# Differential susceptibility to prenatal stress exposure in serotonin transporter-deficient female mice—an epigenetic exploration

**DOI:** 10.3389/fnins.2025.1633386

**Published:** 2025-09-23

**Authors:** Magdalena T. Weidner, Johanna E. M. Zöller, Catharina S. Hamann, Karla G. Schraut, Muhammad Ali, Roy Lardenoije, Lars Eijssen, Konrad U. Foerstner, Nikita Gorbunov, Tatyana Strekalova, Katharina Domschke, Angelika G. Schmitt-Böhrer, Klaus-Peter Lesch, Nicole K. Leibold, Daniel L. A. van den Hove

**Affiliations:** ^1^Division of Molecular Psychiatry, Center of Mental Health, Department of Psychiatry, University of Würzburg, Würzburg, Germany; ^2^Department of Psychiatry and Neuropsychology, Mental Health and Neuroscience Research Institute (MHeNs), Maastricht University, Maastricht, Netherlands; ^3^Department of Psychiatry and Psychotherapy, University of Freiburg Medical Center, Freiburg, Germany; ^4^Pattern Recognition and Bioinformatics, Department of Intelligent Systems, Delft University of Technology, Delft, Netherlands; ^5^Department of Bioinformatics, Psychiatry & Neuropsychology NUTRIM School of Nutrition and Translational Research in Metabolism, Maastricht University, Maastricht, Netherlands; ^6^Research Center of Infectious Diseases, Institute for Molecular Infection Biology, University of Würzburg, Würzburg, Germany; ^7^Peoples Friendship University of Russia (RUDN University), Moscow, Russia; ^8^German Center for Mental Health (DZPG), Partner Site Berlin/Potsdam, Berlin, Germany

**Keywords:** serotonin transporter (5-HTT), prenatal stress (PS), mRNA expression profiles, serotonin transporter deficiency, epigenetic effects of prenatal stress, mouse model

## Abstract

**Introduction:**

Early-life stress exposure has been linked to an increased risk for the onset of mental disorders. As another factor, an individual’s genetic make-up may also tip the scale towards health or disease, with certain genetic variants mediating differential susceptibility towards the effects of early-life stress or other experiences. The current study investigated the molecular foundation of individual variation in the response to prenatal stress (PS) exposure in wildtype mice as well as in mice deficient for the serotonin (5-hydroxytryptamine, 5-HT) transporter (5-HTT).

**Methods:**

To meet this end, wildtype C57BL6/J dams were mated with 5-Htt+/- C57BL6/J males, after which they were exposed to restraint stress during the last part of gestation. In adulthood, female 5-HTT-deficient PS offspring and their wildtype littermates as well as age-matched control groups completed a behavioural test-battery, after which gene expression and histone 3 lysine 4 tri-methylation (H3K4me3) enrichment, as a marker of epigenetic programming, were measured in hippocampal tissue.

**Results:**

Dependent on the 5-Htt genotype, PS offspring showed decreased social behaviour in the 3-chamber sociability test. Notably, we observed a considerable degree of behavioural variation in the observed effect of PS in this social test, which allowed segregation into socially affected (SA) and socially unaffected (SU) mice. Genome-wide mRNA expression profiling of hippocampal tissue revealed a core set of 23 genes to be associated with genotype-specific variation in social behaviour following PS exposure. Whereas H3K4me3 levels did not show profound global changes in relation to the variable effects of PS exposure on social behaviour, the kinesin family member 14 (Kif14) gene, which displayed increased expression in socially unaffected wildtype mice, did show lower levels of H3K4me3 in those same mice, but not in any of the other groups.

**Discussion:**

All in all, differential susceptibility linked to PS exposure displayed 5-Htt genotype-dependent behavioural and transcriptomic profiles, supporting the notion of 5-HT-dependent developmental programming.

## 1 Introduction

Harmful events during development are known to increase the susceptibility for mental illness. Along this line, maternal mental health problems including perceived levels of anxiety during pregnancy have been shown to increase the probability of offspring developing mental issues in later life (Glasheen et al., 2013; Betts et al., 2015). Acute stressful events (Li et al., 2010; Class et al., 2014) and increased maternal cortisol during pregnancy (Buss et al., 2012; Davis and Sandman, 2012) were shown to be associated with similar consequences.

Linking to findings like these, the match/mismatch hypothesis emerged, which proposes that adversity in early life prepares an individual for similar conditions later in life (Schmidt, 2011; Homberg, 2012). If a mismatch occurs between early and later-life contexts, vulnerability to psychiatric disorders is increased. This hypothesis has gained empirical support. Another hypothesis is the three-hit model that proposes that vulnerability arises from the interaction of key aspects, i.e., genetic predisposition, early-life environment, and later-life environment (Daskalakis et al., 2013) When an individual is genetically predisposed (hit 1), and experiences early- (hit 2) and later-life (hit 3) stressors, vulnerability to psychopathology is enhanced. In that case, the stressors accumulate over someone’s lifespan and eventually exceed a certain threshold, potentially triggering the development of psychopathology. The validity of this concept has been demonstrated in animal studies (Farkas et al., 2017; Gaszner et al., 2022a).

In line with these frameworks, next to external factors, genetic predisposition has been shown to be a relevant factor in modulating mental health and an individual’s response to disturbance throughout life. In particular, the serotonergic neurotransmitter system garnered much attention in this context (Booij et al., 2015). Serotonin (5-hydroxytryptamine, 5-HT)-related polymorphisms such as the serotonin transporter (5-HTT, Slc6a4) linked polymorphic region (5-HTTLPR; Caspi et al., 2003, Karg et al., 2011, Schiele et al., 2016) and a monoamine oxidase A (MAO-A) variable number of tandem repeat (VNTR; Caspi et al., 2002) have been suggested to modulate the effect of early adversity. The 5-HTTLPR and polymorphisms in the gene encoding tryptophan hydroxylase 2 (TPH2), which is crucial in 5-HT synthesis, have been associated with altered amygdala reactivity to negative stimuli (Canli et al., 2005; Hariri et al., 2002). Although such gene x environment interactions have been extensively studied, their validity has been disputed (Risch et al., 2009; Culverhouse et al., 2018), which may be due to technical discrepancies between studies and the context-specific nature of these interactions. More specifically, the involvement of additional external and internal factors can affect the experimental outcome, i.e., promote successful adaptation to stress, such as social coping as well as an altered perception and appraisal of situations (Feder et al., 2009).

One major factor regarding stress susceptibility is sex. Among others, van den Hove et al. (2011) showed an interaction of sex and 5-Htt genotype with male *5-Htt +/−* mice showing lower baseline stress hormones and female *5-Htt +/−* mice higher baseline activation of the stress axis, when compared to wildtype offspring. In accordance with this observation, they also found that in particular female *5-Htt +/−* mice displayed increased despair-like behaviour in the Porsolt’s swim test. Similar observations were also reported in humans (Wüst et al., 2009). In addition, the behavioural findings in *5-Htt+/−* females was associated with a distinct DNA methylation and gene expression profile in the hippocampus (Schraut et al., 2014; van den Hove et al., 2011). The hippocampus is one of the brain regions most prominently involved in regulating affective behaviours and anxiety modulation (Shi et al., 2023). For instance, chronic stress has been shown to decrease the expression of plasticity-related genes in the hippocampus (Lakshminarasimhan and Chattarji, 2012). Studies investigating the role of the hippocampus regarding innate anxiety revealed regulatory functions in this respect (Trent and Menard, 2010; McEown and Treit, 2013; Zhang et al., 2014; Bertagna et al., 2021). Evidence from studies with 5-HTT-deficient mice and rats suggests that 5-HT represents a modulatory interface with the capacity to alter the outcome of environmental variation by modulating the epigenetic landscape (Homberg and van den Hove, 2012). This has been investigated in particular in the hippocampus. For example, several studies investigating molecular effects of maternal licking and grooming were able to show that, via increased 5-HT turnover (Hellstrom et al., 2012), epigenetic markers of active gene expression like histone 3 lysine 4 tri-methylation (H3K4me3) were increased at stress-related genes such as the gene encoding the glucocorticoid receptor (GR) in the hippocampus (Zhang et al., 2013; Weaver et al., 2007). H3K4me3 peaks have mainly been reported at the transcription start site of active genes, and changes in H3K4me3 levels have been associated with profound changes in neuronal gene expression (Södersten et al., 2018; Shen et al., 2014). Beyond its modulatory role, the 5-HT system itself is susceptible to environmental stimuli and can display persistent alterations in response to experiences throughout early development (Gardner et al., 2009a; Gardner et al., 2009b). This suggests that, next to direct effects, genetic modification of the 5-HT system can potentiate or buffer individual experiences via epigenetic processes.

Despite a vast body of work regarding the interaction of genetic predisposition within the 5-HT system and early adversity, to the best of our knowledge, no study attempted to investigate the molecular status concomitant with variation in behavioural responses to PS exposure in the context of serotonergic dysregulation. As such, the current study aims to address this complex interaction by making use of an intricate study design. Accordingly, we employed a set of mood- and anxiety-related behavioural tasks to identify behavioural changes related to PS exposure in wildtype and 5-HTT-deficient mice. We deliberately opted for experimental groups with a relatively high number of animals to further explore the considerable degree of behavioural variation and the concomitant genomic profiles that we observed in a previous study with a similar experimental setup (Jakob et al., 2014) to allow for the segregation of PS offspring in affected and unaffected groups. Subsequently, hippocampal tissue of these mice was analysed using genome-wide mRNA sequencing in order to identify genes associated with the observed behavioural changes. Finally, we examined H3K4me3 enrichment by means of chromatin immunoprecipitation (ChIP) sequencing to determine the involvement of this epigenetic mark on the gene expression profile.

## 2 Materials and methods

### 2.1 Animals and procedures

The study was approved by the district government of Unterfranken (Würzburg, Germany; permit number: 55.2–2531.01-93/12), and all efforts were made to minimise animal numbers and suffering. All experiments were performed in accordance with the European Parliament and Council Directive (2010/63/EU). Mice of the parental generation were housed under 14 h/10 h light–dark cycle with lights on at 7 AM–9 PM, at 21 ± 1°C, with a humidity of 45–55%. Standard rodent chow and water were available ad libitum. For breeding, male 5-Htt+/− mice (B6.129(Cg)-Slc6a4tm1Kpl/J; ZEMM, breeding facility, Würzburg; Bengel et al., 1998) were put together with two C57BL6/J female mice (Charles River, Sulzfeld, Germany) each. From that moment onwards, females were tested for vaginal plugs daily. Following a positive plug control, referred to as embryonic day (E) 0 (0), females were housed individually and body weight of pregnant dams was assessed at E0, E13 and E17 to confirm the progressing pregnancy and potential effects of PS (Supplementary Table SI7). Behavioural profiling was done in PS offspring, i.e., in 20 control animals of each genotype and 36 *5-Htt+/+* and 42 5*-Htt+/−*. To prevent litter effects, no more than two female pups per genotype per litter were used (Chapman and Stern, 1979).

### 2.2 Prenatal stress exposure

From E13 to E17, a subset of the pregnant females was subjected to restraint stress for 45 min, three times per day, during the light phase, with an interval of 2–4 h in between sessions. The stress paradigm consisted of restriction in a 25 cm-high, 250 mL glass cylinder filled up to a height of 5 mm with water (hand-hot), whilst exposing them to bright light, as described by Behan et al. (2011). Control animals were left undisturbed in their home cages. Following the last stress session and concomitant weighing of the dams on E17, dams and their respective litters were left undisturbed. Pups were weaned at postnatal day (P) 26 and weighted on P35 and P60. Female offspring that were subsequently tested behaviourally were housed in groups of 3 ± 1 under an inverted 12 h/12 h light–dark cycle (lights on from 7 PM) from P28 ± 1 onwards at the behavioural test unit. Animals were allowed to grow up undisturbed except for weekly cage changes. At approximately 9 weeks of age, behavioural testing started.

### 2.3 Behavioural profiling

All behavioural tests were performed in the dark-phase between 9 AM and 7 PM. Behavioural tests were performed, starting with the Elevated Plus Maze (EPM), followed by the Porsolt Swim Test (PST), the Sucrose Preference Test (SPT) and 3-Chamber Sociability Test (3-CST), with several days in between the various tests in order to minimise carry-over effects. For the EPM, mice were tracked using infrared light from below the apparatus. For all tests, trials were recorded from above, using an infrared-sensitive camera. Behavioural analysis was performed using VideoMot2 tracking software (TSE Systems, Bad Homburg, Germany) and Ethovision Pro software (Noldus, Wageningen, The Netherlands). In-between trials, the respective apparatus was cleaned with Terralin liquid (Schülke, Norderstedt, Germany). Twenty control animals of each genotype and 36 5-Htt+/+ and 42 5-Htt+/− PS offspring were tested.

*Elevated plus maze*: The EPM apparatus consists of a plus-shaped acrylic glass construct (TSE Systems, Inc., Bad Homburg, Germany) made from black opaque PERSPEX XT, semi-permeable to infrared light, with two opposing closed arms (30 cm × 5 cm) surrounded by 15 cm high walls and two opposing open arms without walls (30 cm × 5 cm, with 0.5 cm wide boundaries elevated 0.2 cm). The four arms met in the centre to form a square of 5 cm × 5 cm. The maze was raised 62.5 cm above the ground. The test was undertaken under low light conditions. Each animal was placed in the centre facing an open arm and allowed to explore the maze for 5 min. Subsequently, time spent and distance moved in the open arms, the closed arms and the centre, as well as the number of entries into the different arms were analysed (Lister, 1987; Post et al., 2011).

*Porsolt swim test*: For the PST, mice were placed in a 40 cm tall acrylic glass cylinder with a diameter of 19 cm filled up to 15 cm with warm water (31 ± 1°C) for 10 min. Mice from one cage were tested in parallel, in visually separated cylinders. The setup was illuminated from below with a light box. Between trials, the water was renewed. Distance moved was analysed as an indicative parameter of mobility (Behan et al., 2011).

*Sucrose preference test*: The SPT was performed interchanging with the sociability test, in a way that animals had 1 day of rest between tests. Mice were single-housed for 12 h during the dark phase, from 8 AM to 8 PM, and presented with two similar bottles, one filled with regular tap water and the other with 1% sucrose solution. To exclude the influence of a side preference, the sucrose bottle was positioned on the right or left side in an alternating fashion. The bottles were prepared 1 day in advance to ensure that the solutions were at room temperature (RT) and to avoid the use of leaking bottles. All bottles were weighed both before and after the test. Sucrose preference was calculated as the percentage of consumed sucrose solution in view of the total volume consumed (Strekalova et al., 2004).

*3-chamber sociability test*: The 3-CST setup consisted of an acrylic glass structure comprising 3 separate chambers, i.e., a middle chamber with two adjacent chambers of equal size, to be reached through passages. Both side chambers contained a wired cage. Prior to testing, animals were allowed 5 min of habituation in the middle chamber of the apparatus. Following these 5 min, a female conspecific of the same age was placed into one of the small wired cages and the subject was allowed to explore the whole apparatus for 10 min (Kaidanovich-Beilin et al., 2011). Subsequently, the time spent in the respective chambers was assessed manually from video files.

### 2.4 Corticosterone response

To assess the acute corticosterone response, blood was collected from the saphenous vein (legs were shaved 1 day in advance) before (‘baseline’) and immediately after 20 min of restraint stress (‘stress’), 1 week after the last behavioural test. The stress procedure was performed as described for PS. Blood samples were subsequently centrifuged and the plasma stored at −80°C. Plasma corticosterone concentrations were determined using a radioactive immunoassay (RIA), based on previous work (Leibold et al., 2020).

### 2.5 Genome-wide profiling of mRNA expression and H3K4me3 enrichment

Following behavioural testing and assessment of the corticosterone response, animals were sacrificed using isoflurane followed by quick decapitation. Brains were harvested and immediately frozen in isopentane at −80°C. Frozen brains were semi-thawed on a − 6°C cooling plate and the entire hippocampus was rapidly dissected using a stereo microscope (Olympus Europa, Hamburg, Germany). Tissue of the left and right hippocampus was merged, powderised at 80°C and split into two homogenous portions to enable investigating RNA expression and epigenetic modifications on the DNA in the same animals.

### 2.6 mRNA sequencing

RNA extraction was performed using the commercial miRNeasy Mini kit (Qiagen, Hilden, Germany) for fatty tissue. RNA was eluted with RNase free H_2_O provided with the kit and stored at −80°C. Before RNA sequencing, RNA quality was assessed on a 1.5% agarose gel with ethidium bromide and verified using the Experion (Biorad, München, Germany) according to the manufacturer’s instructions. RNA concentrations were determined using a spectrophotometer (Nanodrop, Thermo Scientific, Wilmington, Delaware, United States). Only RNA samples with a RNA quality indicator (RQI) value of 8 or higher were used for mRNA sequencing. Eight animals from each group were chosen randomly for mRNA sequencing. Sequencing and all related processes as well as quality control were handled by IGA Technologies (Udine, Italy). For this purpose, single-end sequencing was performed on an Illumina HiSeq2000 sequencer (Illumina, San Diego, California, United States) at a depth of 30 million reads per sample with and a read-length of 50 bp. We then processed the raw fastq files using the bcbio-nextgen pipeline.^1^ In brief, we performed mapping with the STAR aligner and the mm10 genome build, mostly with default settings, using STAR quality metrics (Dobin et al., 2013), QualiMap (Okonechnikov et al., 2016) and FastQC (Andrews, 2010), combined with MultiQC (Ewels et al., 2016) for quality control. For quantitation of gene-level counts we used Salmon (Patro et al., 2017) and tximport (Soneson et al., 2015). Sequencing data have been deposited in the Gene Expression Omnibus database (GSE109929).

### 2.7 H3K4me3 chromatin-immunoprecipitation sequencing

Powderised hippocampal tissue was mildly fixed using 1% formaldehyde. The fixation was stopped after 10 min by incubating the samples with 0.125 M glycine for 10 min at RT. All following steps were performed in solutions containing protease inhibitors (Roche, Basel, Switzerland). Following quenching, the samples were washed, subsequently grinded in SDS-lysis buffer [1% SDS, 10 mM EDTA, 50 mM Tris–HCl (pH 8.1)] and incubated on ice for 10 min. Sonication was performed at 4°C in ice water using the Biorupter UCD-200 (Diagenode, Liège, Belgium), at the low intensity setting for 2 × 10 min with a pulse interval setting of 0.5. To test shearing success and to determine the DNA concentration, DNA was extracted from a portion of each sample as described below. Following DNA extraction, fragment size was determined using the Bioanalyzer 2,100 (Agilent, Santa Clara, California, United States) and the DNA concentration was measured using the Qubit dsDNA HS Assay kit. In case of successful shearing, 1,000 ng/antibody was transferred to a fresh 2 mL tube, filled with chromatin-immunoprecipitation (ChIP) dilution buffer (0.01% SDS, 1.1% Triton X-100, 1.2 mM EDTA, 16.7 mM Tris–HCl, pH 8.1, 167 mM NaCl, 1x protease inhibitor cocktail) up to the volume of 1 mL, per sample. Subsequently, samples were precleared using herring-sperm-DNA-blocked protein A agarose beads for 30 min at 4°C on a rotor. Next, the samples were incubated overnight at 4°C with the respective primary antibody (H3K4me3: 39159, Active motif, Carlsbad, CA, United States; rabbit IgG: X090302, Vector Laboratories, Burlingame, CA, United States) on a rotor. After incubation, antibodies were captured by using herring-sperm-DNA-blocked protein A agarose beads for 2 h at 4°C on a rotor. Following antibody capture, the beads were washed for several times, with the following buffers in the listed order: Low Salt Buffer (0.1% SDS, 1% Triton X-100, 2 mM EDTA, 20 mM Tris–HCl, pH 8.1, 150 mM NaCl), High Salt Buffer (0.1% SDS, 1% Triton X-100, 2 mM EDTA, 20 mM Tris–HCl, pH 8.1, 500 mM NaCl), LiCl Buffer [0.25 M LiCl, 1% IGEPAL-CA630, 1% deoxycholic acid (sodium salt), 1 mM EDTA, 10 mM Tris, pH 8.1] and two times with 1x TE buffer, after which the antibodies with the captured DNA were eluted from the beads, using elution buffer (0.1 M NaHCO3, 1% SDS). Subsequently, the samples were de-cross-linked, using 0.2 M NaCl at 65°C overnight. Genomic DNA was isolated using phenol/chloroform/isoamyl alcohol extraction. Additionally, one sample per group was used as an input control (IN) that was not subjected to immunoprecipitation as positive control. Another sample was immunoprecipitated with a mixture of random rabbit IgGs (RAB, Vector Laboratories, Burlingame, CA, United States), as negative control. For the IN fraction, 100 μL were taken from the RAB samples. Immunoprecipitated (IP) DNA was stored at −80°C for further use and a fraction was used for quantification using the Qubit fluorometer (Thermo Fisher Scientific, Waltham, MA, United States) with the Qubit high sensitivity kit for double-stranded DNA (Thermo Fisher Scientific, Waltham, MA, United States). Furthermore, specificity of the immunoprecipitation was investigated using qPCR, with Afamin (Afm) as negative control and glyceraldehyde-3-phosphate dehydrogenase (Gapdh) as positive control (Supplementary material SI1). Both primer pairs were located within the promoter region of the respective gene (Supplementary materials SI2, SI3).

Six samples per group were chosen based on concurrent mRNA expression profiling (Supplementary material SI4), if sample quality allowed, and sent to Nxt-Dx (Ghent, Belgium), who performed the sequencing. Library preparation was performed using the NEBNext Ultra II DNA Library prep kit for Illumina (NEB, Ipswich, Massachusetts, United States). Subsequently, the whole IP material was subjected to ends prep and ligation of Illumina adaptors. A clean-up of the adaptor-ligated DNA with AMPure XP beads (Beckman Coulter) was performed without size selection. The eluted material was subjected to enrichment PCR (14 cycles) with NEBNext Index primers, followed by clean up with AMPure XP beads. The quality of the final libraries was checked on a Bioanalyzer 2,100 DNA 1000 chip (Agilent, Santa Clara, CA, United States). The concentration was determined by performing quantitative polymerase chain reaction (qPCR) on the samples using a dilution of PhiX index3 as standard. The concentration of all indexed samples was adjusted to 10 nM and samples were pooled for sequencing. Sequencing was performed on an Illumina HiSeq4000. All samples were processed at a read-length of 50 bp with 25–30 million reads/sample, paired-end sequencing. Follow-up data demultiplexing, clean up and quality control were performed by Nxt-dx. Subsequently, paired end 50 bp sequence reads were mapped to the *mus musculus* GRCm38.p5 genome using Bowtie2 (v2.3.2.). Coverage peaks were generated using the MACS 2 (v2.1.1) peak caller. Following peak calling, peaks were annotated using the Homer software (v4.9). Quality control results are summarised in Supplementary material SI5. It should be mentioned that mapping efficiency was lower than expected for some of the samples and, while no technical issues or group bias could be detected, results should be interpreted with caution in this respect (Supplementary material SI6). Sequencing data have been deposited in the Gene Expression Omnibus database (GSE109928).

### 2.8 Statistical analysis

*Behaviour:* For statistical analysis of behavioural and physiological measures, SPSS Statistics 26 (IBM Deutschland GmbH, Ehningen, Germany) was used. Due to leaking water bottles, the amount of sucrose consumed could not be determined in three animals, which were excluded from analyses. In the EPM, two mice were excluded due to recording failure. To test for main effects or interactions of 5-Htt genotype and PS, we performed a two-way ANOVA and repeated measures ANOVA, where applicable. Data were examined for normal distribution, outliers, variance distribution and sphericity, using the Shapiro–Wilk test, boxplots, the Leven’s Test and Mauchly’s test, respectively. If assumptions for parametrical testing were not met, additional non-parametric analysis, i.e., Mann–Whitney-U and Kruskal-Wallis tests, were performed. If the results of the ANOVA analysis were confirmed, ANOVA results are reported in the main text. Results of non-parametric tests can be found in Supplementary material SI13. *p*-values < 0.05 were considered significant.

**Figure 1:**
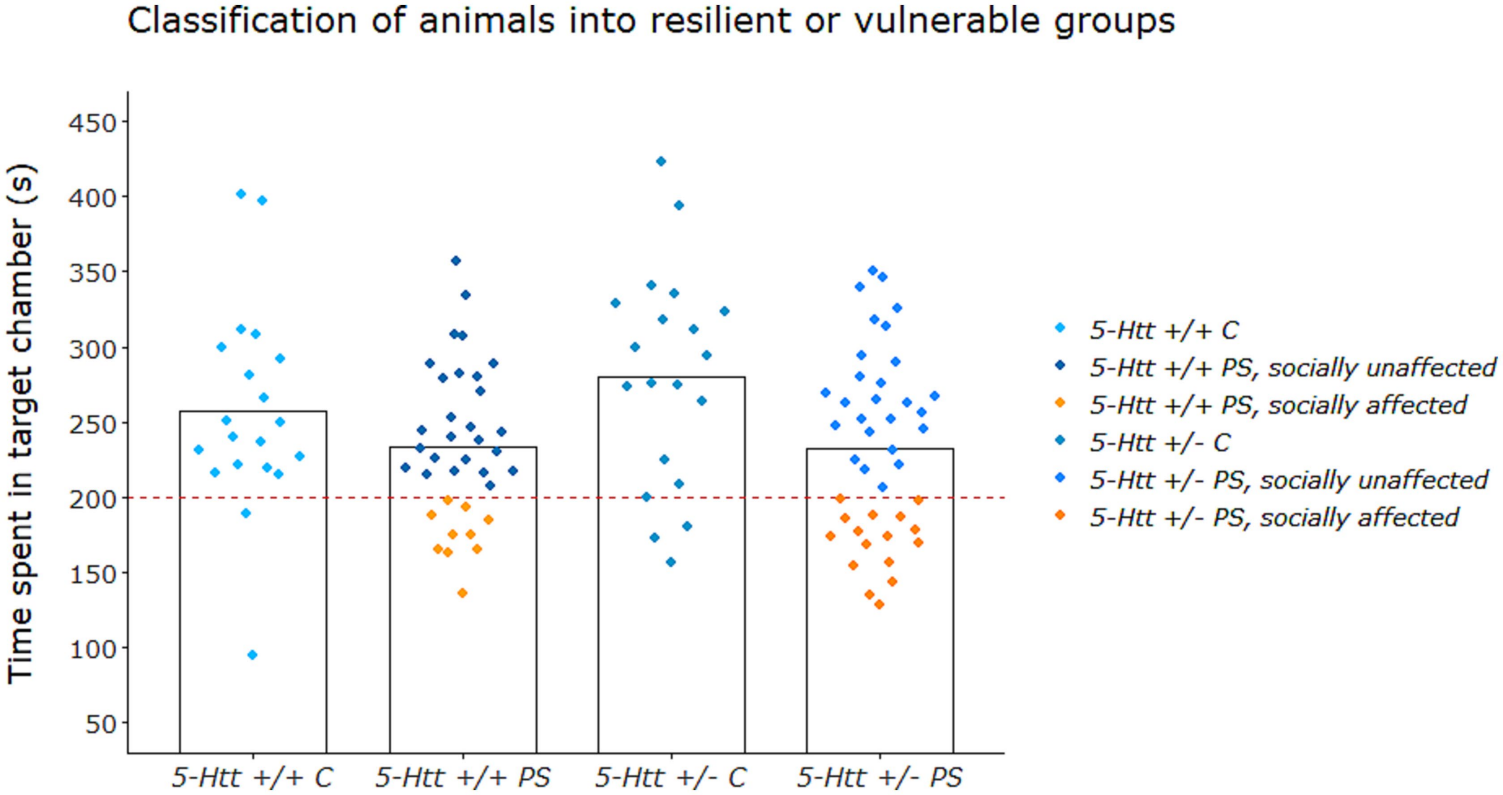
Segregation into socially affected (SA) and unaffected (SU) groups based on performance in the 3-chamber sociability test (3-CST). The performance in this test was used to segregate PS offspring into affected and unaffected animals, as previously described (Berton et al., 2006). Based on the observation that a great majority of control offspring spent more time than chance level (>200 s) in the social target chamber, a cut-off at 200 s (dashed line) was determined to indicate intact social behaviour. Thus, PS offspring that spent less than 200 s in the social target chamber were termed socially affected (SA, orange) and PS offspring that spent more than 200 s in the social target chamber were termed socially unaffected (SU, blue). Figure adapted from Schraut, 2015.

*Segregation of PS offspring in groups affected and unaffected regarding social behaviour:* Following this first analysis, behavioural measures that displayed a significant PS effect were investigated with a focus on interindividual variability. Of all behavioural tests, in particular the EPM and the 3-CST showed effects of PS (see Supplementary Table 2 in results section). While in the EPM animals that were exposed to PS showed decreased anxiety-related behaviour when compared to controls, animals exposed to PS showed overall decreased social behaviour in the 3-CST. When further exploring these data, we observed a considerable degree of variation in the effect of PS on the performance in the 3-CST. As such, the performance in this test was used to segregate PS offspring into affected and unaffected animals, as previously described (Berton et al., 2006). In detail, the majority (67%) of PS offspring showed a preference for the social target chamber similar to offspring of the control group (Figure 1). Based on the observation that a great majority of control offspring spent more time than chance level (>200 s) in the social target chamber, a cut-off at 200 s was determined to indicate intact social behaviour. Thus, PS offspring that spent less than 200 s in the social target chamber were termed socially affected (SA) and PS offspring that spent more than 200 s in the social target chamber were termed socially unaffected (SU).

To zoom in on the potential implications of the observed interindividual variability in social behaviour, the initial analyses on the effects of PS exposure on behaviour were complemented by analyses that integrated this segregation based on performance in the 3-CST. Group comparisons were performed as described above with 3-CST performance instead of PS exposure as fixed factor. *Post hoc* analyses for interaction effects were Bonferroni-corrected for four group comparisons, i.e., 5-Htt+/+SA vs. 5-Htt+/+C; 5-Htt+/+SU vs. 5-Htt+/+C and 5-Htt+/-SA vs. 5-Htt+/-C, 5-Htt+/-SU vs. 5-Htt+/-C. In addition, the association between behaviours was investigated using Pearson’s correlation coefficient (r).

*Differentially expressed genes (DEGs)*: Statistical analyses of RNA expression and H3K4me3 enrichment data were all performed in R (version 3.6.1). The analysis for the identification of DEGs was carried out using the edgeR package (version 3.26.8). Briefly, gene expression data were normalised using default parameters where genes having less than five reads in a minimum of three samples were filtered out. Furthermore, the “upper quartile” normalisation method was used to normalise raw expression values and the empirical Bayes method was used to shrink gene-wise dispersion estimates towards a common dispersion. For statistical analysis, we fitted a negative binomial generalised log-linear model (GLM) to the processed read counts for each gene and conducted the gene-wise statistical tests for a coefficient contrast to identify DEGs. We calculated the contrast which describes a three-way interaction of 5-HTT deficiency, PS exposure and socially affected/unaffected behaviour (5-HTT*PS*socially affected/unaffected behaviour) [(5-Htt+/+SA vs. 5-Htt+/+C) vs. (5-Htt+/+SU vs. 5-Htt+/+C)] vs. [(5-Htt+/-SA vs. 5-Htt+/-C) vs. (5-Htt+/-SU vs. 5-Htt+/-C)]. Based on the identified nominally significant DEGs, we performed the following *post hoc* analyses: [(5-Htt+/+SA vs. 5-Htt+/+C) vs. (5-Htt+/+SU vs. 5-Htt+/+C)] and [(5-Htt+/-SA vs. 5-Htt+/-C) vs. (5-Htt+/-SU vs. 5-Htt+/-C)], which describes a two-way interaction of PS exposure and socially affected/unaffected behaviour, stratified per 5-Htt genotype, and [5-Htt+/+SA vs. 5-Htt+/+C], [5-Htt+/+SU vs. 5-Htt+/+C], [5-Htt+/-SA vs. 5-Htt+/-C] and [5-Htt+/-SU vs. 5-Htt+/-C], which describe the effects of PS exposure on gene expression in SA and SU offspring each, stratified per 5-Htt genotype. We used the Benjamini-Hochberg (False Discovery Rate, FDR) method to adjust *p*-values for multiple testing. In the three-way interaction we identified 1,484 nominally significantly differentially expressed genes. As subsequent analyses were restricted to these comparisons, multiple testing correction was restricted to this set of genes. The genomic inflation factor lambda was 0.999, indicating no inflation of the test statistics.

*Gene ontologies (GO)*: a gene set-based enrichment analysis of biological processes (BP), and molecular functions (MF) was carried out for all the nominal DEGs (*p* < 0.05) using the clusterProfiler package (version 4.12.0).

*Differentially enriched fragments* (*DEFs,* i.e.*, gene fragments enriched for H3K4me3*): in order to identify regions enriched for H3K4me3 histone marks associated to nominal DEGs (*p* < 0.05), we used the DiffBind package (version 2.12.0). Briefly, the peak data and the sequencing alignment files related to the 1,484 nominal DEGs were used to calculate a binding matrix with scores based on read counts for every sample. Subsequently, a DESeq2-based (version 1.36.0) approach was used on the default binding matrix to identify regions enriched for H3K4me3, i.e., DEFs. The model used for identifying DEFs was similar to the one used for the differential gene expression analysis and the Benjamini-Hochberg method was used to correct p-values for multiple testing.

## 3 Results

### 3.1 *5-Htt*-dependent behavioural performance following PS exposure

An overview of all effects of PS, 5-Htt genotype and their interaction on behaviour, can be found in Supplementary Table 2. As described above, animals exposed to PS did not just show reduced sociability overall, but also displayed a considerable degree of interindividual variability in social behaviour covering a range from ‘normal, socially unaffected’ (SU) to ‘decreased, socially affected’ (SA) behaviour (see Figure 1; Supplementary Table 2).

**Figure 2:**
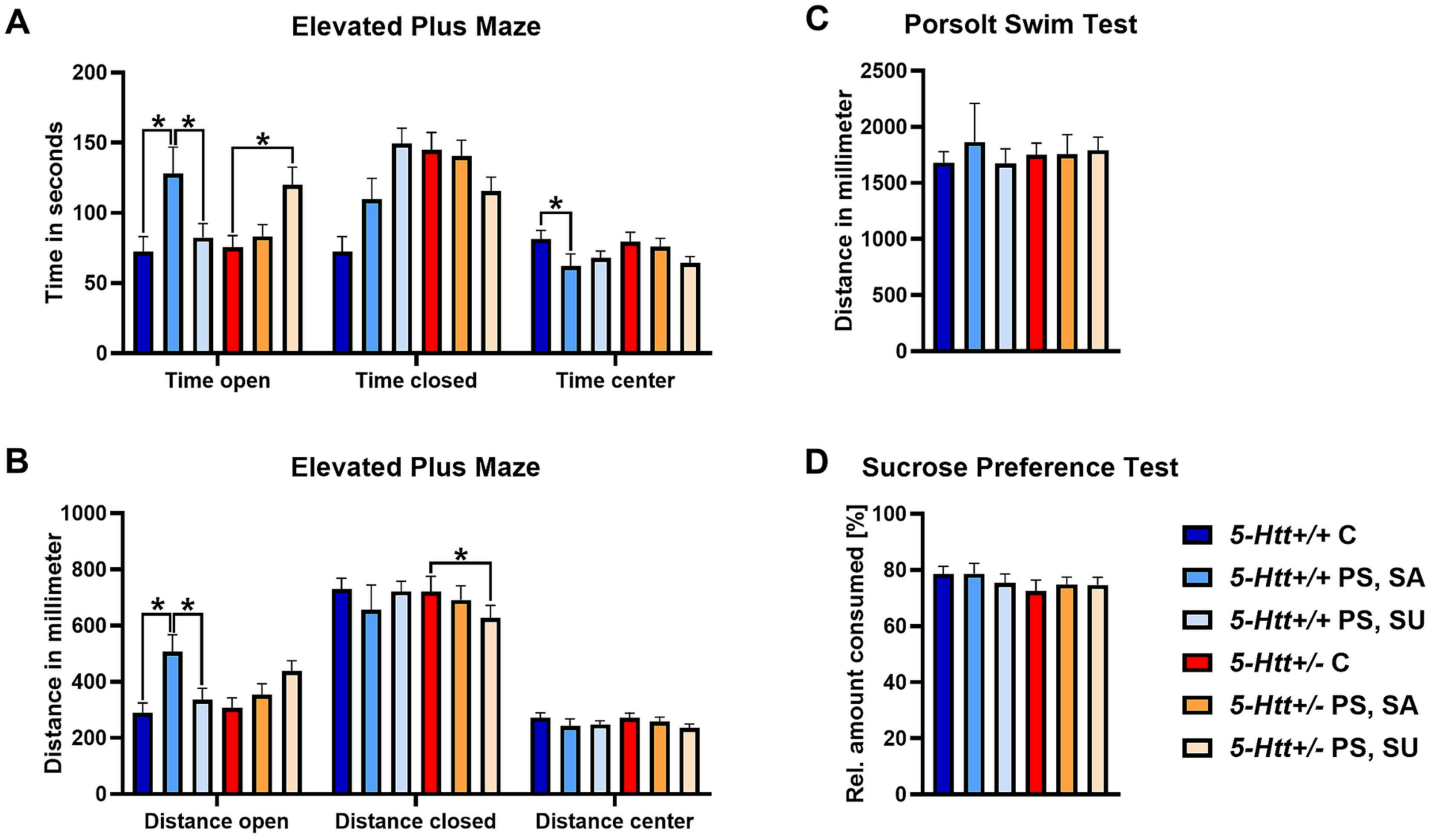
Results of the behavioural analysis based on performance in the 3-chamber sociability test. Time spent in the open and closed arms, as well as the centre of the Elevated Plus Maze **(A)**. Distance covered in the open and closed arms, as well as the centre of the Elevated Plus Maze **(B)**. Distance covered in the Porsolt Swim Test **(C)**. Relative sucrose amount consumed in the Sucrose Preference Test **(D)**. Data represent mean ± SEM. p* < 0.05. C, control; SA, socially affected; SU, socially unaffected. Figure adapted from Schraut, 2015.

More specifically, in terms of genetic variation, overall, we did not detect any significant differences between *5-Htt*+/+ and *5-Htt+/−* mice with regard to any of the behavioural parameters assessed. Regarding PS exposure, we observed a significant effect on behaviour in the 3-CST, with PS offspring spending less time in the chamber with the social target (*F*(1.114) = 8.81, *p* = 0.004). In the EPM, PS exposure was associated with an increased time spent (*F*(1.112) = 6.6, *p* = 0.012) and distance moved (F(1.112) = 7.0, *p* = 0.009) in the open arms. We did not observe any effects of an interaction between 5-Htt genotype and PS. Interestingly, when dividing offspring into SU and SA animals, based on their social behaviour, we found that differential social behaviour following PS exposure was associated with altered time spent (*F*_(2,110)_ = 5.68, *p* = 0.004) and distance covered (F_(2,110)_ = 4.29, *p* = 0.016) on the open arms as well as time spent in the centre (*F*_(2.110)_ = 3.52; *p* = 0.033) of the EPM. This was dependent on the *5-Htt* genotype. More specifically, *5-Htt*+/+ SA offspring spent more time and covered a greater distance on the open arms, when compared to *5-Htt*+/+ control offspring (C) (p = 0.009 and *p* = 0.006) and *5-Htt*+/+ SU offspring (*p* = 0.044 and *p* = 0.026). Additionally, *5-Htt*+/+ SA offspring spent less time in the centre when compared to *5-Htt*+/+ C offspring (*p* = 0.048). In *5-Htt*+/− animals, in contrast, SU offspring spent more time on the open arm (*p* = 0.026) and covered a greater distance (*p* = 0.012) on the open arms when compared to undisturbed control offspring. Furthermore, correlation analysis revealed a negative correlation between the time spent in the chamber with the social target and time spent (*r* = −0.477, *p* < 0.001) and distance covered (*r* = −0.405, *p* = 0.002) on the open arm of the EPM in *5-Htt+/+* offspring. No correlation was found for *5-Htt*+/− offspring. We did not observe any association of the interindividual variability in 3-CST performance with the distance covered in the PST or sucrose preference. A graphical representation of the results of the behavioural tests can be found in Figure 2.

Although we did assess effects of either PS exposure, *5-Htt* genotype and their interaction as well as differential social behaviour following PS exposure *in utero* and its interaction with *5-Htt* genotype on physiological parameters like corticosterone concentration, none of the results were significant (Supplementary material SI12).

### 3.2 Differential gene expression dependent on 5-HTT deficiency and interindividual variability in social behaviour following prenatal stress exposure

Given the distinct behavioural profile of SA and SU offspring and the role of 5-HTT deficiency in this context, we next determined expression profiles of each of the groups compared to control (C) offspring. For this purpose, we calculated the contrast (for details see material & methods section).

Our analysis identified 1,484 nominally significant differentially expressed genes (DEGs), 23 of which remained significant after correcting for multiple testing (see Supplementary material SI8; Table 1, respectively).

**Table 1:** Statistical analysis of differentially expressed genes (DEGs) in the context of a three-way interaction of serotonin transporter (5-HTT) deficiency, PS and differential sociability (5-HTT*PS*socially affected/unaffected behaviour) by means of the calculated contrast: [(5-*Htt+/+*SA vs. *5-Htt+/+*C) vs. (*5-Htt+/+*SU vs. *5-Htt+/+*C)] vs. [(*5-Htt+/-*SA vs. *5-Htt+/-*C) vs. (*5-Htt+/-*SU vs. *5-Htt+/-*C)].

Gene_symbol	Ensembl_ID	logFC	logCPM	LR	*p* value	FDR
Ccl21c	ENSMUSG00000096271	−12.65	−2.14	32.94	9.49E+05	0.0003
Gm22623 retired under this ID	ENSMUSG00000094452	−11.26	−0.67	29.98	4.37E+06	0.001
Gm6685	ENSMUSG00000032889	−7.42	−0.22	28.69	8.50E+06	0.001
Gm9847	ENSMUSG00000050974	9.04	−3.52	28.21	1.09E+07	0.001
Eef1ece2	ENSMUSG00000115293	−10.56	−1.92	28.16	1.12E+07	0.001
Gm47184	ENSMUSG00000113664	−10.44	−2.79	26.57	2.54E+07	0.001
Rpl3-ps1	ENSMUSG00000084349	10.91	−1.41	26.29	2.94E+06	0.001
Gm7935	ENSMUSG00000075609	−9.72	−0.43	23.26	1.42E+08	0.004
Fam177a2	ENSMUSG00000094103	9.66	−3.33	23.22	1.45E+08	0.004
Kif14	ENSMUSG00000041498	−5.68	1.27	22.86	1.74E+08	0.005
Gm3020	ENSMUSG00000079402	8.83	−3.59	21.96	2.78E+08	0.007
Gm22969 retired under this ID	ENSMUSG00000094229	−8.66	−0.88	19.99	7.77E+08	0.017
Gm21974	ENSMUSG00000030804	8.76	−2.76	19.77	8.75E+08	0.017
Lgals1-ps2	ENSMUSG00000091135	6.06	−2.88	19.75	8.81E+08	0.017
Otop2	ENSMUSG00000050201	−4.13	−0.86	18.20	1.99E+09	0.033
Gm6916	ENSMUSG00000108608	8.36	−1.70	18.17	2.03E+09	0.033
Gm28017 retired under this ID	ENSMUSG00000099322	−8.14	−1.63	18.11	2.09E+09	0.033
Gm4294	ENSMUSG00000078377	7.70	−0.86	17.75	2.52E+09	0.037
Saxo1	ENSMUSG00000028492	−6.53	−3.89	17.18	3.40E+08	0.045
Gm8203	ENSMUSG00000101878	7.67	−0.54	17.15	3.45E+08	0.045
Mapk15	ENSMUSG00000063704	1.97	−0.28	17.07	3.60E+09	0.045
Gm43335	ENSMUSG00000106623	−2.38	−2.67	17.01	3.73E+09	0.045
Gm16033	ENSMUSG00000086998	7.69	−2.51	16.77	4.23E+09	0.049

**Figure 3:**
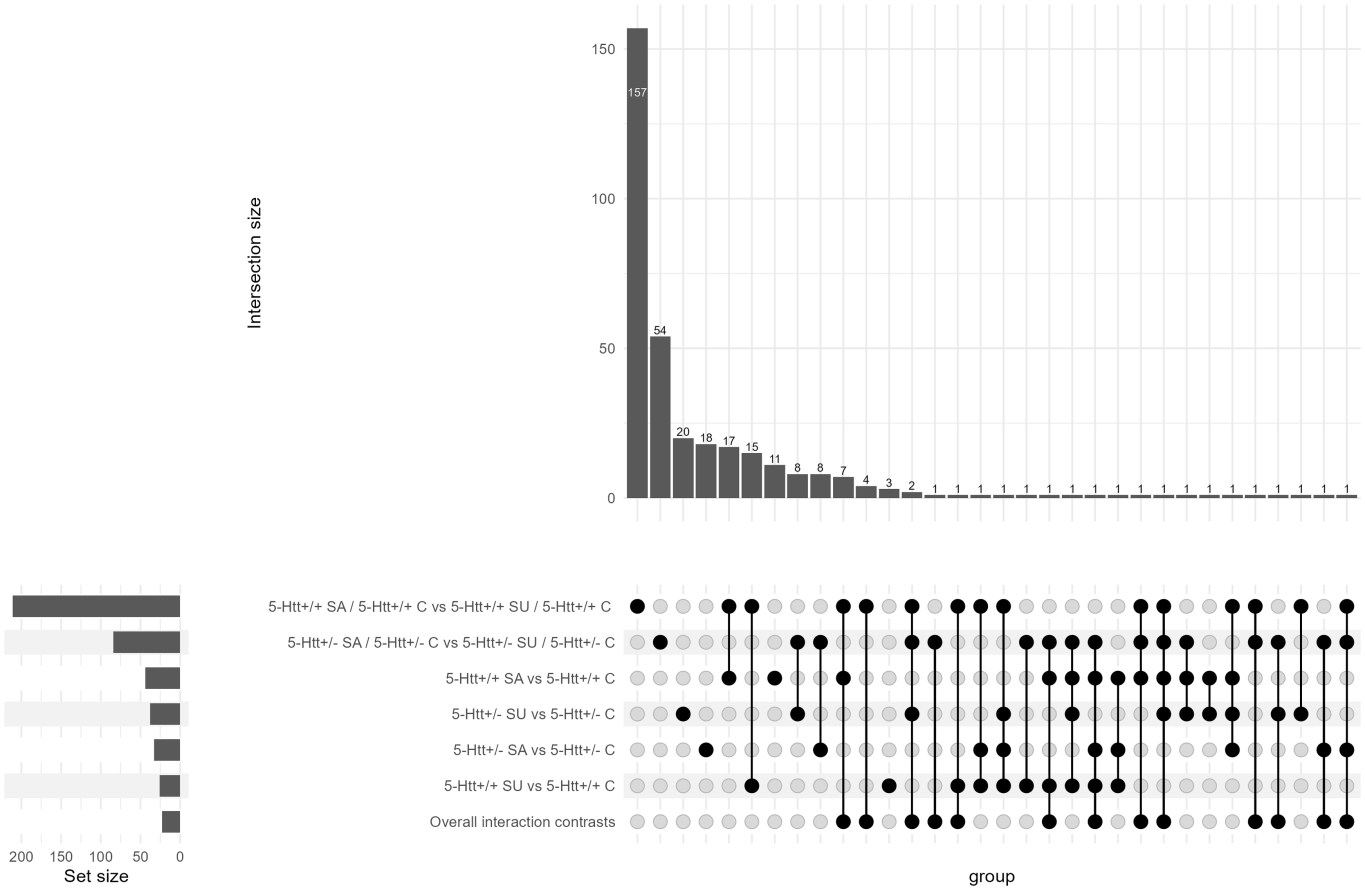
UpSet plot visualising the overlap of differentially expressed genes (DEGs) identified across multiple contrasts. The contrasts include: (1) the three-way interaction between 5-Htt deficiency, PS exposure and socially affected/unaffected behaviour (5-Htt × PS × SA/SU); (2) the two-way interaction of PS exposure and socially affected/unaffected behaviour, stratified by 5-Htt genotype; (3) the main effect of PS exposure on gene expression in SA and SU offspring, each stratified by 5-Htt genotype. The number of significant DEGs identified in each contrast is: 23, 211, 84, 44, 26, 33, and 38, respectively. Each bar in the plot represents the number of DEGs uniquely or jointly identified in the contrasts indicated by the connected dots below. SU, socially unaffected; SA, socially affected; C, control; 5-Htt, serotonin transporter; DEG, differentially expressed gene (Supplementary material SI9 significant DEGs).

**Figure 4:**
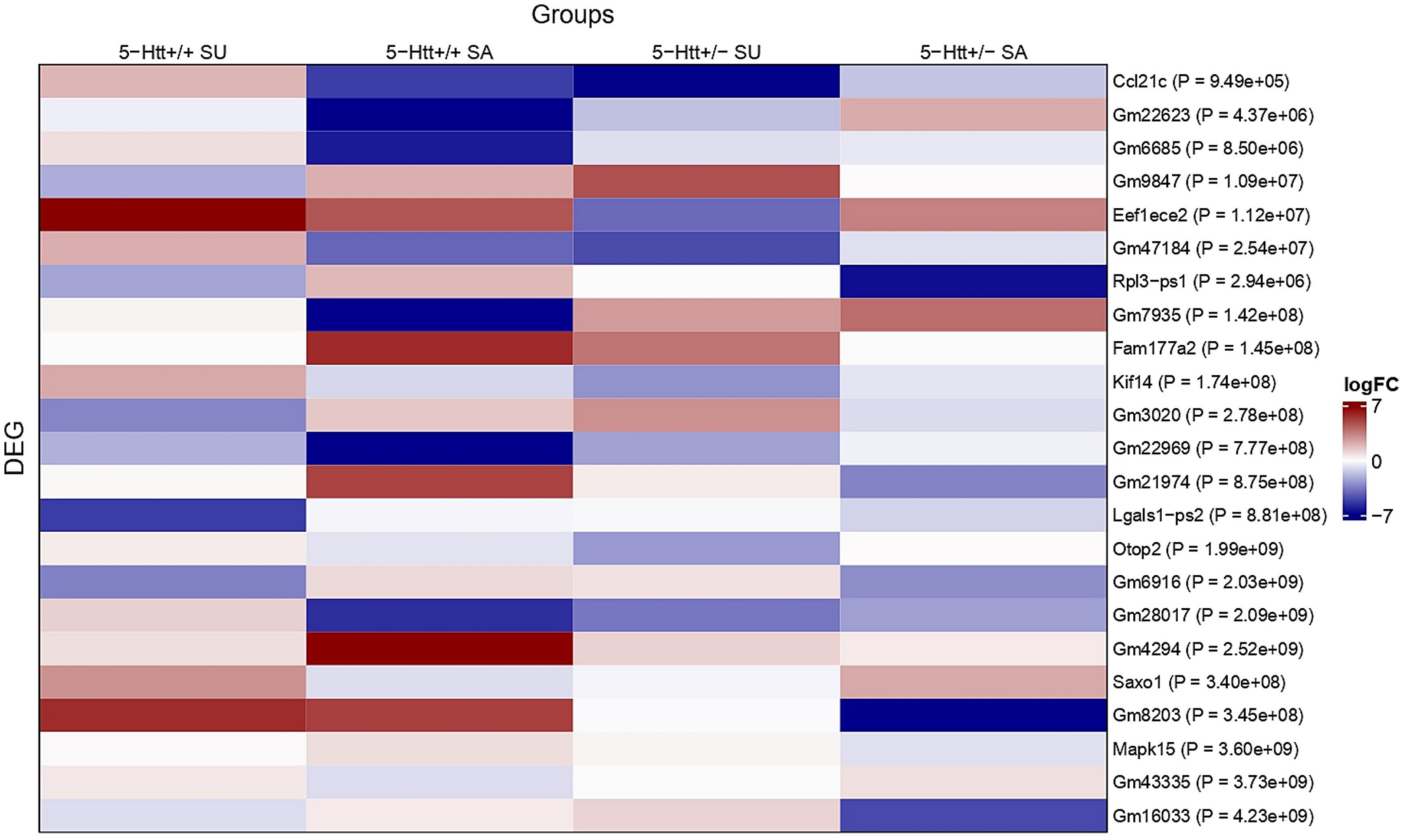
Heatmap presenting the log fold change (logFC) values of differentially expressed genes (DEGs) identified through a three-way interaction. The three-way interaction was calculated between serotonin transporter (5-HTT) deficiency, prenatal stress (PS), and sociability phenotype [socially affected (SA) vs. unaffected (SU)]. The interaction contrast used for DEG identification was: [(5-Htt+/+ SA vs. 5-Htt+/+ C) vs. (5-Htt+/+ SU vs. 5-Htt+/+ C)] vs. [(5-Htt+/− SA vs. 5-Htt+/− C) vs. (5-Htt+/− SU vs. 5-Htt+/− C)]. Displayed logFC values reflect comparisons between the SA and SU groups and their respective genotype-matched control groups (5-Htt+/+ or 5-Htt+/−). Columns represent the SA and SU subgroups per genotype; rows show the DEGs identified by the interaction, with *p*-values shown next to each gene name.

An UpSet plot describing the overlap of significant DEGs of all investigated contrasts is displayed in Figure 3 (see Supplementary material SI9 for significant DEGs). A heatmap presenting the log fold change (logFC) values of differentially expressed genes (DEGs) identified through a three-way interaction is displayed in Figure 4. For the heatmap showing the expression levels (logCPM) of differentially expressed genes (DEGs) identified through a three-way interaction, see Supplementary material SI10.

All of the 23 significant DEGs of the three-way interaction overlapped with DEGs of other contrasts. The expression of the predicted genes *Fam177a2, Gm22969*, *Gm28017*, *Gm7935*, *Gm4294*, *Gm21974*, *Gm22623* and *Gm6685* was only altered in SA *5-Htt+/+* offspring when compared to corresponding control offspring. *Lgals1-ps2* was only altered in SU *5-Htt+/+* offspring and the expression of *Eef1ece2* was altered in both SA and SU when compared to control offspring. The expression of *Rpl3-ps1* and the predicted gene *Gm16033* were changed in SA 5-Htt+/− offspring, while expression of Otop2 and the predicted genes *Gm47184* and *Gm9847* was altered in SU 5-Htt+/− offspring, when compared to the controls. The expression of *Ccl21b* was changed in SA *5-Htt+/+* and SU *5-Htt*+/− offspring, whereas the expression of the predicted gene *Gm8203* was altered in all but SU 5-Htt+/− when compared to corresponding control offspring. Finally, *Kif14* and the predicted genes *Saxo1*, *Gm43335* and *Gm6916* were altered in expression in *5-Htt+/+* offspring by an interaction of PS exposure and differential sociability. *Mapk15* and the predicted gene *Gm3020* were altered in expression in *5-Htt*+/− offspring by an interaction of PS exposure and differential sociability. For detailed graphs of normalised read counts, see Supplementary material SI11.

Of the 23 DEGs, five were protein-coding genes (*Ccl21b*, *Eef1ece2, Kif14*, *Mapk15* and *Otop2*), three DEGs were pseudogenes (*Fam177a1p1*, *Lgals1-ps2*, *Rpl3-ps1*) and 15 DEGs were corresponding to predicted genes such as ncRNAs, potential protein-coding genes and pseudogenes. Among the DEGs listed as predicted ncRNAs, *Gm16033* is identified as a long ncRNA overlapping with the 3′ end of *A disintegrin and metallopeptidase domain 19* (*Adam19*), *Gm28017* as micro-RNA, and *Gm22623* and *Gm22969* as small nuclear RNAs. Gm22623, Gm22969 and Gm28017 are retired under their respective IDs in the Ensembl database version 112. There were three predicted protein-coding genes: *Stabiliser of axonemal microtubules 1* (*Saxo1*), which is inferred from homology, Gm21974, a transcript with premature stop codon, and *Gm43335*, a region with EST clusters that have polyA features that could indicate the presence of protein coding genes. Furthermore, there were 8 predicted pseudogenes, the processed pseudogenes *Gm4294*, *Gm47184*, *Gm6685*, *Gm7935*, *Gm8203, Gm9847* and *Gm6916*, a transcribed pseudogene with processed transcript and transcribed processed pseudogene. Interestingly, gene ontology (GO enrichment) analysis of the 1,484 nominally significant DEGs found mostly terms with regard to cilia for the top biological processes. A list of the top hits can be found in Table 2.

**Table 2:** Gene ontologies: top 10 hits of gene set-based enrichment analysis for the 1,484 nominally DEGs associated with biological processes and molecular functions.

ID	Description	Gene ratio	Bg ratio	*p* value	p. adjust	*q* value	gene ID	Count
Top 10 hits associated with biological processes								
GO:0003341	cilium movement	21/867	194/17385	0.0007	0.968	0.968	Gas2l2/Mkks/Odad4/Dnai3/Stard7/Mns1/Dnai2/Klc3/Vdac3/Tekt1/Drc1/Iqcg/Ttll1/Cep131/Dnali1/Cfap45/Dnah6/Cfap65/Drc7/Cacna1e/Ropn1l	21
GO:0045722	positive regulation of gluconeogenesis	6/867	24/17385	0.0009	0.968	0.968	Ppara/Nnmt/Ppp4r3a/Hif1a/Ptpn2/Sirt1	6
GO:0120316	sperm flagellum assembly	8/867	43/17385	0.001	0.968	0.968	Mns1/Klc3/Vdac3/Iqcg/Ttll1/Cep131/Cfap65/Drc7	8
GO:1901018	positive regulation of potassium ion transmembrane transporter activity	6/867	26/17385	0.001	0.968	0.968	Kcnk3/Atp1b3/Kcnc1/Abcc8/Amigo1/Atp1b1	6
GO:0001539	cilium or flagellum-dependent cell motility	17/867	155/17385	0.002	0.968	0.968	Gas2l2/Mkks/Dnai3/Mns1/Klc3/Vdac3/Tekt1/Drc1/Iqcg/Ttll1/Cep131/Cfap45/Dnah6/Cfap65/Drc7/Cacna1e/Ropn1l	17
GO:0060285	cilium-dependent cell motility	17/867	155/17385	0.002	0.968	0.968	Gas2l2/Mkks/Dnai3/Mns1/Klc3/Vdac3/Tekt1/Drc1/Iqcg/Ttll1/Cep131/Cfap45/Dnah6/Cfap65/Drc7/Cacna1e/Ropn1l	17
GO:0060271	cilium assembly	31/867	367/17385	0.003	0.968	0.968	Saxo1/Mapk15/Cep41/Mkks/Arf4/Tbc1d30/Odad4/Dnai3/Mns1/Cep162/Dnai2/Rsph1/Klc3/Clcn4/Vdac3/Tekt1/Ccdc57/Drc1/Iqcg/Ttll1/Tmem231/2700049A03Rik/Cep131/Cenpj/Pcdh15/Cfap65/Atxn10/Atg5/Drc7/Tbc1d31/Ccdc42	31
GO:0010907	positive regulation of glucose metabolic process	8/867	50/17385	0.003	0.968	0.968	Gck/Ppara/Nnmt/Ppp4r3a/Hif1a/Ptpn2/Hmgb1/Sirt1	8
GO:0060294	cilium movement involved in cell motility	16/867	149/17385	0.0031	0.968	0.968	Gas2l2/Mkks/Dnai3/Mns1/Klc3/Vdac3/Tekt1/Iqcg/Ttll1/Cep131/Cfap45/Dnah6/Cfap65/Drc7/Cacna1e/Ropn1l	16
GO:0007288	sperm axoneme assembly	6/867	30/17385	0.003	0.968	0.968	Mns1/Iqcg/Ttll1/Cep131/Cfap65/Drc7	6
Top 10 hits associated with molecular functions								
GO:0004812	aminoacyl-tRNA ligase activity	7/872	41/17304	0.004	0.944	0.944	Iars1/Fars2/Gars/Rars2/Rars1/Tars2/Cars1	7
GO:0016875	ligase activity, forming carbon–oxygen bonds	7/872	41/17304	0.004	0.944	0.944	Iars1/Fars2/Gars/Rars2/Rars1/Tars2/Cars1	7
GO:0004175	endopeptidase activity	25/872	296/17304	0.008	0.944	0.944	Eef1ece2/Tmprss7/Ecel1/Bace2/Ndel1/Mst1/Prss30/Mcpt4/Adam12/Mmp12/Pappa2/Casp2/Senp1/F3/C1s1/Prss57/Adam11/Fap/Adam1b/Capn2/Prss52/Casp9/Sec11a/Cflar/Tmprss11a	25
GO:0070034	telomerase RNA binding	4/872	17/17304	0.009	0.944	0.944	Snrpd3/Hnrnpc/Smg5/Dhx36	4
GO:0016504	peptidase activator activity	7/872	51/17304	0.013	0.944	0.944	Nkx3-1/Psme2/Pcolce/Aph1a/Fbln1/Cflar/Pcolce2	7
GO:0031418	L-ascorbic acid binding	4/872	19/17304	0.014	0.944	0.944	Ogfod2/Dbh/Ogfod3/Egln2	4
GO:0051539	4 iron, 4 sulfur cluster binding	6/872	40/17304	0.014	0.944	0.944	Mutyh/Cdk5rap1/Isca2/Nubpl/Exo5/Tyw1	6
GO:0004252	serine-type endopeptidase activity	11/872	103/17304	0.015	0.944	0.944	Tmprss7/Mst1/Prss30/Mcpt4/F3/C1s1/Prss57/Fap/Prss52/Sec11a/Tmprss11a	11
GO:0019842	vitamin binding	13/872	131/17304	0.015	0.944	0.944	Rbp2/Ogfod2/Aadat/Rbp3/Kyat3/Dbh/Ogfod3/Abca4/Folr2/Uros/Got1/Tcn2/Egln2	13
GO:0008331	high voltage-gated calcium channel activity	3/872	11/17304	0.016	0.944	0.944	Cacnb3/Cacna1s/Cacna1e	3

### 3.3 Differential gene accessibility mediated by H3K4me3 enrichment dependent on 5-HTT deficiency- and interindividual variability in social behaviour following prenatal stress

To investigate a possible involvement of H3K4me3 levels in explaining the distinct gene expression profiles, an H3K4me3 enrichment-based gene accessibility analysis was performed for the 1,484 nominal DEGs. With this analysis, we identified 19 nominally significantly enriched fragments (Table 3), of which none survived correction for multiple testing. Notably, Kif14, one of the 23 significant DEGs, displayed lower levels of H3K4me3 in socially unaffected PS *5-Htt+/+* mice.

**Table 3:** Differentially enriched fragments for H3K4me3.

Genes	logFC	logCPM	LR	PValue	FDR	Group	geneName	geneSymbol
15,107	0.51	9.78	10.16	0.001	0.8466	*5-Htt+/+* SA / *5-Htt+/+* C vs. *5-Htt+/+* SU / *5-Htt+/+* C	ENSMUSG00000020156	Pwwp3a
15,107	−0.46	9.78	8.22	0.004	0.9999	*5-Htt+/+* SU vs. *5-Htt*+/+ C	ENSMUSG00000020156	Pwwp3a
3,681	0.60	9.14	6.92	0.009	0.9999	*5-Htt+/−* SA vs. *5-Htt+/−* C	ENSMUSG00000027376	Prom2
11,399	−0.83	8.64	6.11	0.013	0.9999	*5-Htt+/+* SU vs. *5-Htt+/+* C	ENSMUSG00000035354	Uvrag
15,107	0.53	9.78	5.37	0.02	1.0000	*5-Htt+/+* SA / *5-Htt+/+* C vs. *5-Htt+/+* SU / *5-Htt+/+* C vs. *5-Htt+/−* SA / *5-Htt+/−* C vs. *5-Htt+/−* SU / *5-Htt+/−* C	ENSMUSG00000020156	Pwwp3a
15,034	0.36	9.99	5.37	0.021	0.9997	*5-Htt+/+* SA / *5-Htt+/+* C vs. *5-Htt+/+* SU / *5-Htt+/+* C	ENSMUSG00000009070	Rsph14
11,399	0.77	8.64	5.32	0.021	0.9997	*5-Htt+/+* SA / *5-Htt+/+* C vs. *5-Htt+/+* SU / *5-Htt+/+* C	ENSMUSG00000035354	Uvrag
1,330	−0.35	9.90	5.16	0.023	0.9999	*5-Htt+/+* SU vs. *5-Htt+/+* C	ENSMUSG00000041498	Kif14
20,504	0.34	10.00	5.02	0.025	0.9997	*5-Htt+/+* SA / *5-Htt+/+* C vs. *5-Htt+/+* SU / *5-Htt+/+* C	ENSMUSG00000096629	Gm3383
23,262	0.42	9.37	4.69	0.03	0.9997	*5-Htt+/+* SA / *5-Htt+/+* C vs. *5-Htt+/+* SU / *5-Htt+/+* C	ENSMUSG00000099958	1700010B13Rik
30,698	−0.51	9.14	4.46	0.035	0.9999	*5-Htt+/−* SU vs. *5-Htt+/−* C	ENSMUSG00000048120	Entpd1
15,034	−0.32	9.99	4.34	0.037	0.9999	*5-Htt+/+* SU vs. *5-Htt+/+* C	ENSMUSG00000009070	Rsph14
11,383	−0.33	10.26	4.32	0.038	0.9999	*5-Htt+/+* SU vs. *5-Htt+/+* C	ENSMUSG00000035354	Uvrag
5,503	−0.60	8.81	4.30	0.038	0.9999	*5-Htt+/+* SU vs. *5-Htt+/+* C	ENSMUSG00000105891	A230001M10Rik
30,698	0.49	9.14	4.25	0.039	0.9999	*5-Htt+/−* SA / *5-Htt+/−* C vs. *5-Htt+/−* SU / *5-Htt+/−* C	ENSMUSG00000048120	Entpd1
15,034	0.32	9.99	4.09	0.043	0.9999	*5-Htt+/−* SA / *5-Htt+/−* C vs. *5-Htt+/−* SU / *5-Htt+/−* C	ENSMUSG00000009070	Rsph14
19,251	−0.33	10.38	3.94	0.047	0.9999	*5-Htt+/+* SU vs. *5-Htt+/+* C	ENSMUSG00000097565	Gm26965

## 4 Discussion

The aim of the current study was to investigate the interaction between genetic variation in *5-Htt* expression and prenatal maternal stress in the context of interindividual behavioural variability. Behavioural screening of several behavioural parameters indicative of affective behaviour and genome-wide profiling of mRNA expression and H3K4me3 enrichment revealed, as expected, variations in adult affective behaviour of mice exposed to PS associated with a distinct, partly 5-HTT-dependent, behavioural and transcriptional profile.

Generally, PS in rodents has been associated with a multitude of behavioural changes, such as increased anxiety (Schulz et al., 2011; Zohar and Weinstock, 2011; Hiroi et al., 2016), altered social behaviour (Schulz et al., 2011; Miyagawa et al., 2011) and increased passive coping during acute swim stress exposure as in the PST (Alonso et al., 1991; Hiroi et al., 2016). In previous work within our own group, the same PS paradigm as used in the current study, was associated with increased passive coping in the PST, particularly pronounced in *5-Htt*+/− female offspring and, overall, with decreased distance covered in the elevated zero maze (van den Hove et al., 2011). A closer look at those behavioural effects of PS showed that not all offspring responded to PS exposure to the same degree, with, e.g., a subset of PS offspring showing behavioural performance comparable to control offspring, suggesting interindividual variability among PS offspring (Jakob et al., 2014).

In the present study, we aimed to investigate this interindividual variability more extensively, making use of a larger group of PS offspring. As expected, we observed interindividual variability in response to PS in some of the behavioural tasks. More specifically, while a substantial proportion of PS offspring showed decreased sociability, an almost equal amount of animals showed levels of sociability indistinguishable from control offspring. Interestingly, in the EPM, 5-Htt+/+ offspring that were affected with regard to social behaviour (i.e., the SA group) at the same time showed signs of reduced anxiety when compared to control offspring, whereas 5-Htt+/+ offspring unaffected with regard to social behaviour (i.e., the SU group) displayed levels of anxiety comparable to control offspring. In 5-Htt+/− offspring, however, this relation was inverted, with PS offspring affected with regard to social behaviour showing no change in anxiety when compared to control mice, and PS offspring with intact social behaviour displaying reduced anxiety. These remarkable results suggest behavioural adaptation and coping styles in response to PS to be both context- (i.e., test-) and genotype-dependent.

The observed differential test-specific and 5-HTT-dependent PS effects suggest that 5-HT-mediated developmental programming is highly specific and context-dependent. As such, 5-HTT deficiency seems to affect how the brain translates signals associated with environmental variation, in this case prenatal maternal stress exposure, into molecular changes within specific brain regions (Homberg and van den Hove, 2012). In support of this notion, following low maternal care, levels of brain-derived neurotrophic factor (BDNF) mRNA were exclusively increased in the hippocampus of 5-HTT-deficient offspring, but not in wildtype offspring, and correlated with the time spent in the centre of the open field, while, of note, *Bdnf* expression in the amygdala was not affected by either variations in levels of maternal care or *5-Htt* genotype (Carola et al., 2008). A key player that may explain this region- and context-specific, 5-HT-dependent developmental programming of plasticity-related gene expression is epigenetic regulation (Homberg and van den Hove, 2012). For example, the expression of the glucocorticoid receptor, which is known to play a key role in regulating stress-sensitivity, has been shown to be increased by maternal licking and grooming through 5-HT-mediated epigenetic changes (Weaver et al., 2007; Hellstrom et al., 2012). This suggests that, next to direct effects, genetic variation linked to the 5-HT system may be able to potentiate or buffer individual experiences via epigenetic processes (Homberg and van den Hove, 2012; Clayton et al., 2020).

However, it has to be mentioned that, although we expected to observe interindividual variability in response to PS in particular with regard to passive coping as observed in the PST based on our previous work (Jakob et al., 2014), we did not observe we did not observe any statistically significant variability in passive coping. This apparent discrepancy may be explained by a different experimental design. First, in the previous study, *5-Htt+/−* dams (next to 5-Htt+/− males) were being used for breeding, while in the present study all pregnant dams were wildtype (C57BL6/J) mice (mated with *5-Htt+/−* males). Maternal genotype, and associated maternal 5-HT availability, can provide a different prenatal environment but also affect maternal behaviour after birth and thus provide a different postnatal (maternal) environment. *5-Htt+/−* animals are known for displaying an altered stress response (Murphy and Lesch, 2008) and therefore likely respond differently to stress during pregnancy (Meaney, 2001), concomitant with different programming effects in utero. In line with this, work by Jones et al. (2010) showed that offspring of both *5-Htt+/−* and *5-Htt+/+* dams that were stressed during pregnancy showed signs of general increased active coping in the EPM, while decreased social preference was dependent on the maternal genotype, independent from offspring genotypic differences, with the most pronounced effects of PS being observed in offspring of PS *5-Htt+/−* dams, an effect that has been postulated to be linked to differences in maternal care. This indicates the importance of maternal behaviour, a parameter that should be screened for in further early life stress studies. Second, the behavioural test battery we employed in the current study was also quite different from the experimental setup in the previous study, setting a different context for animals of the two different study cohorts. Due to parental genotype and associated 5-HT availability, behavioural differences may arise in mice prenatally stressed and with the same genotype.

Of note, the behavioural tests employed in the current study cover a range of behaviours relevant to the spectrum of affective disorders. Using the EPM and PST, behavioural coping in adverse environments was investigated. While in the EPM animals are exposed to a choice between exploring elevated open arms that expose them to potential danger and closed arms that reflect (relative) safety (Harro, 2018), the PST does not leave the animals with a choice to avoid aversion (Commons et al., 2017). As stress coping strategies represent an indicator of an individual animal’s capacity to buffer adverse experiences, they are a likely reporter of human psychopathology and resilience (Zimmer-Gembeck and Skinner, 2016). The 3-CST on the other hand is used to examine the preference of social stimuli such as odour and visual and auditory contact with a conspecific, by giving the choice to enter a room containing or devoid of a social target (Moy et al., 2004; Nadler et al., 2004). As such, performance in the 3-CST is likely to reflect the innate drive of animals to interact socially. In humans, increased social drive is often associated with an increased coping capacity (Zimmer-Gembeck and Skinner, 2016). Taken together, although speculative, the present results suggest that altered sociability, which is potentially indicative of variations in coping (styles), is independent of coping strategies in response to acute external stressors and that the coping strategy to acute external stressors is affected by the *5-Htt* genotype as seen in the EPM.

Altogether, the current behavioural data show only minor effects of PS and 5-Htt genotype, which is not unexpected, as PS in rodents has been associated with a multitude of behavioural changes ranging from increased anxiety (Schulz et al., 2011; Zohar and Weinstock, 2011; Hiroi et al., 2016) to increased passive coping during acute swim stress exposure (Alonso et al., 1991; Hiroi et al., 2016). While all these studies were showing one or two behavioural profiles to be affected by PS, it is rarely seen that PS affects a full spectrum of behavioural traits. The exact nature and degree of these changes depends on, e.g., the species, strain and sex of rodents used, the behavioural screening conditions and the nature of the PS paradigm (Weinstock, 2008) thus the observed subtle effects of PS might be attributed to an otherwise stress-free environment. With regard to the 5-Htt genotype, *5-Htt +/−* mice have been reported to manifest no behavioural differences when compared to wildtype animals in a variety of tests ranging from EPM to PST, even when exposed to early life stress (Carroll et al., 2007). It is thus possible that the PS paradigm used in this setup together with the maternal genotype and the resulting postnatal environment was not sufficient to evoke strong effects. These observations further support the notion of variable effects of PS being dependent not only on one or two distinct factors like duration or nature of the stressor, but suggests that a complex network of (interdependent) effectors regulate early developmental programming of brain functions with consequences for later responsivity to (e.g., social and/or stressful) stimuli and associated behaviours.

Given the distinct behavioural profile of SA and SU offspring, we were interested in determining the distinct gene expression profiles in the hippocampus of each of these groups compared to the control offspring, as well as in examining the effects of 5-HTT deficiency on each of these profiles. While in the H3K4me3 enrichment-based gene accessibility analysis none of the identified nominally significantly enriched fragments survived correction for multiple testing, 23 significant DEGs were found. Interestingly, only five of the 23 identified DEGs constitute protein-coding genes, all of which represent, to the best of our knowledge, novel candidates for serotonin-dependent stress regulation and differential susceptibility. Kinesin family member 14 (KIF14) is a N-kinesin with the motor domain at the N-terminus (Miki et al., 2001) that has been linked to cytokinesis (Carleton et al., 2006). Interference with KIF14 function results in multinucleated cells or acute apoptosis. A mouse mutant with a spontaneous *Kif14* mutation showed disorganised cytoarchitecture within the hippocampus, cerebral and cerebellar cortices as well as in the olfactory bulb (Fujikura et al., 2013; Yunus et al., 2014). Further phenotypic features of the so-called laggered mutant mouse is hypomyelination and reduced brain volume. In humans, *KIF14* variants have been associated with microcephaly (Makrythanasis et al., 2018; Moawia et al., 2017). Both human and mouse observations support the suggested role in completion of cytokinesis (Moawia et al., 2017; Fujikura et al., 2013; Gruneberg et al., 2006). Altered KIF14 function might thus affect hippocampal cytoarchitecture and, as a consequence, information processing in hippocampus-related tasks and associated behaviours. Notably, *Kif14* expression was increased both in *5-Htt+/+* and *5-Htt+/−* offspring displaying intact social behaviour, but not in offspring socially affected by PS (i.e., the SA group), when compared to their respective control groups. Although purely speculative, this may indicate that KIF14 activity contributes to an altered behavioural response to PS exposure. Interestingly, in addition to an increase in mRNA expression, we observed a decrease in *Kif14* H3K4me3 in *5-Htt+/+,* but not *5-Htt+/−* SU offspring. The negative correlation between *Kif14* H3K4me3 enrichment and mRNA expression suggests a multi-levelled regulation, where, for example, H3K9me2 and H3K9ac together with H3K4me3 can act as a switch between gene activation and repression (Sharma, 2012; Gates et al., 2017). Hypothetical, histone serotonylation, which has been shown to enhance H3K4me3 binding to transcription factor II D (TFIID) (Farrelly et al., 2019), might be the cause of altered *Kif14* enrichment in *5-Htt+/+* but not in *5-Htt+/−* SU offspring owing to the changes in serotonin homeostasis in *5-Htt+/−* animals.

Mitogen-activated protein kinase 15 (MAPK15), also referred to as extracellular signal-regulated kinase 7/8 (ERK7/8), is an atypical MAPK (Abe et al., 1999). *In vitro*, MAPK15 has been shown to phosphorylate myelin basic protein (Abe et al., 2002). More recently, in *C. elegans*, it was reported to regulate the dopamine transporter (Bermingham et al., 2017). Moreover, it seems to regulate oestrogen receptor alpha availability and to inhibit its transcription factor activity *in vitro* (Henrich et al., 2003; Rossi et al., 2011). The oestrogen receptor alpha is involved in sex-specific modulation of interneurons in the hippocampus (Tabatadze et al., 2015) and has been shown to mediate stress-related regulation of gamma-aminobutyric acid (GABA) release from endocannabinoid receptor 1 containing interneurons in a sex-specific manner (Ferraro et al., 2020). In our study, we found *Mapk15* to display increased expression in PS *5-Htt*+/+ offspring compared to control *5-Htt*+/+ offspring, independent of variations in social behaviour. Notably, in *5-Htt*+/− PS offspring, *Mapk15* expression was decreased in SA offspring, while being increased in SU offspring, when compared to control offspring. With its potential effect on GABA signalling, this complex expression profile of *Mapk15* hints towards a GABA-mediated difference in hippocampal homeostasis between *5-Htt*+/+ and *5-Htt*+/− female offspring. Clearly, this notion warrants further research.

Otopetrin 2 (Otop2) is a paralogue of Otop1 and shows 34% amino acid identity with Otop1 (Hurle et al., 2003). Together with Otop3, Otop2 and 1 comprise the otopetrin gene family, which shows homology to the *C. elegans* and D. melanoganster DUF270 genes (Hurle et al., 2003). Otop1 was discovered in 2003 in the context of the classical mouse mutant tilted, which is associated with impaired balance (Hurle et al., 2003). Secondary structure analysis suggested a function as ion channel or transporter for Otop1 (Hurle et al., 2003). Diverse physiological studies across various taxa suggest an involvement of Otop1 in calcium (Ca2+) secretion (Hughes et al., 2008). Recent work by Tu et al. (2018) showed that otopetrin genes encode highly selective ion channels that are specific for proton transport. It was furthermore reported that channel activity is pH-dependent with opposing reactions to extracellular and intracellular low pH (Chen et al., 2019). Channels sensitive to changes in pH have been associated with various functions in the brain (Askwith et al., 2004; Sherwood et al., 2011; Uchitel et al., 2019), among which the regulation of fear- and anxiety-related behaviours. In both *5-Htt+/+* SA and SU a slightly increased Otop2 expression was found compared to the control group. In *5-Htt*+/− mice, Otop2 expression was increased in SA and decreased in SU compared to the control group. Like with *Mapk15*, this profile of an ion channel supports 5-HT-dependent differences in hippocampal homeostasis and reactivity.

Chemokine (C-C motif) ligand 21 (Ccl21) is a cytokine in the family of the cc chemokines. Instead of the common four cysteine residues of chemokines, CCL21 has six cysteine residues that is why it is also called 6CKINE (Hedrick and Zlotnik, 1997). The murine CCL21 is subdivided in three different groups, i.e., CCL21A, CCL21B and CCL21C. Whereas CCL21A consists of six cysteines and a serine residue and CCL21B and CCL21C have a leucine residue, CCL21A might be expressed in secondary lymphoid organs and CCL21B might be more expressed in non-lymphoid tissue (Chen et al., 2002). CCL21 is known to bind to CCR7 (Yoshida et al., 1998), and has been found to play a role in the immunoreaction activating the CXCR3 in the brain, where damaged neurons express CCL21, activating the CXCR3, which in turn triggers chemotaxis in murine microglia (Biber et al., 2001; Rappert et al., 2002). Van Weering et al. (2010) found CCL21 to be associated with induced responses of primary mouse astrocytes in an *in vitro* model and suggested that CCL21 plays a role in communication between neurons and surrounding glia cells under pathological conditions as both microglia and astrocytes have the capacity to respond to CCL21.

Eef1akmt4-endothelin converting enzyme 2 readthrough (Eef1ece2) has been found to encode a readthrough transcription between the two genes endothelin converting enzyme 2 (Ece2) and elongation factor alpha lysine specific methyltransferase 4 (Eef1akmt4). Ece2 is believed to play a role in neurodevelopment, where it is believed to promote neuronal differentiation (Buchsbaum et al., 2020). Eef1akmt4 is responsible for the methylation of lysine residue K36 in the eukaryotic translation elongation factor 1 alpha (eEF1A) (Jakobsson et al., 2017). The Eef1ece2 gene has not been associated with any function, process or component yet. However, the fusion protein produced by the transcript shares sequence identity with both individual gene products (RefSeq July 2017).

Next, to the five protein coding genes, we found 18 DEGs, categorised as ‘pseudogenes’, ‘processed pseudogenes’ and ‘predicted genes’. Pseudogenes have gained more and more recognition throughout recent years, in particular with the progression of whole-genome analyses. First understood as a “relic of evolution,” a pseudogene is nowadays virtually any sequence with high resemblance to protein-coding genes (Cheetham et al., 2020). As we can see in our results, over 80% of our DEGs reflect unknown or not yet verified genes. While the specific functions of each identified pseudogene are unknown, pseudogenes in general exert a wide variety of functions. For example, with regard to their evolutionary origin and structure, pseudogenes are suggested to take on regulatory roles (Singh et al., 2020; Kovalenko and Patrushev, 2018). Roberts and Morris postulated a potential epigenetic regulatory role for pseudogenes (Roberts and Morris, 2013). The potential impact of pseudogenes is tremendous, as they could even be targeted in the development of novel therapeutic strategies (Costa et al., 2010; Roberts and Morris, 2013) and for diagnosis (Chen et al., 2020). It is certainly imperative to focus future research on unravelling the interplay of classic genes and pseudogenes in more detail. To this end, it is of interest to make use of the vast body of data created by whole-genome hypothesis-free approaches, while targeted approaches using, e.g., CRISPR/Cas might provide more insight in the role of individual pseudogenes.

Investigating the interindividual variability on a behavioural and molecular level has proven to be more challenging than anticipated, as the observed behavioural effects were less pronounced than expected and in accordance the gene expression and epigenetic changes were small. Further limitations that need to be considered when interpreting the present findings are as follows: as the current behavioural and molecular analyses were conducted in a large sample of female mice to further investigate the behavioural variation we previously observed, the use of a single sex-design limits the generalisation of results to males. Additionally, as the estrous cycle was not monitored in this study, it cannot be excluded that hormonal alterations may have affected behaviour and molecular outcomes. Moreover, in accordance with our previous work, brain regions were dissected without taking into account existing subregions. In the hippocampus functional subdivisions are the ventral and dorsal part (Fanselow and Dong, 2010), with the ventral part being involved in emotions, stress, and affective processes (Felix-Ortiz and Tye, 2014; Trent and Menard, 2010; McEown and Treit, 2013), and the dorsal part in cognition and memory (Moser et al., 1995; Pothuizen et al., 2004). By pooling these subregions as well as the left and right hemisphere noise might have been introduced. The results thus have to be interpreted in this light. The focus on only the hippocampus is an additional limitation as brain structures such as prefrontal cortex and amydala are also involved in emotions and affect. Lastly, due to the exploratory nature of our genome-wide mRNA sequencing and H3K4me3 enrichment analysis, the identified DEGs and the nominally involved differentially enriched H3K4me3 immunoreactive fragments need to be validated by alternative methods. In addition, the analysis should be extended to other epigenetic modifications that might be related such as DNA methylation, such as acetyl-lysine 9 H3 histone (H3K9ac), given the potential of epigenetic research to extend our current understanding of the pathophysiology of mental disorders (Gaszner et al., 2022b; Mill and Petronis, 2007).

In conclusion, overall, the current study shows that exposure to PS resulted in a notable decrease in social behaviour. This decrease in social behaviour among PS offspring was more pronounced in *5-Htt*+/− mice and showed a substantial degree of interindividual variability, suggesting that part of the offspring coped differently with PS. Moreover, this differential response was distinguished by specific, *5-Htt* genotype-dependent transcriptomic and epigenetic signatures, supporting the concept of 5-HT-dependent, developmental programming. Further exploring these molecular signatures both in animal models and in humans (Ell et al., 2024), as well as potential sex differences, might shed more light on the processes mediating differential vulnerability to psychopathology and, as such, may open avenues for the development of novel treatments for stress-related disorders.

## Data Availability

The datasets presented in this study can be found in online repositories. The names of the repository/repositories and accession number(s) can be found at: https://www.ncbi.nlm.nih.gov/geo/, GSE109929.

## Footnotes

^1^ https://doi.org/10.5281/zenodo.3564938
