# Supplementary material for: Differential susceptibility to prenatal stress exposure in serotonin transporter-deficient female mice—an epigenetic exploration
Source: Front Neurosci. 2025 Sep 23;19:1633386. doi: 10.3389/fnins.2025.1633386 (PMC12500653; doi:10.3389/fnins.2025.1633386)
Supplement: Supplementary file 1 [file Data_Sheet_1.pdf]

Supplementary Figure SI11: normalized read counts

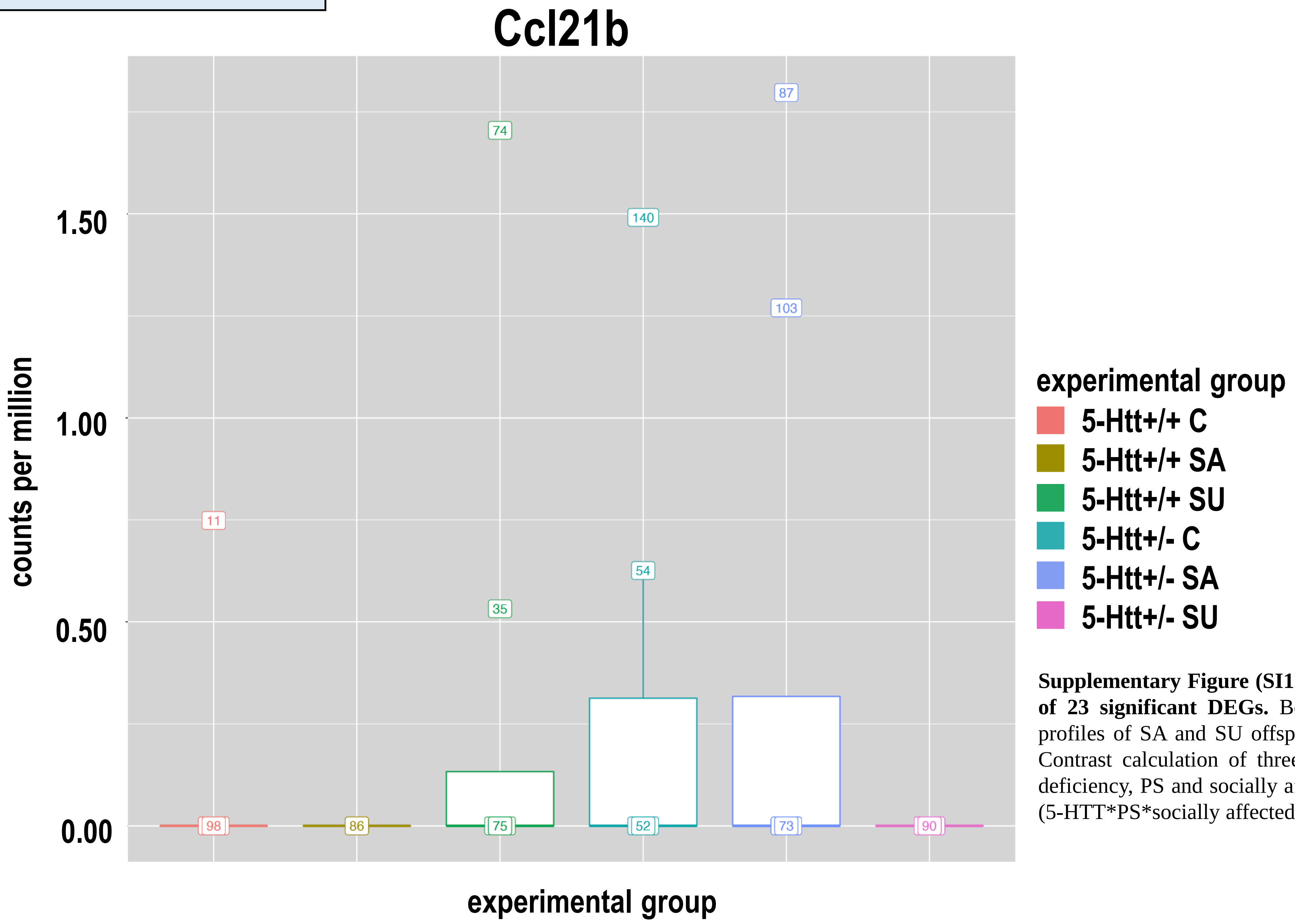

Supplementary Figure (SI11). Normalized read counts of 23 significant DEGs. Boxplots showing expression profiles of SA and SU offspring groups compared to C. Contrast calculation of three-way interaction of 5-HTT deficiency, PS and socially affected/unaffected behaviour (5-HTT*PS*socially affected/unaffected behaviour).

Supplementary Figure SI11: normalized read counts

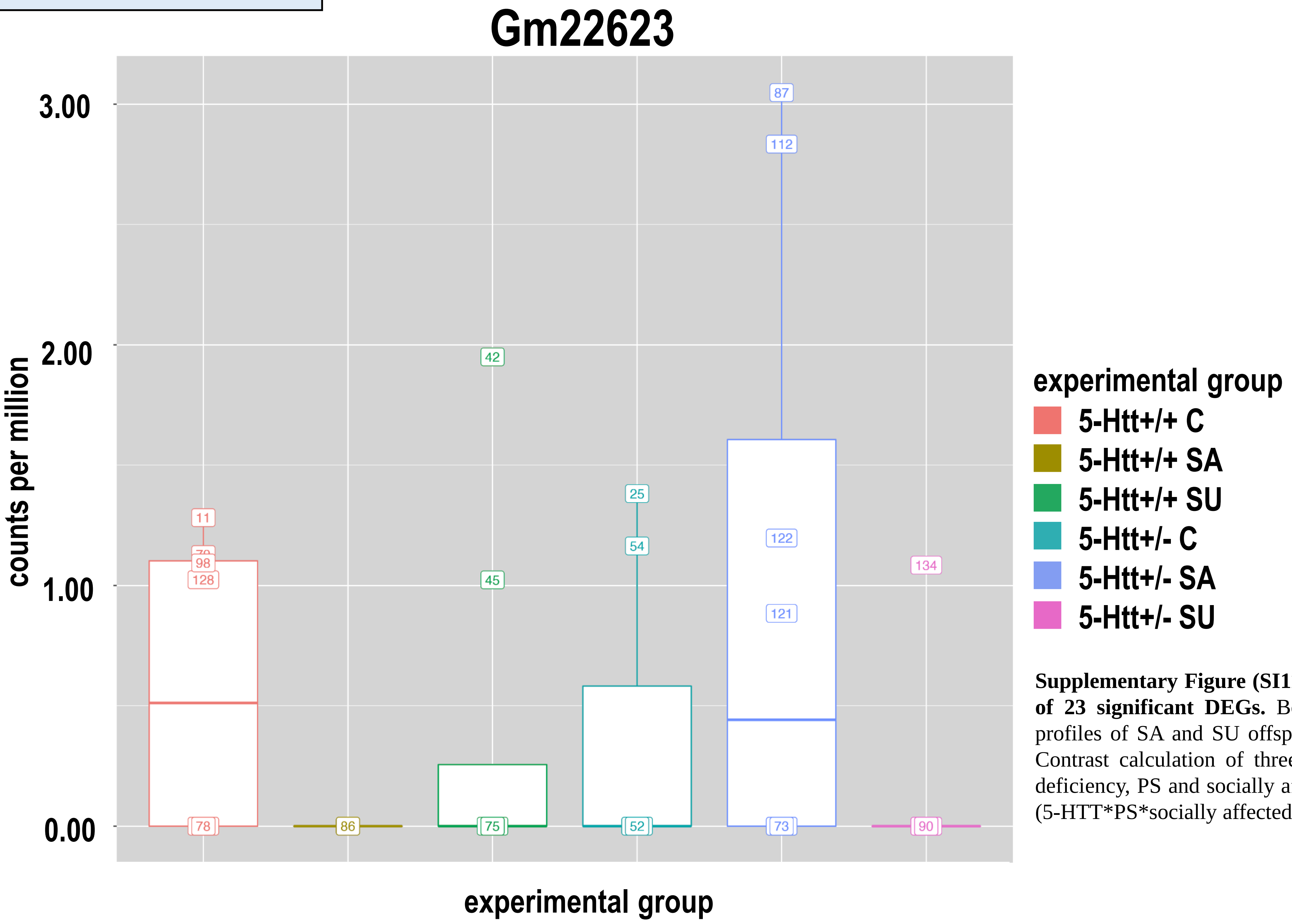

Supplementary Figure (SI11). Normalized read counts of 23 significant DEGs. Boxplots showing expression profiles of SA and SU offspring groups compared to C. Contrast calculation of three-way interaction of 5-HTT deficiency, PS and socially affected/unaffected behaviour (5-HTT*PS*socially affected/unaffected behaviour).

Supplementary Figure SI11: normalized read counts

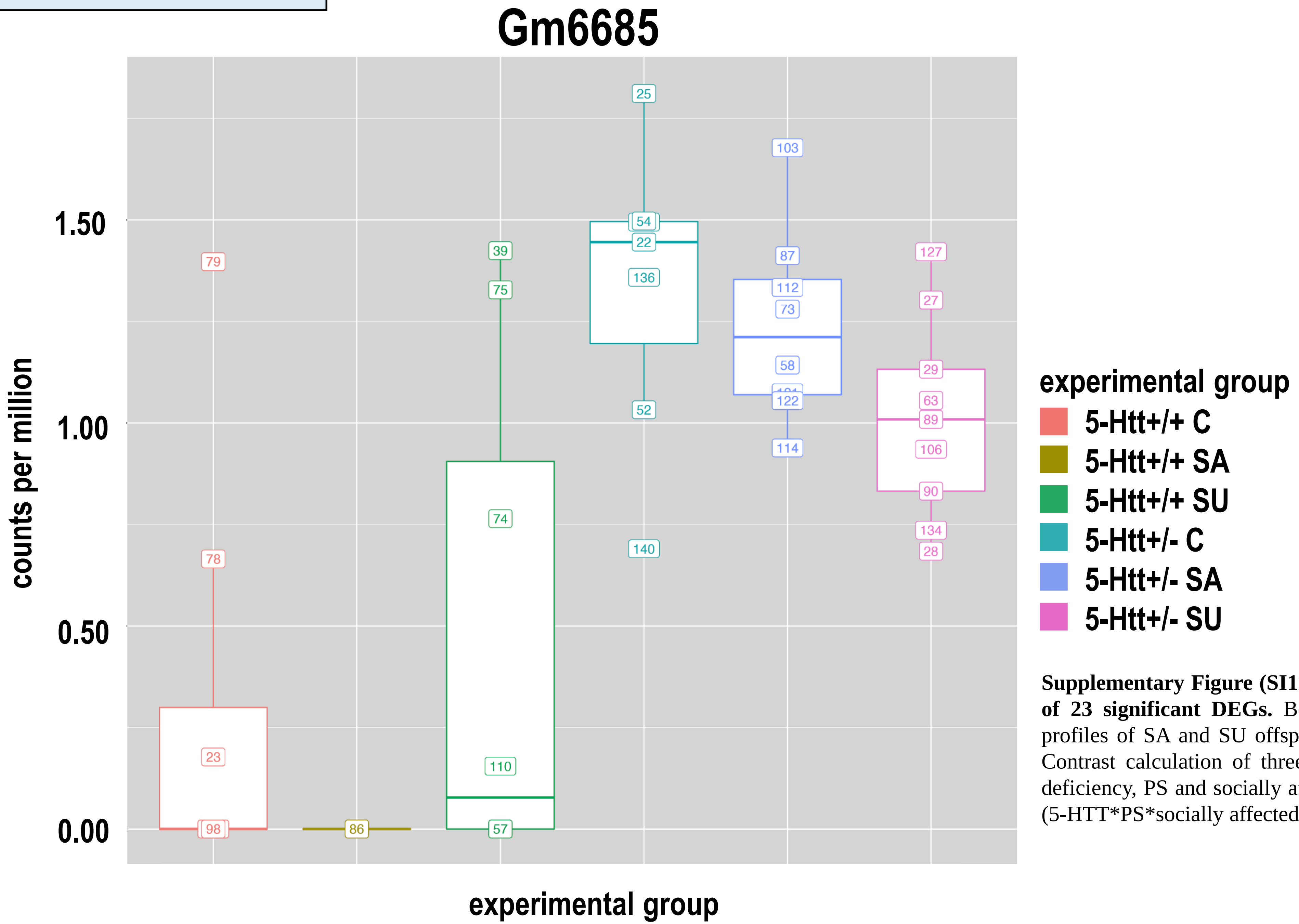

Supplementary Figure (SI11). Normalized read counts of 23 significant DEGs. Boxplots showing expression profiles of SA and SU offspring groups compared to C. Contrast calculation of three-way interaction of 5-HTT deficiency, PS and socially affected/unaffected behaviour (5-HTT*PS*socially affected/unaffected behaviour).

Supplementary Figure SI11: normalized read counts

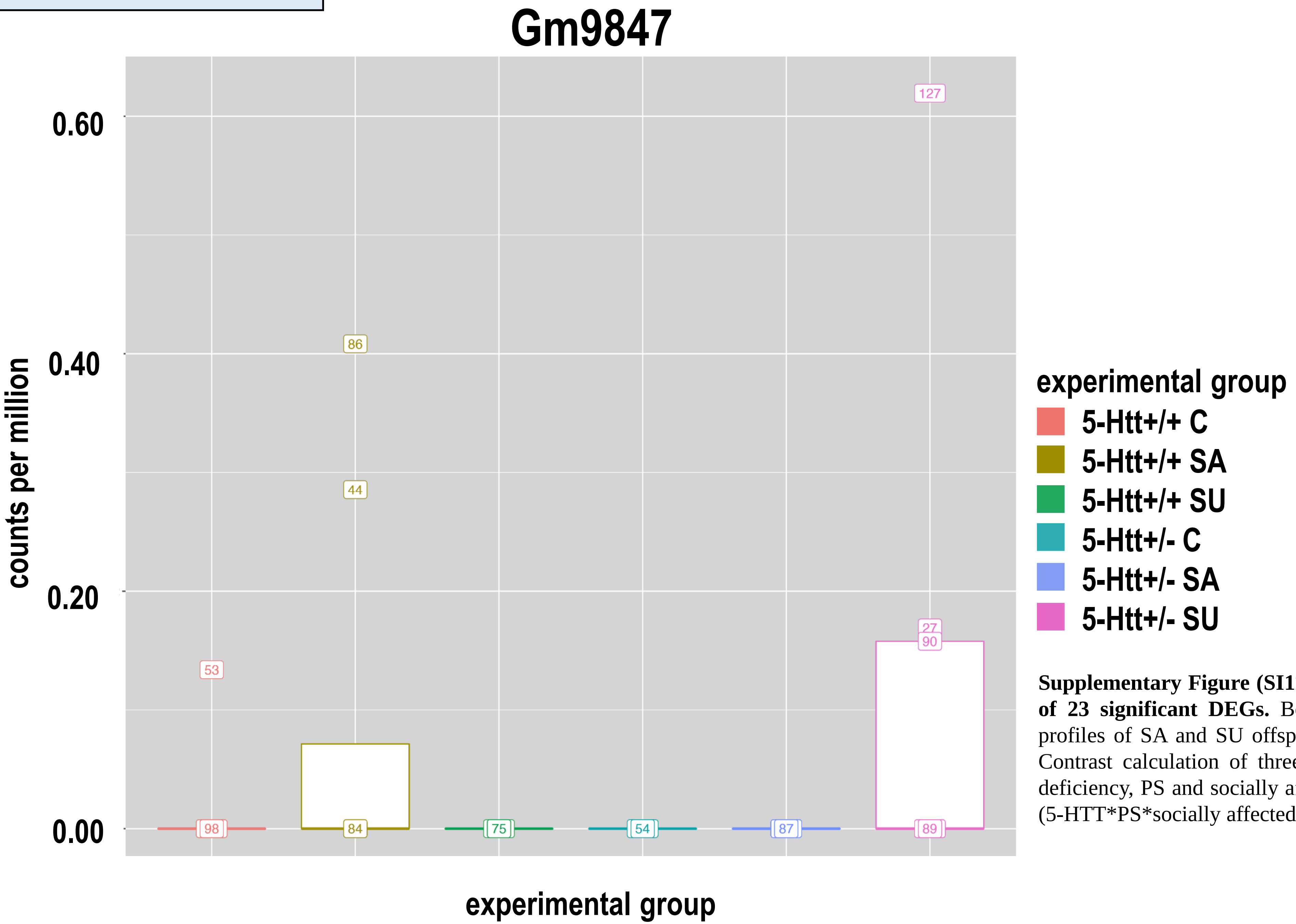

Supplementary Figure (SI11). Normalized read counts of 23 significant DEGs. Boxplots showing expression profiles of SA and SU offspring groups compared to C. Contrast calculation of three-way interaction of 5-HTT deficiency, PS and socially affected/unaffected behaviour (5-HTT*PS*socially affected/unaffected behaviour).

Supplementary Figure SI11: normalized read counts

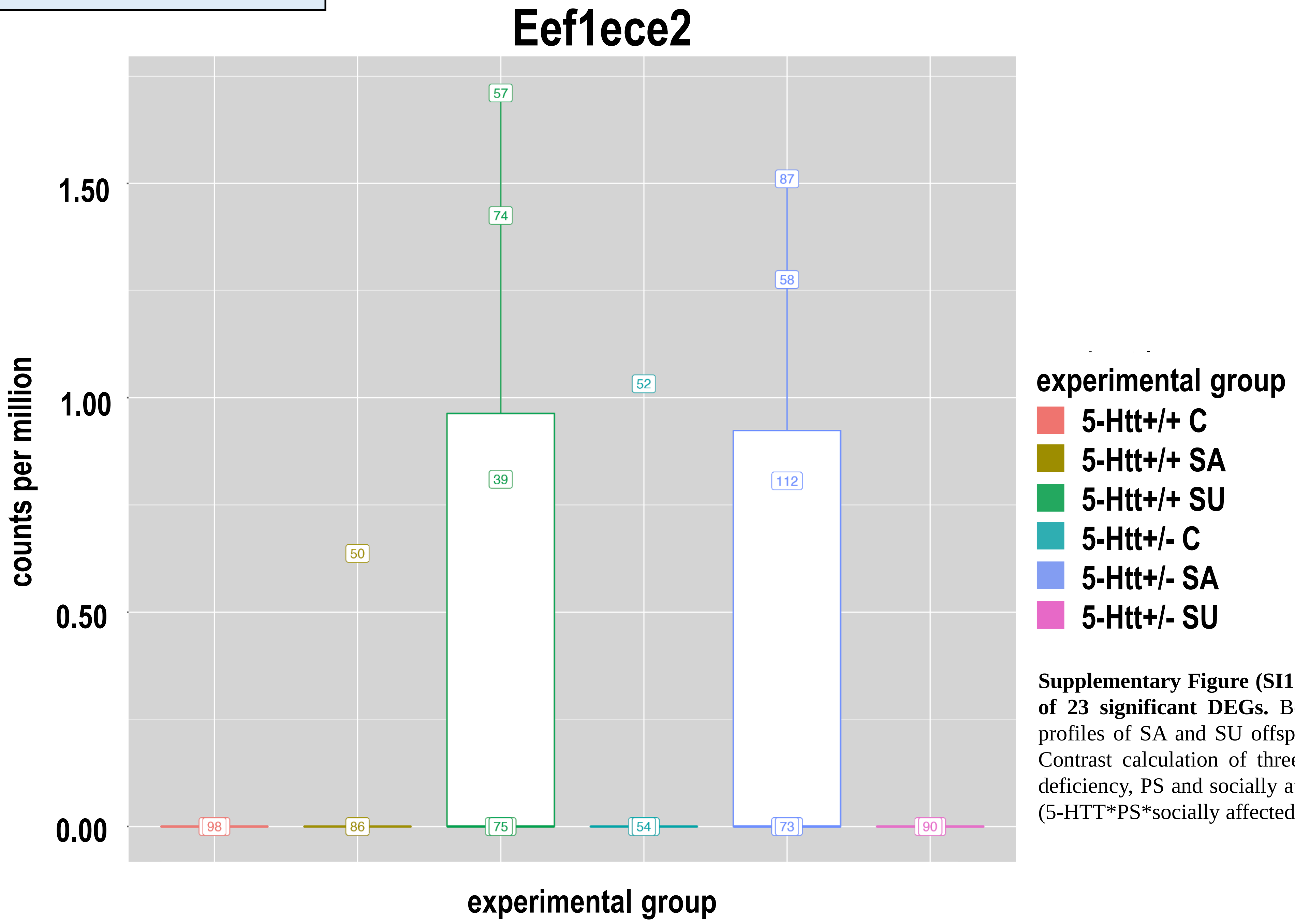

Supplementary Figure (SI11). Normalized read counts of 23 significant DEGs. Boxplots showing expression profiles of SA and SU offspring groups compared to C. Contrast calculation of three-way interaction of 5-HTT deficiency, PS and socially affected/unaffected behaviour (5-HTT*PS*socially affected/unaffected behaviour).

Supplementary Figure SI11: normalized read counts

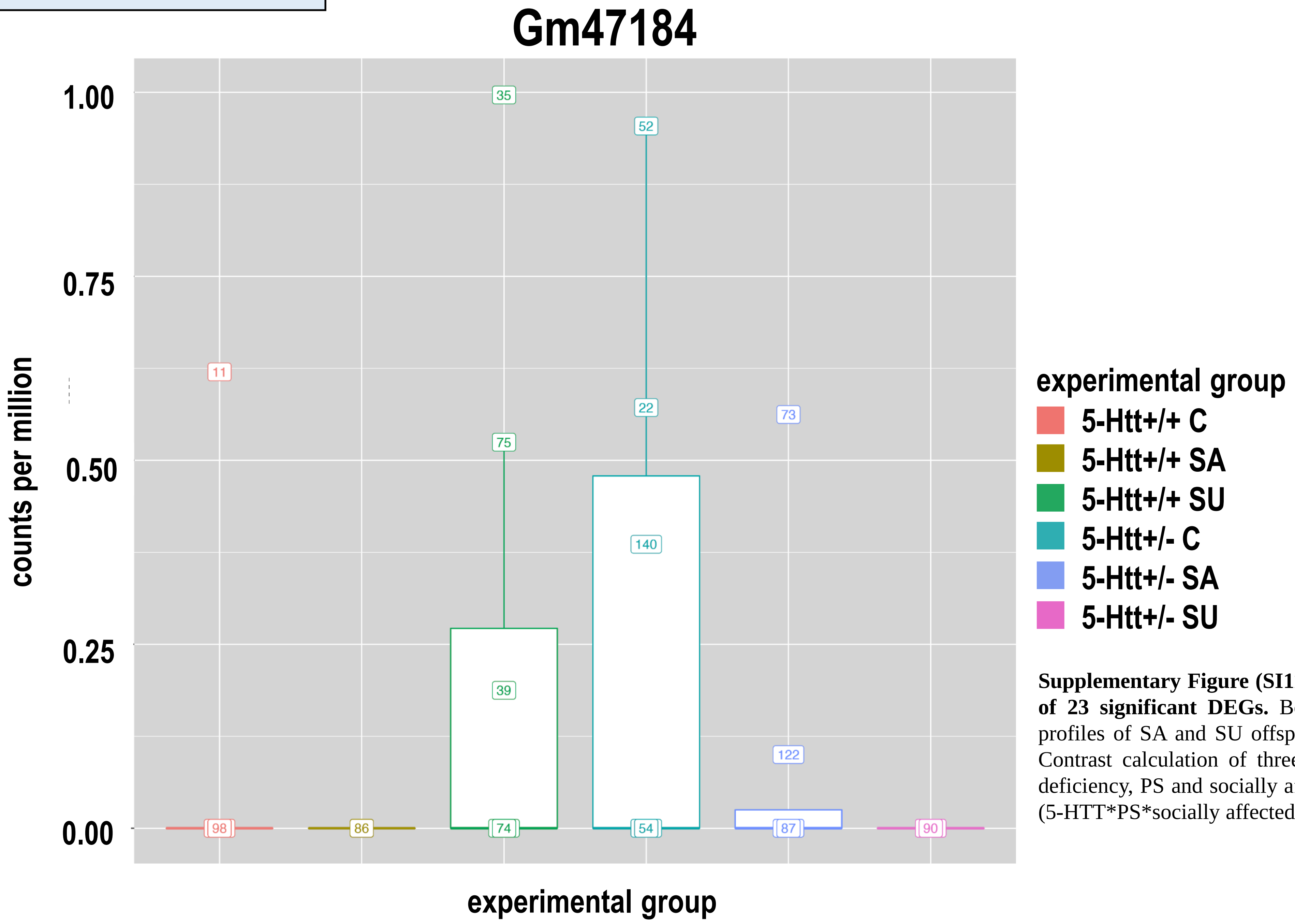

Supplementary Figure (SI11). Normalized read counts of 23 significant DEGs. Boxplots showing expression profiles of SA and SU offspring groups compared to C. Contrast calculation of three-way interaction of 5-HTT deficiency, PS and socially affected/unaffected behaviour (5-HTT*PS*socially affected/unaffected behaviour).

Supplementary Figure SI11: normalized read counts

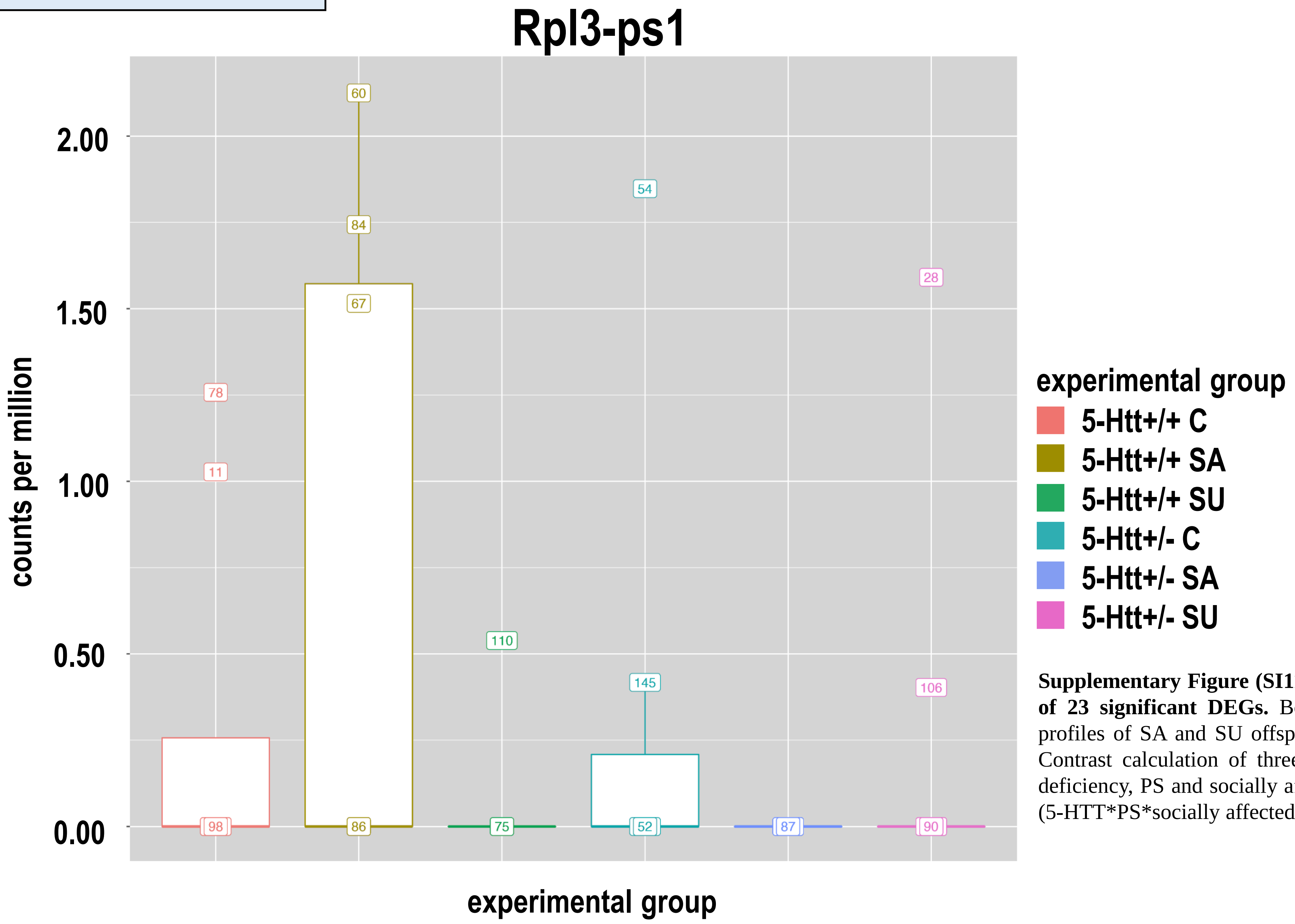

Supplementary Figure (SI11). Normalized read counts of 23 significant DEGs. Boxplots showing expression profiles of SA and SU offspring groups compared to C. Contrast calculation of three-way interaction of 5-HTT deficiency, PS and socially affected/unaffected behaviour (5-HTT*PS*socially affected/unaffected behaviour).

Supplementary Figure SI11: normalized read counts

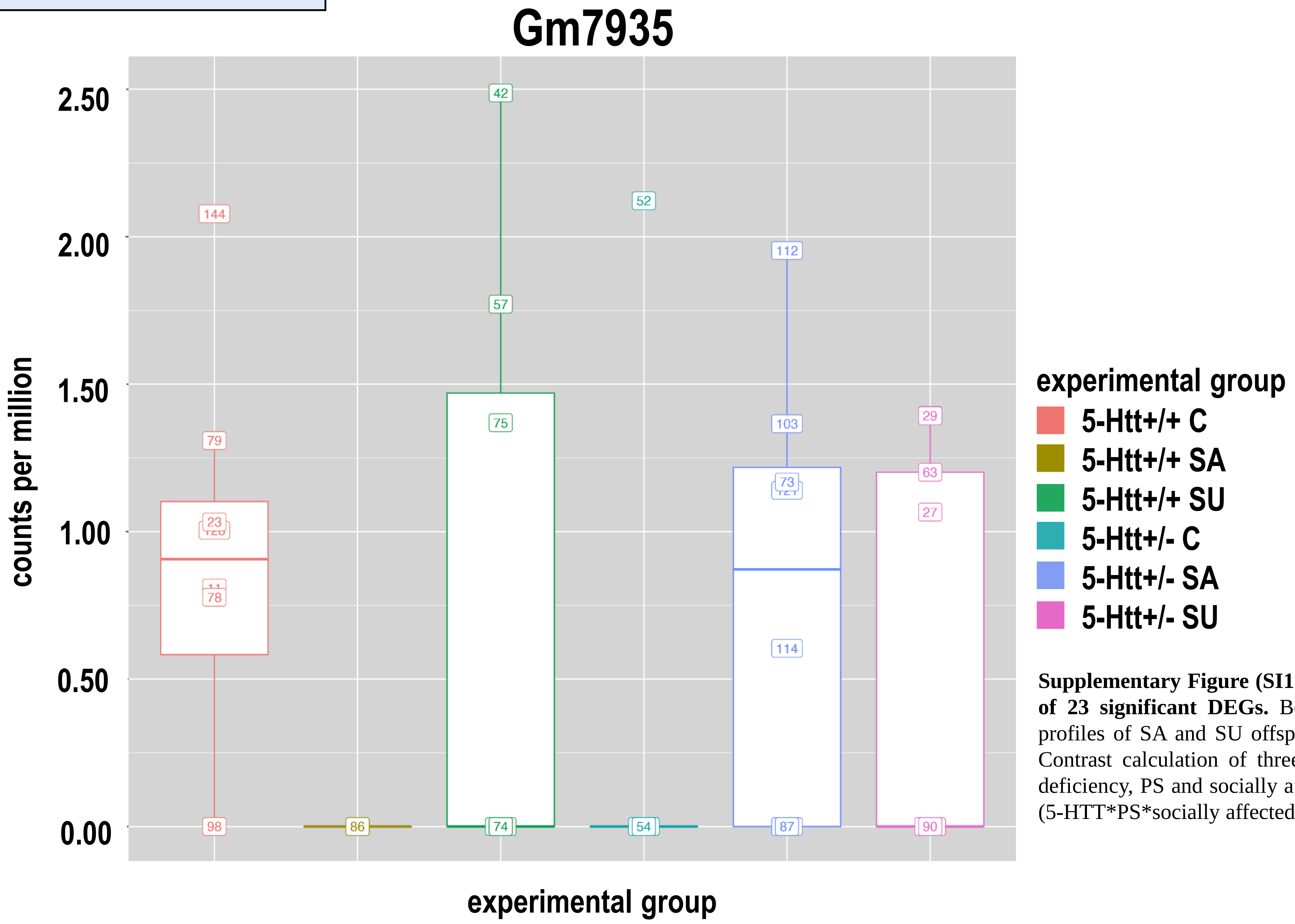

Supplementary Figure (SI11). Normalized read counts of 23 significant DEGs. Boxplots showing expression profiles of SA and SU offspring groups compared to C. Contrast calculation of three-way interaction of 5-HTT deficiency, PS and socially affected/unaffected behaviour (5-HTT*PS*socially affected/unaffected behaviour).

Supplementary Figure SI11: normalized read counts

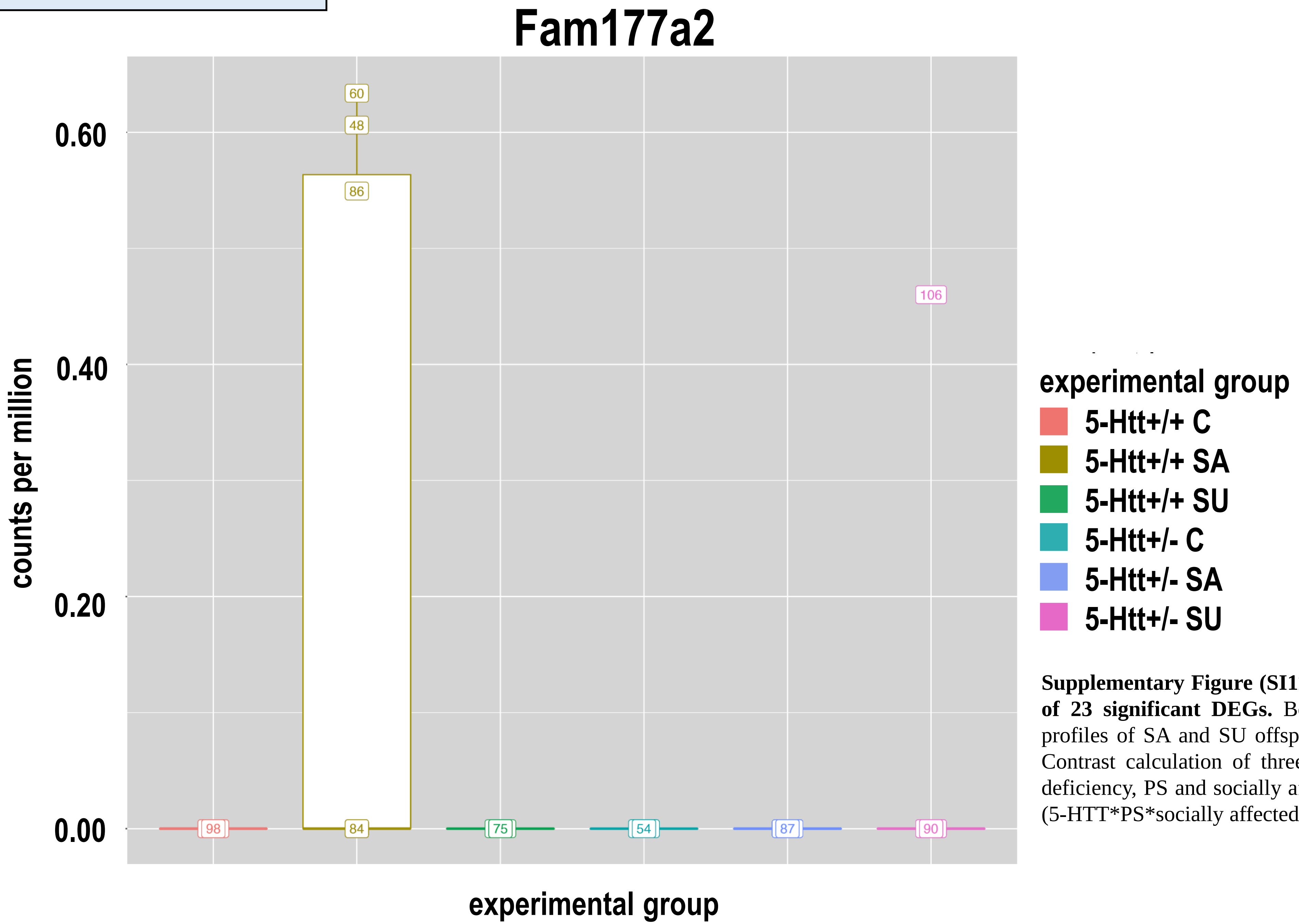

Supplementary Figure (SI11). Normalized read counts of 23 significant DEGs. Boxplots showing expression profiles of SA and SU offspring groups compared to C. Contrast calculation of three-way interaction of 5-HTT deficiency, PS and socially affected/unaffected behaviour (5-HTT*PS*socially affected/unaffected behaviour).

Supplementary Figure SI11: normalized read counts

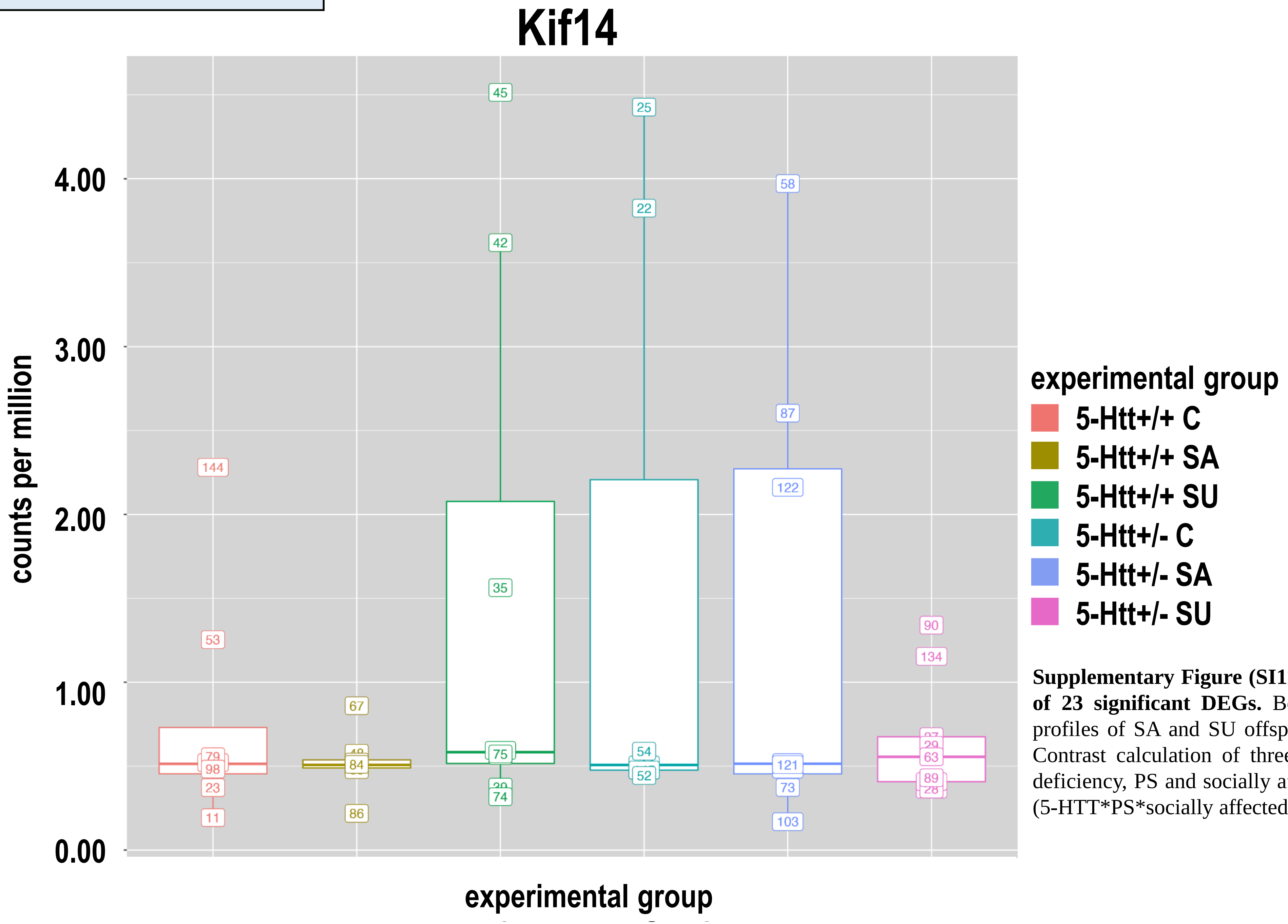

Supplementary Figure (SI11). Normalized read counts of 23 significant DEGs. Boxplots showing expression profiles of SA and SU offspring groups compared to C. Contrast calculation of three-way interaction of 5-HTT deficiency, PS and socially affected/unaffected behaviour (5-HTT*PS*socially affected/unaffected behaviour).

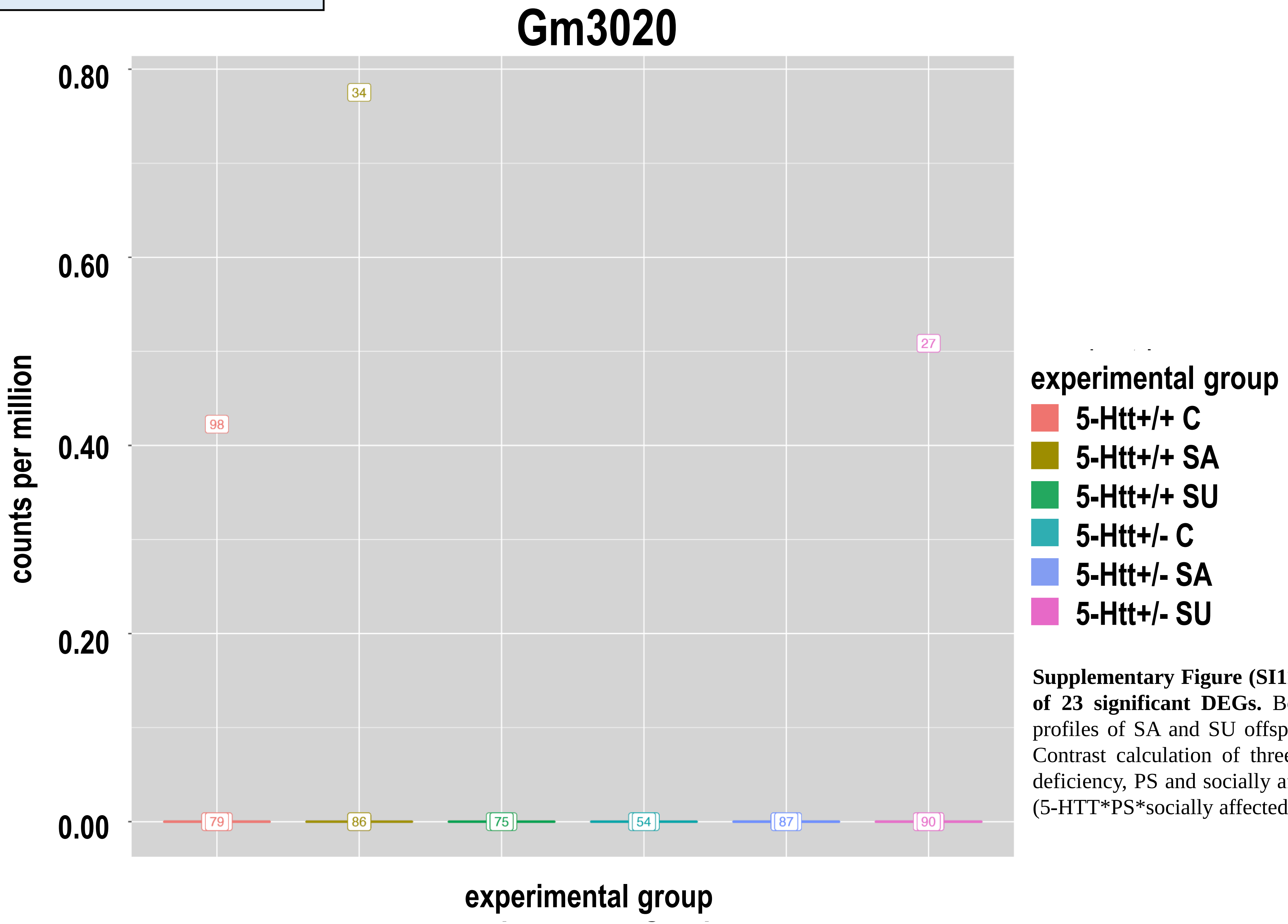

Supplementary Figure (SI11). Normalized read counts of 23 significant DEGs. Boxplots showing expression profiles of SA and SU offspring groups compared to C. Contrast calculation of three-way interaction of 5-HTT deficiency, PS and socially affected/unaffected behaviour (5-HTT*PS*socially affected/unaffected behaviour).

Supplementary Figure SI11: normalized read counts

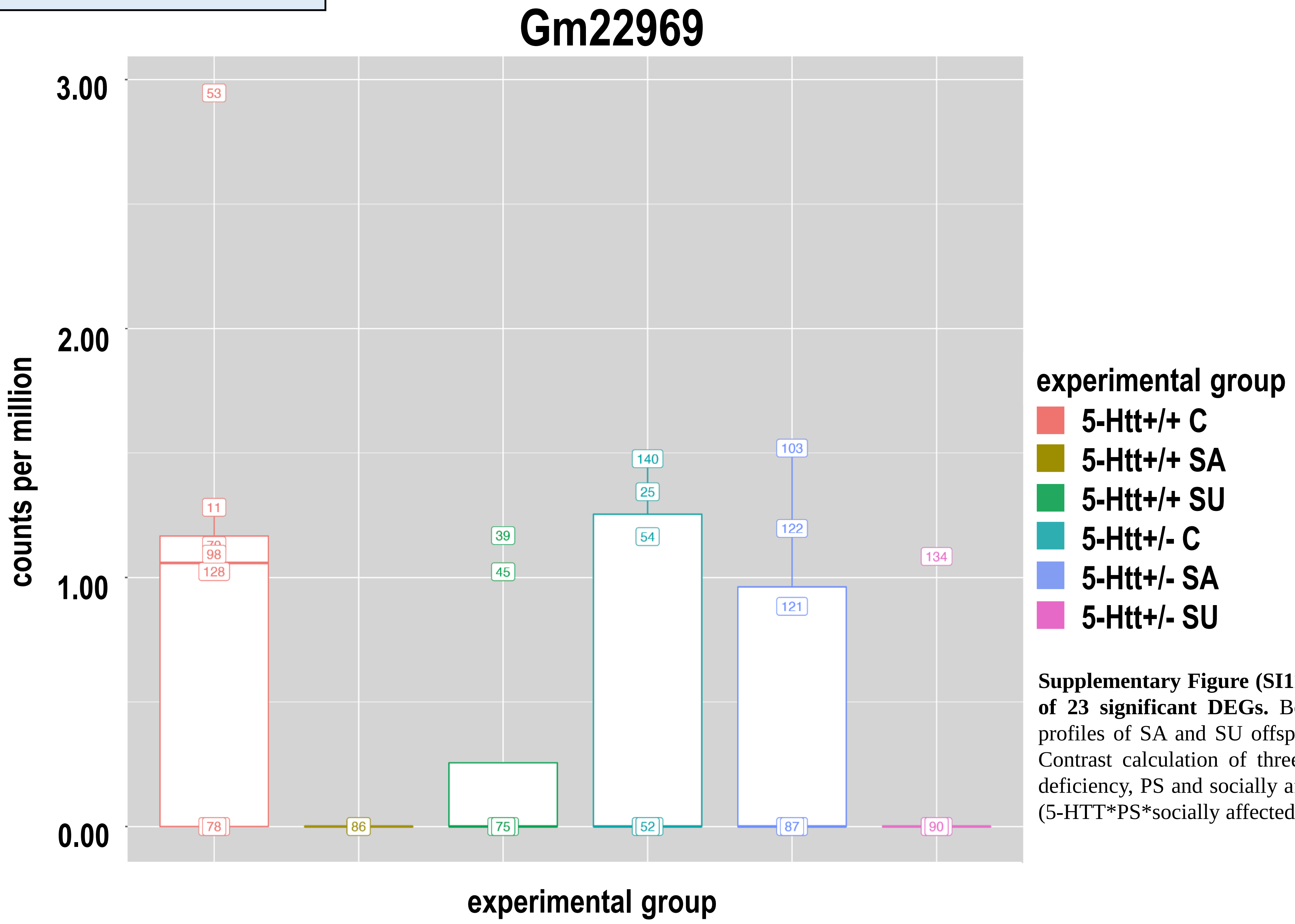

Supplementary Figure (SI11). Normalized read counts of 23 significant DEGs. Boxplots showing expression profiles of SA and SU offspring groups compared to C. Contrast calculation of three-way interaction of 5-HTT deficiency, PS and socially affected/unaffected behaviour (5-HTT*PS*socially affected/unaffected behaviour).

Supplementary Figure SI11: normalized read counts

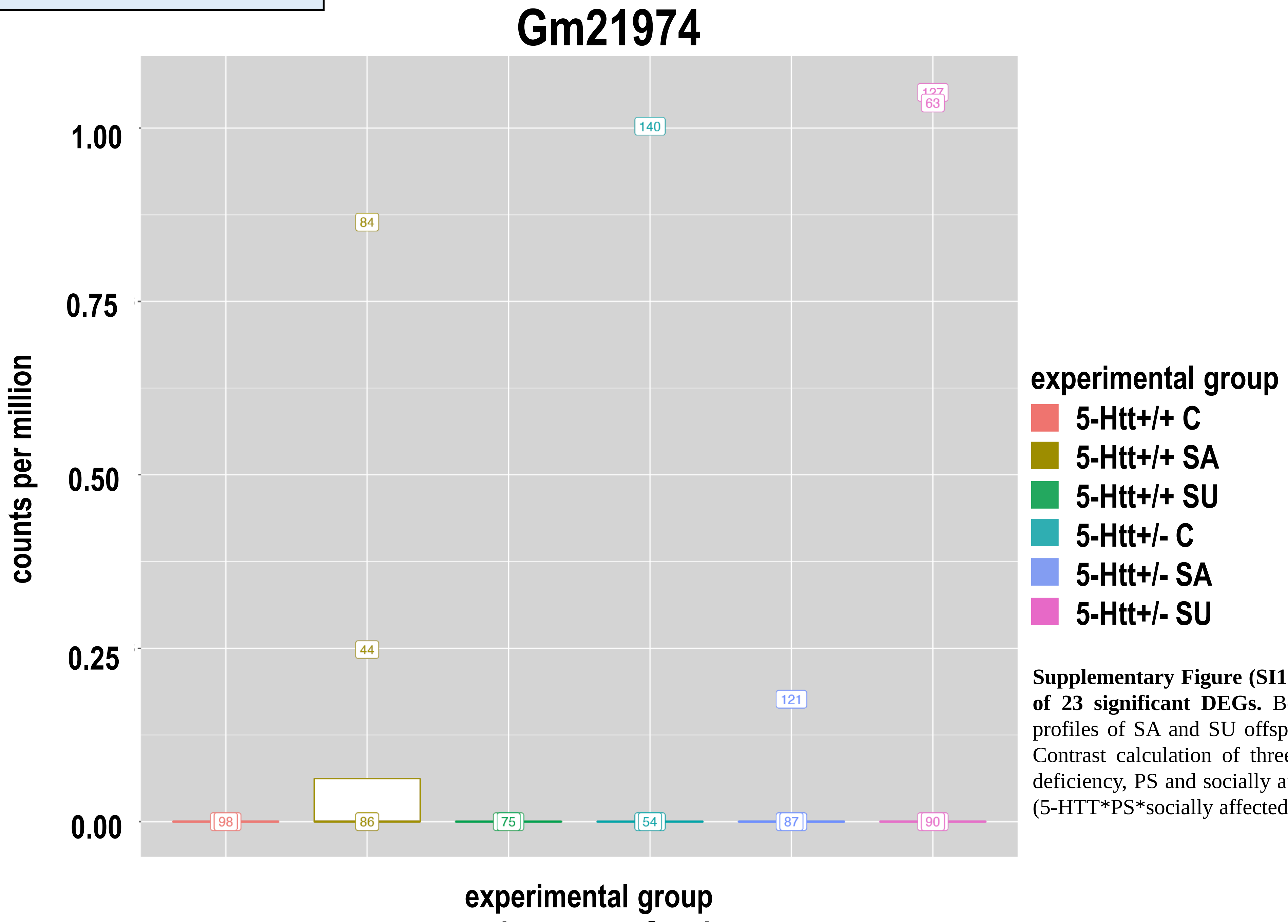

Supplementary Figure (SI11). Normalized read counts of 23 significant DEGs. Boxplots showing expression profiles of SA and SU offspring groups compared to C. Contrast calculation of three-way interaction of 5-HTT deficiency, PS and socially affected/unaffected behaviour (5-HTT*PS*socially affected/unaffected behaviour).

Supplementary Figure SI11: normalized read counts

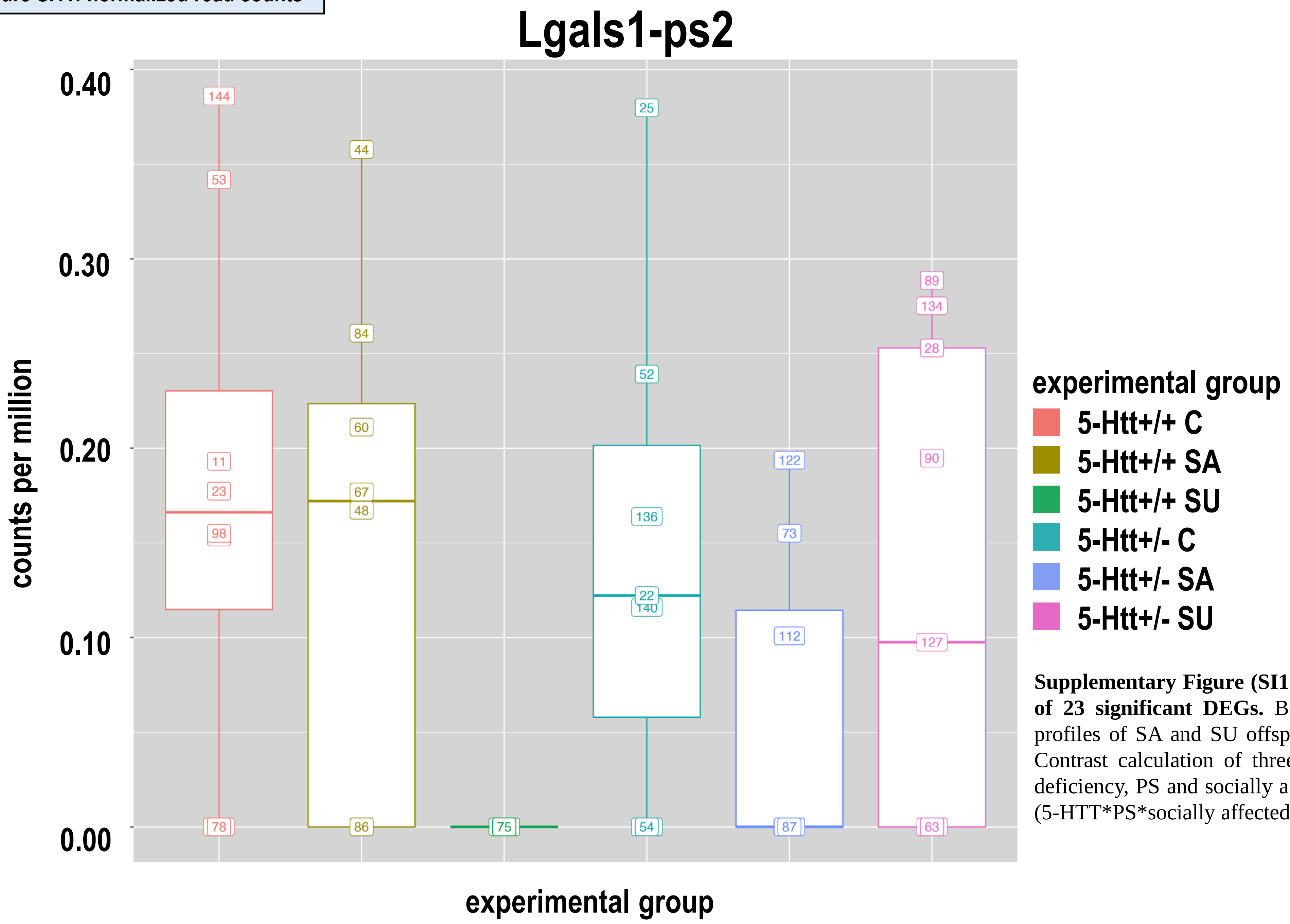

Supplementary Figure (SI11). Normalized read counts of 23 significant DEGs. Boxplots showing expression profiles of SA and SU offspring groups compared to C. Contrast calculation of three-way interaction of 5-HTT deficiency, PS and socially affected/unaffected behaviour (5-HTT*PS*socially affected/unaffected behaviour).

Supplementary Figure SI11: normalized read counts

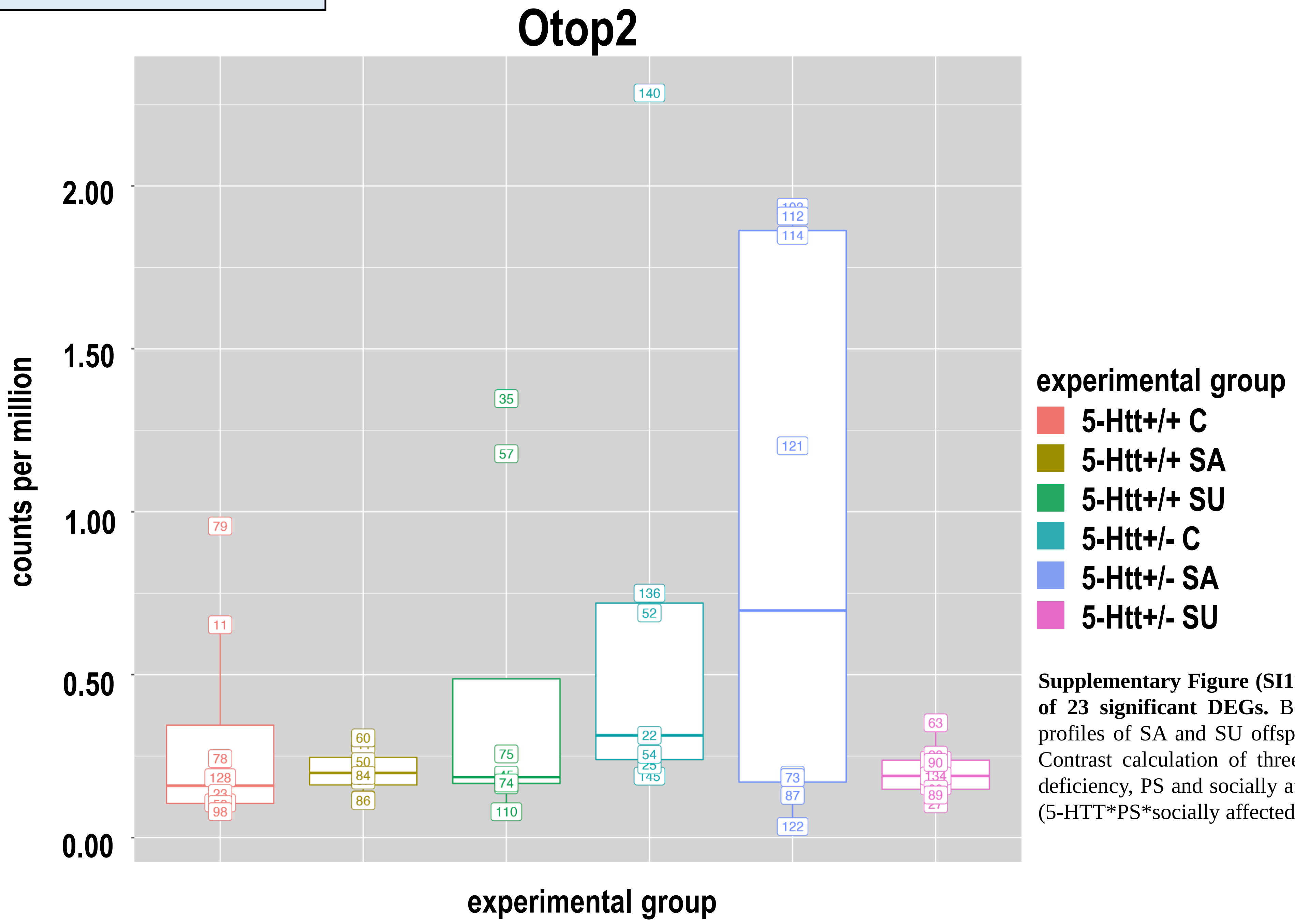

Supplementary Figure (SI11). Normalized read counts of 23 significant DEGs. Boxplots showing expression profiles of SA and SU offspring groups compared to C. Contrast calculation of three-way interaction of 5-HTT deficiency, PS and socially affected/unaffected behaviour (5-HTT*PS*socially affected/unaffected behaviour).

Supplementary Figure SI11: normalized read counts

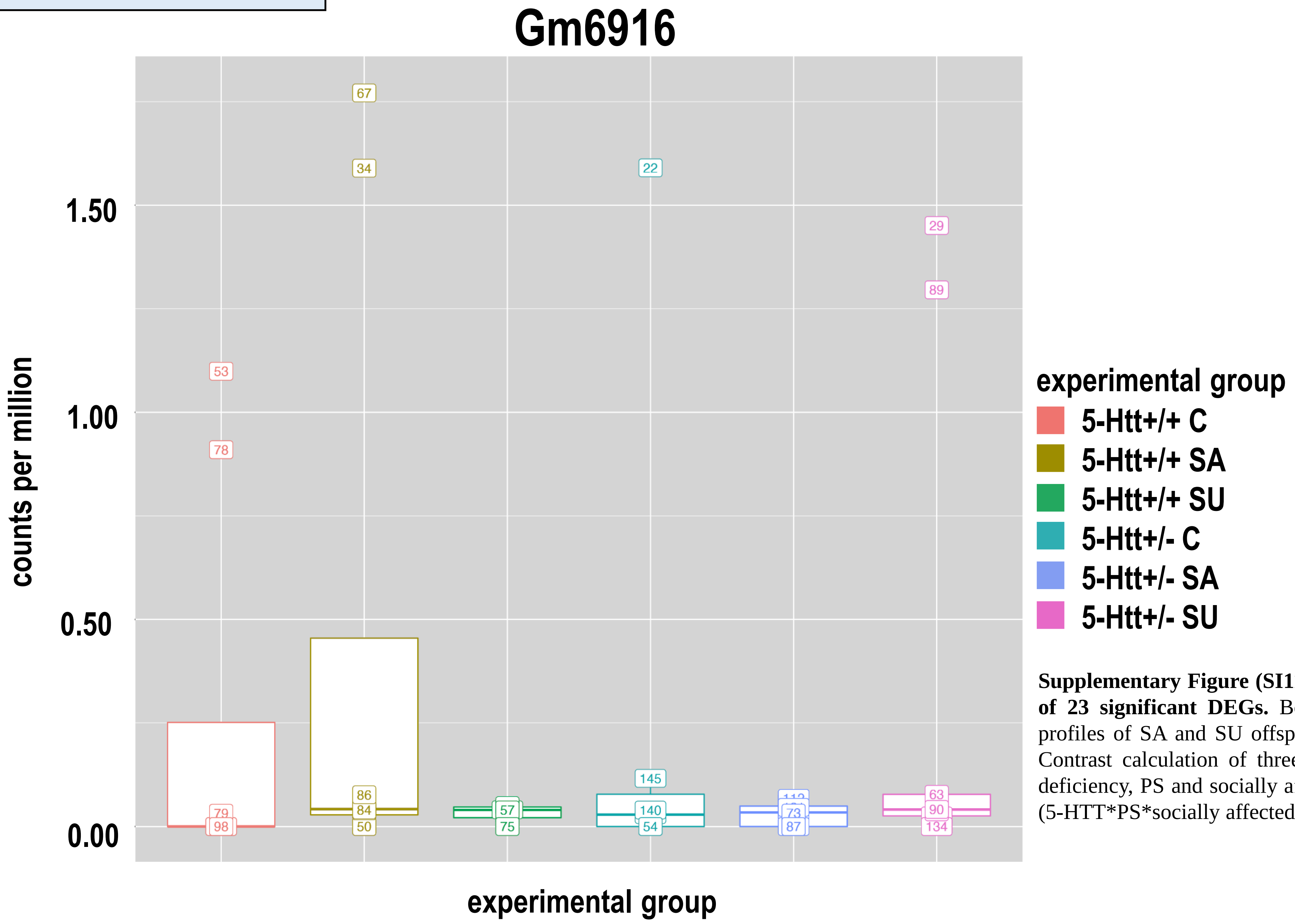

Supplementary Figure (SI11). Normalized read counts of 23 significant DEGs. Boxplots showing expression profiles of SA and SU offspring groups compared to C. Contrast calculation of three-way interaction of 5-HTT deficiency, PS and socially affected/unaffected behaviour (5-HTT*PS*socially affected/unaffected behaviour).

Supplementary Figure SI11: normalized read counts

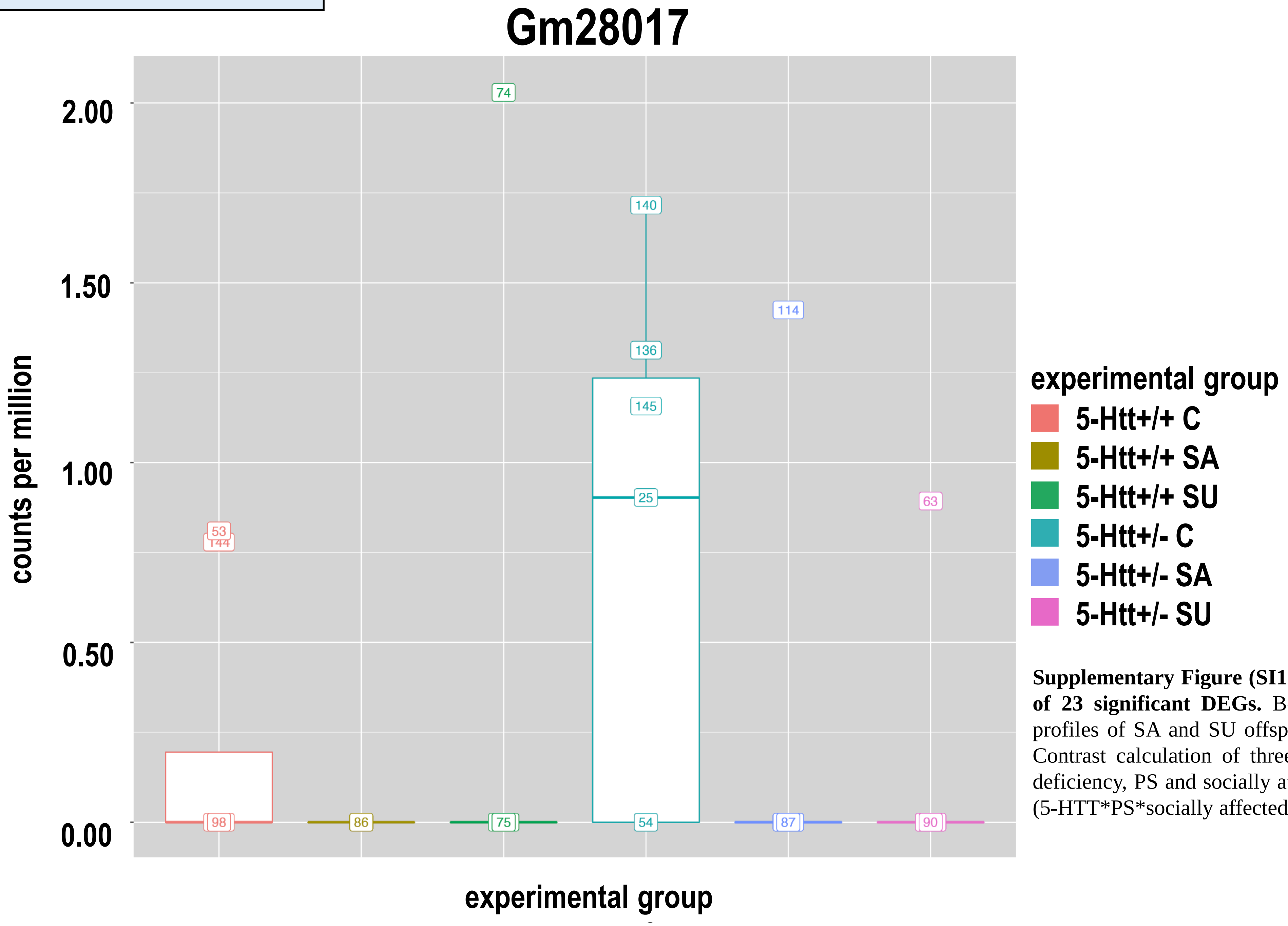

Supplementary Figure (SI11). Normalized read counts of 23 significant DEGs. Boxplots showing expression profiles of SA and SU offspring groups compared to C. Contrast calculation of three-way interaction of 5-HTT deficiency, PS and socially affected/unaffected behaviour (5-HTT*PS*socially affected/unaffected behaviour).

Supplementary Figure SI11: normalized read counts

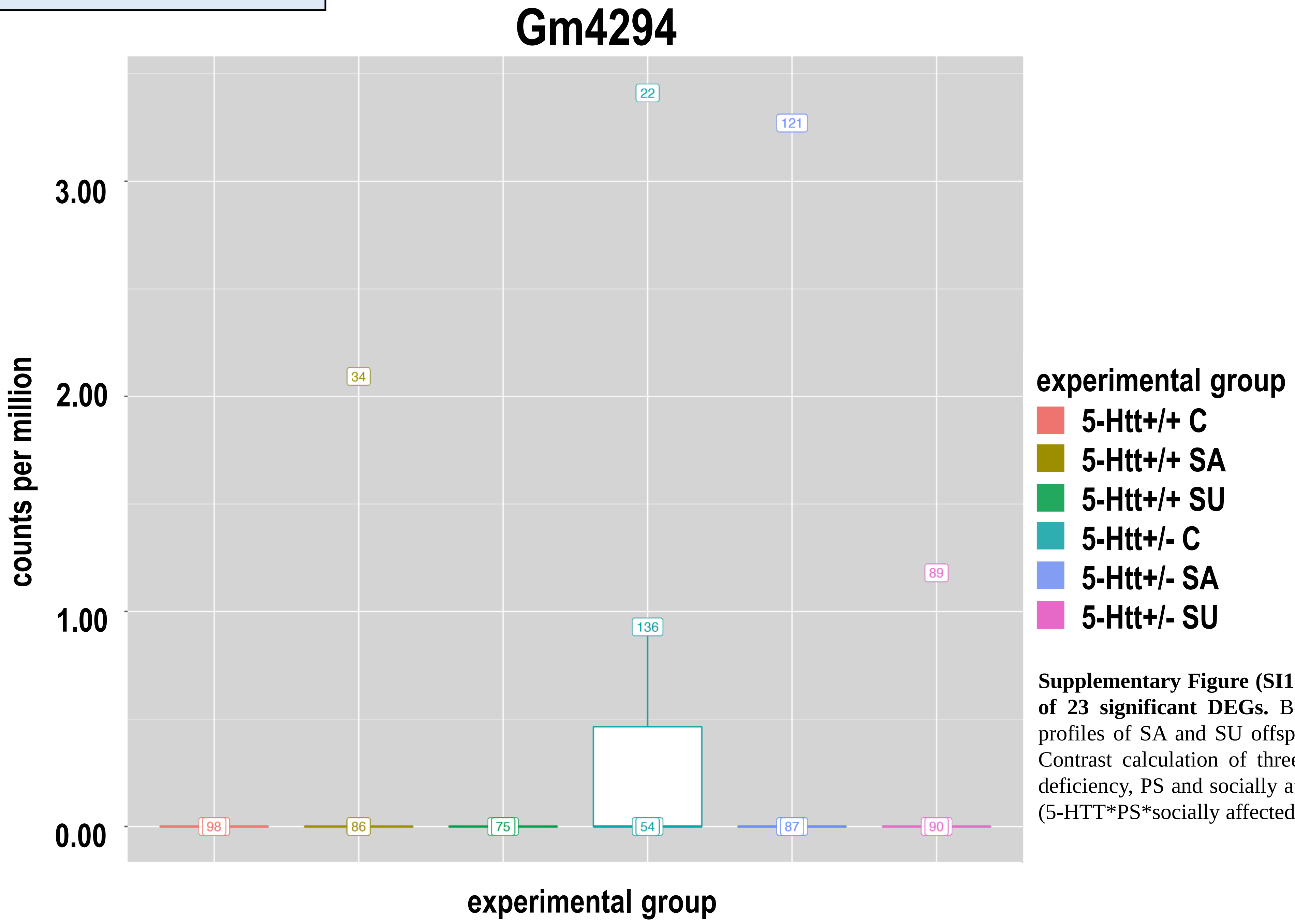

Supplementary Figure (SI11). Normalized read counts of 23 significant DEGs. Boxplots showing expression profiles of SA and SU offspring groups compared to C. Contrast calculation of three-way interaction of 5-HTT deficiency, PS and socially affected/unaffected behaviour (5-HTT*PS*socially affected/unaffected behaviour).

Supplementary Figure SI11: normalized read counts

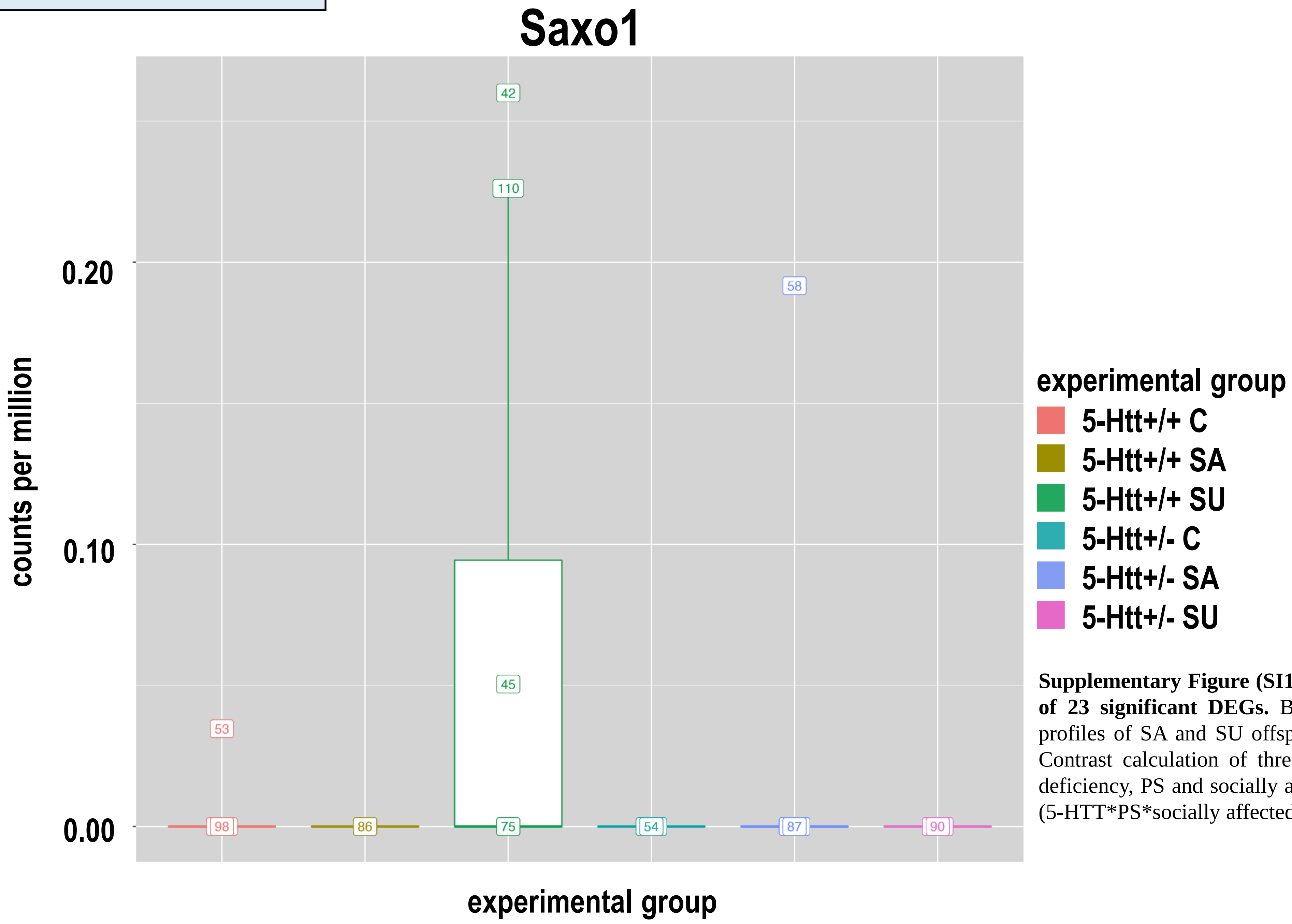

Supplementary Figure (SI11). Normalized read counts of 23 significant DEGs. Boxplots showing expression profiles of SA and SU offspring groups compared to C. Contrast calculation of three-way interaction of 5-HTT deficiency, PS and socially affected/unaffected behaviour (5-HTT*PS*socially affected/unaffected behaviour).

Supplementary Figure SI11: normalized read counts

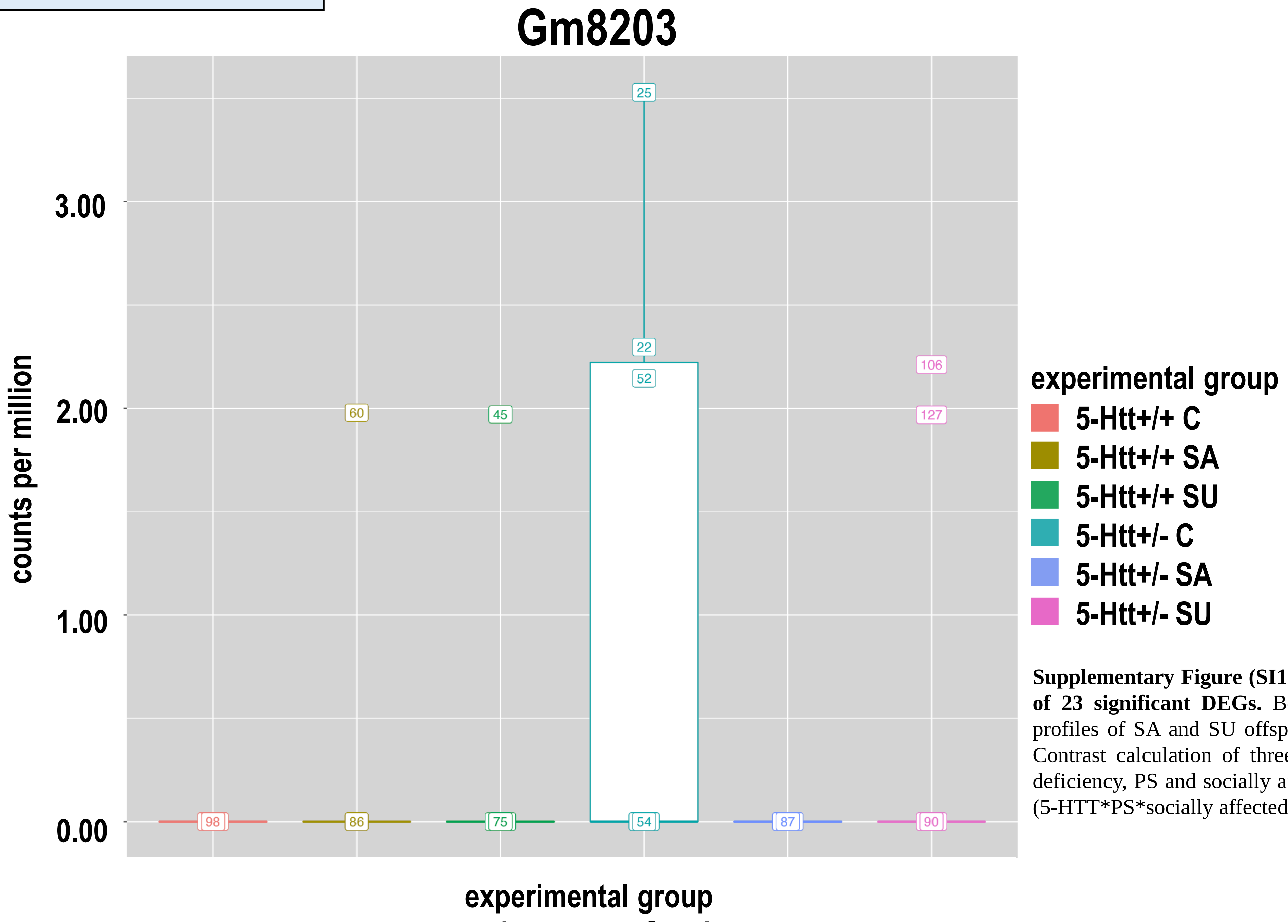

Supplementary Figure (SI11). Normalized read counts of 23 significant DEGs. Boxplots showing expression profiles of SA and SU offspring groups compared to C. Contrast calculation of three-way interaction of 5-HTT deficiency, PS and socially affected/unaffected behaviour (5-HTT*PS*socially affected/unaffected behaviour).

Supplementary Figure SI11: normalized read counts

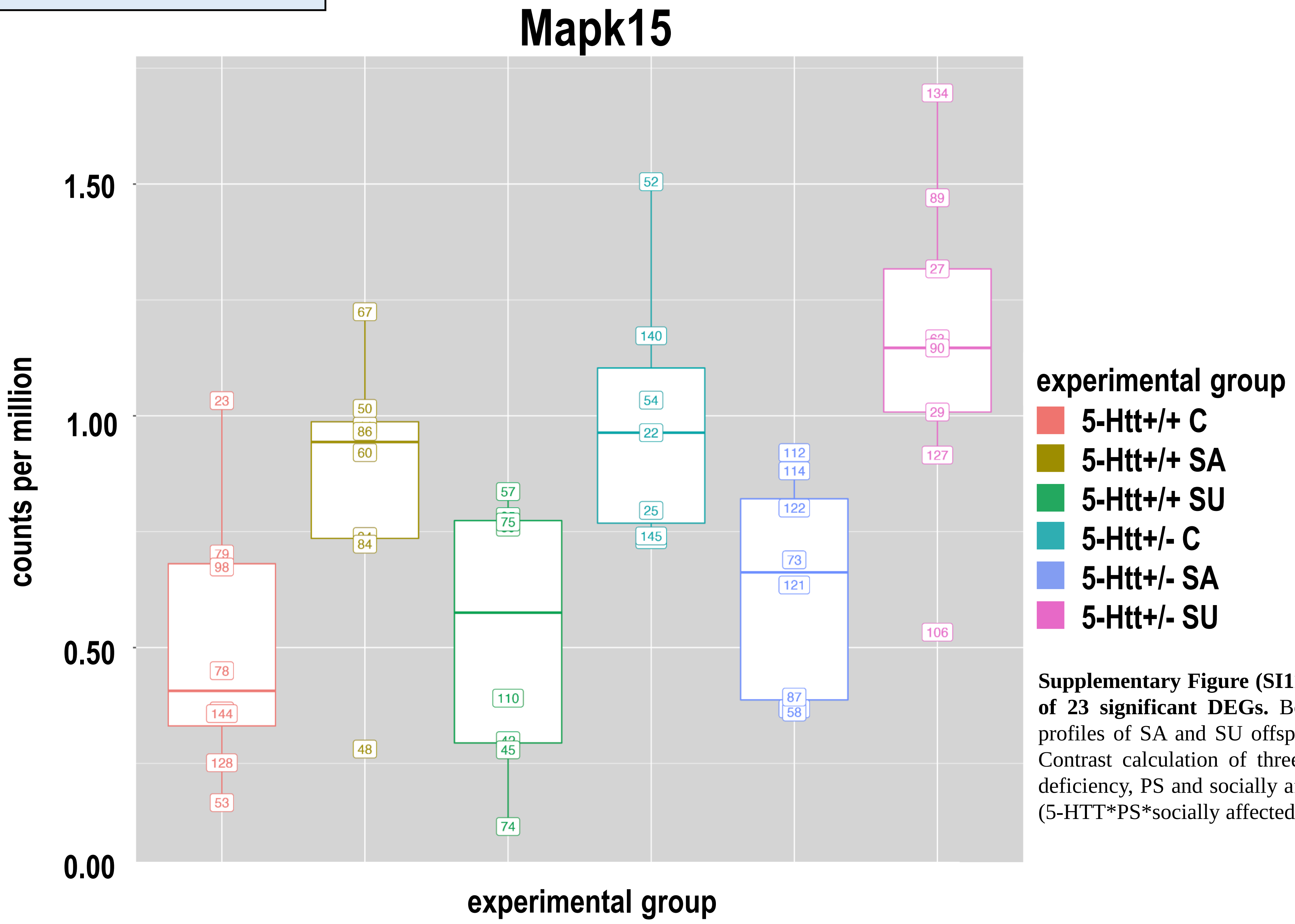

Supplementary Figure (SI11). Normalized read counts of 23 significant DEGs. Boxplots showing expression profiles of SA and SU offspring groups compared to C. Contrast calculation of three-way interaction of 5-HTT deficiency, PS and socially affected/unaffected behaviour (5-HTT*PS*socially affected/unaffected behaviour).

Supplementary Figure SI11: normalized read counts

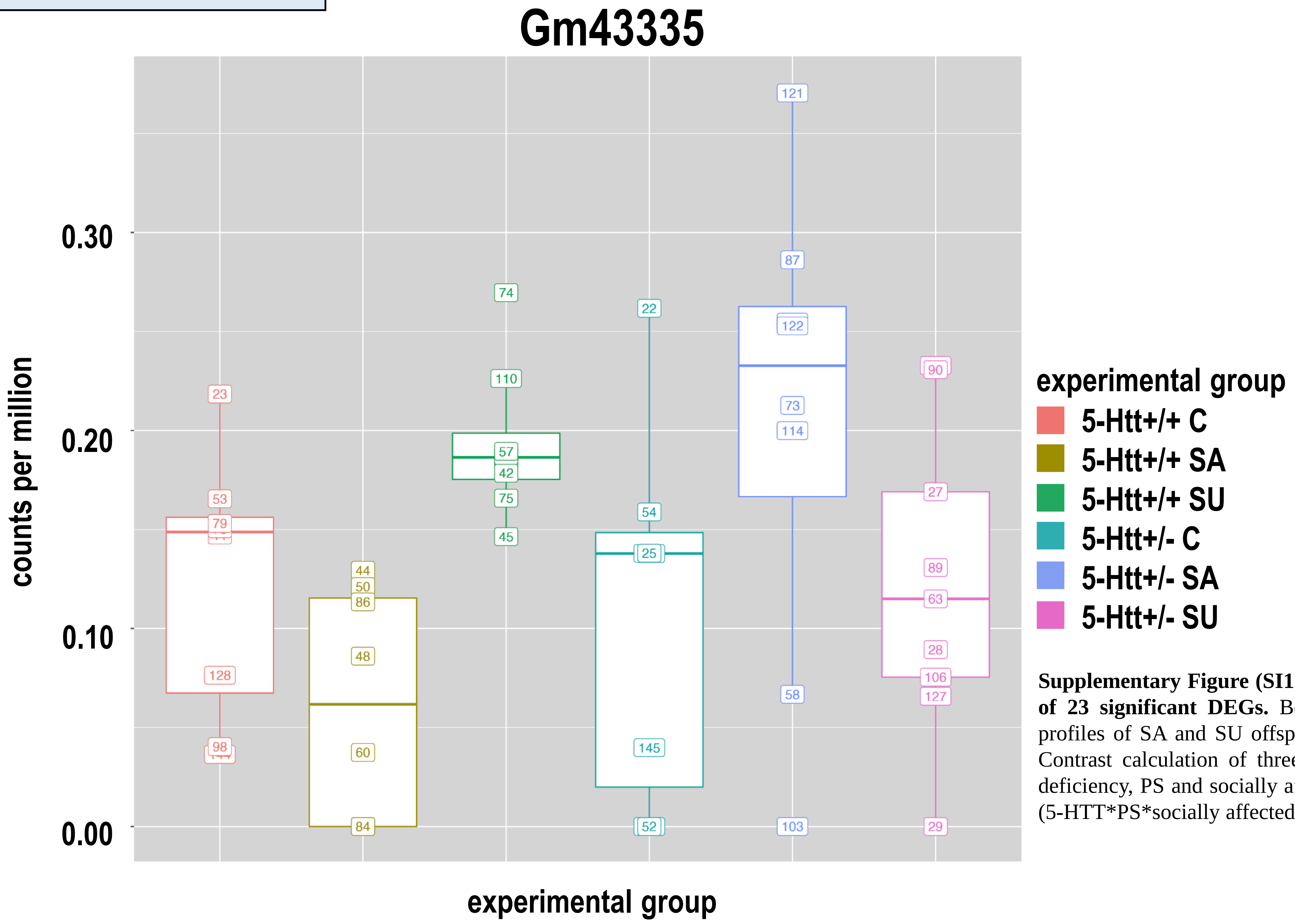

Supplementary Figure (SI11). Normalized read counts of 23 significant DEGs. Boxplots showing expression profiles of SA and SU offspring groups compared to C. Contrast calculation of three-way interaction of 5-HTT deficiency, PS and socially affected/unaffected behaviour (5-HTT*PS*socially affected/unaffected behaviour).

Supplementary Figure SI11: normalized read counts

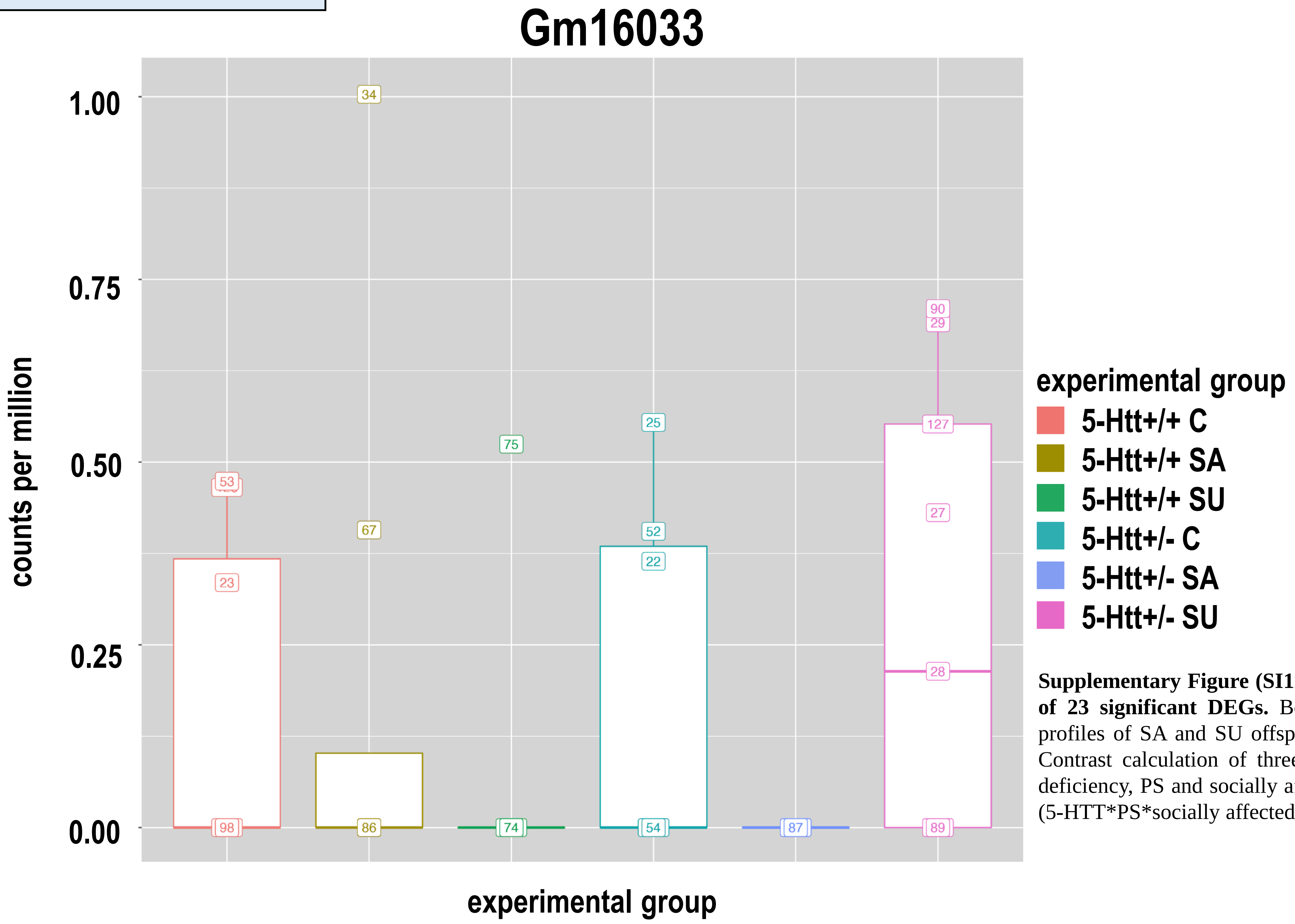

Supplementary Figure (SI11). Normalized read counts of 23 significant DEGs. Boxplots showing expression profiles of SA and SU offspring groups compared to C. Contrast calculation of three-way interaction of 5-HTT deficiency, PS and socially affected/unaffected behaviour (5-HTT*PS*socially affected/unaffected behaviour).
