# Supplementary figures and images for: Differential susceptibility to prenatal stress exposure in serotonin transporter-deficient female mice—an epigenetic exploration
Source: Front Neurosci. 2025 Sep 23;19:1633386. doi: 10.3389/fnins.2025.1633386 (PMC12500653; doi:10.3389/fnins.2025.1633386)

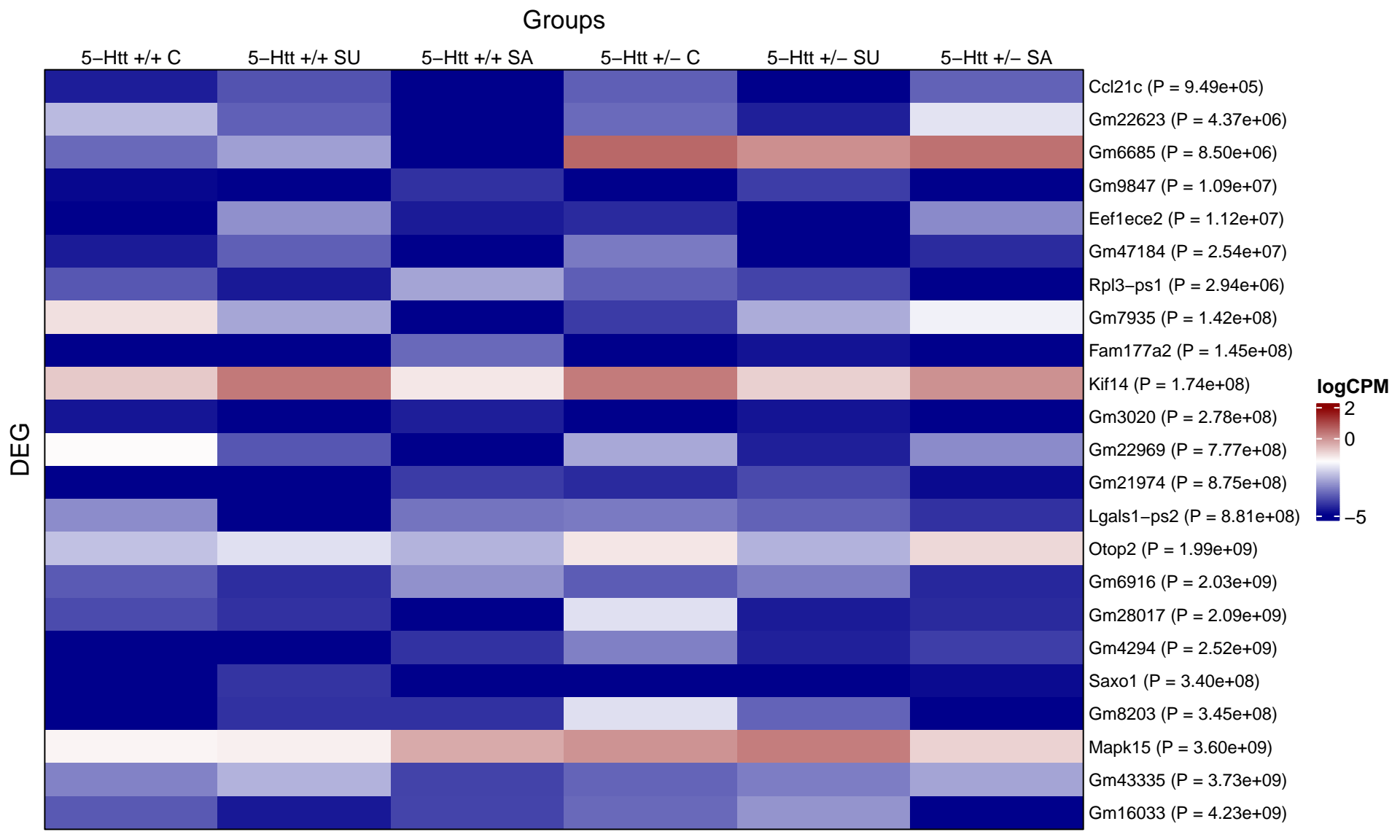

Supplement: Supplementary file 2 [file Data_Sheet_2.pdf]
