# Supplementary material for: Differential susceptibility to prenatal stress exposure in serotonin transporter-deficient female mice—an epigenetic exploration
Source: Front Neurosci. 2025 Sep 23;19:1633386. doi: 10.3389/fnins.2025.1633386 (PMC12500653; doi:10.3389/fnins.2025.1633386)
Supplement: Supplementary file 3 [file Supplementary_file_1.docx]

Supplementary Material

# Supplementary Data

**AFM and GAPDH as control primers for successful H3K4me3 chromatin immunoprecipitation**

To determine the successful immunoprecipitation of the histone marker H3K4me3, which is very closely related to actively expressed genes, we chose as a positive control glyceraldehyde 3-phosphate dehydrogenase (*Gapdh*). *Gapdh* is widely used as a reference gene for qPCR. As a negative control we used afamin (*Afm*), which is not expressed in the brain of adult mice. Both primer pairs were located in the area of the transcription start site and are displayed in SI1.

UCSC *In-Silico* PCR^[[1]](#footnote-1)^ using the reference genome Mouse July 2007 (NCBI37/mm9) Assembly and the temperature calculations were done assuming 50 mM salt and 50 nM annealing oligo concentration. The code to calculate the melting temperature was obtained from [Primer3](http://frodo.wi.mit.edu/primer3/input.htm) (Untergasser et al., 2012).

GAPDH:

>chr6:125115771-125115841 71 bp

Primer Melting Temperatures of forward (63.3°C) ctgggcctctctcatttccc and reverse (64.2°C) cttggtgcgtgcacatttca primer.

The PCR product:

CTGGGCCTCTCTCATTTCCCctcctccctctctttggacccgcctcattttTGAAATGTGCACGCACCAAG

is displayed in SI2.

AFM:

>chr5:90948075-90948146 72 bp

Primer Melting Temperatures of forward (61.0°C) ctagccctacccacaaagcc and reverse (60.7°C) cccctaacttagcattcctggt primer.

The PCR product:

CTAGCCCTACCCACAAAGCCtcaggacgtaggtaagtttacttgtgtattACCAGGAATGCTAAGTTAGGGG

is displayed in SI3.

All samples were run in duplicate and the used fluorophore was SYBRgreen. QRT-PCR was performed on the LC4800, 384 well system (Roche, Basel, Switzerland). For one sample per group, we additionally used an input control (IN) as negative control. This IN was not subjected to immunoprecipitation as positive control and samples, but immunoprecipitated with a mixture of random rabbit IgGs (RAB) (Vector Laboratories, Burlingame, CA, USA). For the IN fraction, 100 µl were taken from the RAB samples (SI4).

Results of the qRT-PCR are reported in table SI5.

**Mapping efficiency of the H3K4me3-based enrichment sequencing**

Sequencing quality, GC content, overrepresented sequences and sequencing duplication levels were all in accordance with the expected thresholds, while mapping showed some deviations. The paired-end 50 bp sequencing reads were mapped using Bowtie2 (v2.3.2) software. Table SI6 summarizes the mapping statistics for all samples. As the mapping efficiencies of some of the samples were lower than expected, a couple of additional improvement steps were performed. During this process possible bias in unmapped or poorly mapped reads was assessed. However, no technical bias could be found both in data analysis steps or library prep. The results of this extra analysis suggest that the mapping efficiency bias is merely caused by the sample preparation (chromatin immunoprecipitation; ChIP) itself and the data should be interpreted with this in mind, as the coverage might differ between the individual samples.

**Dam weight and litter size**

Dam weight was recorded on E0, E13 and E17. Before stress exposure control dams and dams that were appointed to the stress group showed the same gain in weight from E0 to E13 (see SI7 for more details). From beginning to end of the maternal stress paradigm (E13-E17), stressed dams showed reduced weight gain, when compared to control animals. The average litter size did not differ between groups.

The efficacy of the PS paradigm can be seen by looking at the dam weight. A significant reduction in weight gain during the last stage of pregnancy was observed in dams, subjected to restraint stress in comparison to control dams, while both groups produced on average litters of equal size. Repeated, acute, restraint stress has been widely associated with a decrease in body weight in rodents (Rybkin et al., 1997, Harris et al., 2002, Harris et al., 2006, Jeong et al., 2013).

# Supplementary Figures and Tables

## Supplementary Tables

SI1 Primer designed for positive and negative control of successful, specific immunoprecipitation of H3K4me

| Oligo Name | Sequence 5' to 3' (include modification codes if applicable) | Scale (µmol) | Purification | Company |
| --- | --- | --- | --- | --- |
| mmu-gDNA-Gapdh F' | CTGGGCCTCTCTCATTTCCC | 0.025 | desalted | Sigma-Aldrich |
| mmu-gDNA-Gapdh R' | CTTGGTGCGTGCACATTTCA | 0.025 | desalted | Sigma-Aldrich |
| mmu_gDNA_AFM_F | CTAGCCCTACCCACAAAGCC | 0.025 | desalted | Sigma-Aldrich |
| mmu_gDNA_AFM_R | CCCCTAACTTAGCATTCCTGGT | 0.025 | desalted | Sigma-Aldrich |

SI4 Summary of sequenced samples, n = 6 per group.

| **NXT-Dx No** | **Sample ID** | **Group** |
| --- | --- | --- |
| 1 | 78 | 1 |
| 2 | 128 | 1 |
| 3 | 53IN | 1 |
| 4 | 53 | 1 |
| 5 | 26 | 1 |
| 6 | 23 | 1 |
| 7 | 79 | 1 |
| 8 | 84 | 2 |
| 9 | 50 | 2 |
| 10 | 48 | 2 |
| 11 | 34 | 2 |
| 12 | 60IN | 2 |
| 13 | 60 | 2 |
| 14 | 66 | 2 |
| 15 | 110 | 3 |
| 16 | 75 | 3 |
| 17 | 42 | 3 |
| 18 | 45 | 3 |
| 19 | 117 | 3 |
| 20 | 57IN | 3 |
| 21 | 57 | 3 |
| 22 | 54IN | 4 |
| 23 | 54 | 4 |
| 24 | 139 | 4 |
| 25 | 52 | 4 |
| 26 | 17 | 4 |
| 27 | 22 | 4 |
| 28 | 25 | 4 |
| 29 | 103 | 5 |
| 30 | 122IN | 5 |
| 31 | 122 | 5 |
| 32 | 121 | 5 |
| 33 | 76 | 5 |
| 34 | 58 | 5 |
| 35 | 37 | 5 |
| 36 | 127IN | 6 |
| 37 | 127 | 6 |
| 38 | 106 | 6 |
| 39 | 63 | 6 |
| 40 | 90 | 6 |
| 41 | 27 | 6 |
| 42 | 89 | 6 |

SI5 Quality Control H3K4me3 ChIP. Positive and negative control for the specificity of the chromatin immuno precipitation using afamin (Afm) as negative and glyceraldehyde 3-phosphate dehydrogenase (Gapdh) as positive control.

| **Row** | **Column** | **Target** | **Sample** | **Plate-pos** | **Cp** | **Row** | **Column** | **Target** | **Sample** | **Plate-pos** | **Cp** |
| --- | --- | --- | --- | --- | --- | --- | --- | --- | --- | --- | --- |
| A | 9 | AFM | 17 | A9 | 35.73 | A | 1 | GAPDH | 17 | A1 | 30.4 |
| A | 10 | AFM | 17 | A10 | 34.85 | A | 2 | GAPDH | 17 | A2 | 30.8 |
| C | 9 | AFM | 22 | C9 | 35.55 | C | 1 | GAPDH | 22 |  | 32.6 |
| C | 10 | AFM | 22 | C10 |  | C | 2 | GAPDH | 22 | C2 | 30.74 |
| E | 9 | AFM | 23 | E9 |  | E | 1 | GAPDH | 23 |  | 31.13 |
| E | 10 | AFM | 23 | E10 |  | E | 2 | GAPDH | 23 | E2 | 31.28 |
| G | 9 | AFM | 25 | G9 |  | G | 1 | GAPDH | 25 |  | 31.6 |
| G | 10 | AFM | 25 | G10 |  | G | 2 | GAPDH | 25 | G2 | 31.55 |
| I | 9 | AFM | 26 | I9 | 35.64 | I | 1 | GAPDH | 26 |  | 31.19 |
| I | 10 | AFM | 26 | I10 |  | I | 2 | GAPDH | 26 | I2 | 31.07 |
| K | 9 | AFM | 27 | K9 |  | K | 1 | GAPDH | 27 |  | 30.45 |
| K | 10 | AFM | 27 | K10 | 35.8 | K | 2 | GAPDH | 27 | K2 | 30.72 |
| M | 9 | AFM | 34 | M9 | 34.71 | M | 1 | GAPDH | 34 |  | 30.96 |
| M | 10 | AFM | 34 | M10 |  | M | 2 | GAPDH | 34 | M2 | 30.67 |
| O | 9 | AFM | 35 | O9 | 47.59 | O | 1 | GAPDH | 35 |  | - |
| O | 10 | AFM | 35 | O10 |  | O | 2 | GAPDH | 35 | O2 | - |
| B | 9 | AFM | 37 | B9 | 35.72 | B | 1 | GAPDH | 37 |  | 31.39 |
| B | 10 | AFM | 37 | B10 | 36.67 | B | 2 | GAPDH | 37 | B2 | 31.11 |
| D | 9 | AFM | 42 | D9 | 35.17 | D | 1 | GAPDH | 42 |  | 31.54 |
| D | 10 | AFM | 42 | D10 |  | D | 2 | GAPDH | 42 | D2 | 31.54 |
| F | 9 | AFM | 45 | F9 | 34.8 | F | 1 | GAPDH | 45 |  | 31.35 |
| F | 10 | AFM | 45 | F10 | 35.78 | F | 2 | GAPDH | 45 | F2 | 31.74 |
| H | 9 | AFM | 48 | H9 | 35.15 | H | 1 | GAPDH | 48 |  | 30.34 |
| H | 10 | AFM | 48 | H10 |  | H | 2 | GAPDH | 48 | H2 | 30.33 |
| J | 9 | AFM | 50 | J9 |  | J | 1 | GAPDH | 50 |  | 30.06 |
| J | 10 | AFM | 50 | J10 | 35.72 | J | 2 | GAPDH | 50 | J2 | 30.31 |
| L | 9 | AFM | 52 | L9 |  | L | 1 | GAPDH | 52 |  | 30.13 |
| L | 10 | AFM | 52 | L10 | 34.72 | L | 2 | GAPDH | 52 | L2 | 29.75 |
| O | 11 | AFM | 58 | O11 | 40.71 | O | 3 | GAPDH | 58 | O3 | 32.61 |
| O | 12 | AFM | 58 | O12 |  | O | 4 | GAPDH | 58 | O4 | 32.62 |
| H | 11 | AFM | 63 | H11 | 40.55 | H | 3 | GAPDH | 63 | H3 | 32 |
| H | 12 | AFM | 63 | H12 | 35.59 | H | 4 | GAPDH | 63 | H4 | 31.69 |
| J | 11 | AFM | 66 | J11 | 35.84 | J | 3 | GAPDH | 66 | J3 | 30.3 |
| J | 12 | AFM | 66 | J12 | 36.46 | J | 4 | GAPDH | 66 | J4 | 30.03 |
| L | 11 | AFM | 75 | L11 | 35.66 | L | 3 | GAPDH | 75 | L3 | 31.82 |
| L | 12 | AFM | 75 | L12 |  | L | 4 | GAPDH | 75 | L4 | 33.1 |
| N | 11 | AFM | 76 | N11 |  | N | 3 | GAPDH | 76 | N3 | 33.62 |
| N | 12 | AFM | 76 | N12 | 37.15 | N | 4 | GAPDH | 76 | N4 | 33.4 |
| P | 12 | AFM | 78 | P12 |  | P | 3 | GAPDH | 78 | P3 | 30.88 |
| P | 11 | AFM | 78 | P11 |  | P | 4 | GAPDH | 78 | P4 | 30.69 |
| A | 13 | AFM | 79 | A13 | 34.87 | A | 5 | GAPDH | 79 | A5 | 30.97 |
| A | 14 | AFM | 79 | A14 |  | A | 6 | GAPDH | 79 | A6 | 30.79 |
| C | 13 | AFM | 84 | C13 | 42.08 | C | 5 | GAPDH | 84 | C5 | 30.71 |
| C | 14 | AFM | 84 | C14 | 35.55 | C | 6 | GAPDH | 84 | C6 | 30.72 |
| E | 13 | AFM | 86 | E13 | 34.85 | E | 5 | GAPDH | 86 | E5 | 33.65 |
| E | 14 | AFM | 86 | E14 | 35.86 | E | 6 | GAPDH | 86 | E6 | 32.78 |
| G | 13 | AFM | 89 | G13 |  | G | 5 | GAPDH | 89 | G5 | 30.77 |
| G | 14 | AFM | 89 | G14 |  | G | 6 | GAPDH | 89 | G6 | 30.18 |
| I | 13 | AFM | 90 | I13 | 34.53 | I | 5 | GAPDH | 90 | I5 | 31.51 |
| I | 14 | AFM | 90 | I14 | 35.07 | I | 6 | GAPDH | 90 | I6 | 31.9 |
| K | 15 | AFM | 103 | K15 |  | K | 7 | GAPDH | 103 | K7 | 30.61 |
| K | 16 | AFM | 103 | K16 |  | K | 8 | GAPDH | 103 | K8 | 30.62 |
| K | 13 | AFM | 106 | K13 |  | K | 5 | GAPDH | 106 | K5 | 31.83 |
| K | 14 | AFM | 106 | K14 |  | K | 6 | GAPDH | 106 | K6 | 31.51 |
| M | 13 | AFM | 110 | M13 | 45.88 | M | 5 | GAPDH | 110 | M5 | 32.45 |
| M | 14 | AFM | 110 | M14 | 36.91 | M | 6 | GAPDH | 110 | M6 | 34.39 |
| O | 13 | AFM | 111 | O13 | 35.19 | O | 5 | GAPDH | 111 | O5 | 32.77 |
| O | 14 | AFM | 111 | O14 | 33.92 | O | 6 | GAPDH | 111 | O6 | 32.65 |
| B | 13 | AFM | 112 | B13 |  | B | 5 | GAPDH | 112 | B5 | 37.05 |
| B | 14 | AFM | 112 | B14 |  | B | 6 | GAPDH | 112 | B6 | 35.59 |
| D | 13 | AFM | 117 | D13 |  | D | 5 | GAPDH | 117 | D5 | 32.77 |
| D | 14 | AFM | 117 | D14 |  | D | 6 | GAPDH | 117 | D6 | 33.13 |
| F | 13 | AFM | 121 | F13 |  | F | 5 | GAPDH | 121 | F5 | 31.41 |
| F | 14 | AFM | 121 | F14 |  | F | 6 | GAPDH | 121 | F6 | 31.23 |
| C | 15 | AFM | 128 | C15 |  | C | 7 | GAPDH | 128 | C7 | 32.1 |
| C | 16 | AFM | 128 | C16 |  | C | 8 | GAPDH | 128 | C8 | 31.22 |
| E | 15 | AFM | 139 | E15 |  | E | 7 | GAPDH | 139 | E7 | 33 |
| E | 16 | AFM | 139 | E16 |  | E | 8 | GAPDH | 139 | E8 | 32.99 |
| G | 15 | AFM | 145 | G15 | 35.18 | G | 7 | GAPDH | 145 | G7 | 32.94 |
| G | 16 | AFM | 145 | G16 | 46.87 | G | 8 | GAPDH | 145 | G8 | 33.22 |
| I | 15 | AFM | 147 | I15 | 47.65 | I | 7 | GAPDH | 147 | I7 | 34.36 |
| I | 16 | AFM | 147 | I16 | 34.54 | I | 8 | GAPDH | 147 | I8 | 33.34 |
| L | 13 | AFM | 122 IN | L13 | 26.66 | L | 5 | GAPDH | 122 IN | L5 | 27.85 |
| L | 14 | AFM | 122 IN | L14 | 26.7 | L | 6 | GAPDH | 122 IN | L6 | 27.87 |
| H | 13 | AFM | 122 IP | H13 | 43.87 | H | 5 | GAPDH | 122 IP | H5 | 30.68 |
| H | 14 | AFM | 122 IP | H14 |  | H | 6 | GAPDH | 122 IP | H6 | 30.97 |
| J | 13 | AFM | 122 RAB | J13 |  | J | 5 | GAPDH | 122 RAB | J5 |  |
| J | 14 | AFM | 122 RAB | J14 | 34.67 | J | 6 | GAPDH | 122 RAB | J6 |  |
| A | 15 | AFM | 127 IN | A15 | 27.04 | A | 7 | GAPDH | 127 IN | A7 | 28.56 |
| A | 16 | AFM | 127 IN | A16 | 26.97 | A | 8 | GAPDH | 127 IN | A8 | 28.46 |
| N | 13 | AFM | 127 IP | N13 |  | N | 5 | GAPDH | 127 IP | N5 | 32.22 |
| N | 14 | AFM | 127 IP | N14 |  | N | 6 | GAPDH | 127 IP | N6 | 31.36 |
| P | 13 | AFM | 127 RAB | P13 | 36.77 | P | 5 | GAPDH | 127 RAB | P5 |  |
| P | 14 | AFM | 127 RAB | P14 | 35.71 | P | 6 | GAPDH | 127 RAB | P6 |  |
| A | 11 | AFM | 53 IN | A11 | 27.1 | A | 3 | GAPDH | 53 IN | A3 | 28.42 |
| A | 12 | AFM | 53 IN | A12 | 27.5 | A | 4 | GAPDH | 53 IN | A4 | 28.62 |
| N | 9 | AFM | 53 IP | N9 |  | N | 1 | GAPDH | 53 IP |  |  |
| N | 10 | AFM | 53 IP | N10 |  | N | 2 | GAPDH | 53 IP | N2 | 31.47 |
| P | 9 | AFM | 53 RAB | P9 |  | P | 1 | GAPDH | 53 RAB |  |  |
| P | 10 | AFM | 53 RAB | P10 |  | P | 2 | GAPDH | 53 RAB | P2 |  |
| G | 11 | AFM | 54 IN | G11 | 27.33 | G | 3 | GAPDH | 54 IN | G3 | 28.11 |
| G | 12 | AFM | 54 IN | G12 | 26.65 | G | 4 | GAPDH | 54 IN | G4 | 27.89 |
| C | 11 | AFM | 54 IP | C11 | 35.95 | C | 3 | GAPDH | 54 IP | C3 | 30800 |
| C | 12 | AFM | 54 IP | C12 |  | C | 4 | GAPDH | 54 IP | C4 | 30.55 |
| E | 11 | AFM | 54 RAB | E11 | 39.29 | E | 3 | GAPDH | 54 RAB | E3 |  |
| E | 12 | AFM | 54 RAB | E12 | 35.71 | E | 4 | GAPDH | 54 RAB | E4 | 37.75 |
| M | 11 | AFM | 57 IN | M11 | 28.37 | M | 3 | GAPDH | 57 IN | M3 | 29.53 |
| M | 12 | AFM | 57 IN | M12 | 27.85 | M | 4 | GAPDH | 57 IN | M4 | 29.3 |
| I | 11 | AFM | 57 IP | I11 |  | I | 3 | GAPDH | 57 IP | I3 | 30.27 |
| I | 12 | AFM | 57 IP | I12 | 35.87 | I | 4 | GAPDH | 57 IP | I4 | 29.96 |
| K | 11 | AFM | 57 RAB | K11 | 42.73 | K | 3 | GAPDH | 57RAB | K3 | 36.21 |
| K | 12 | AFM | 57 RAB | K12 |  | K | 4 | GAPDH | 57 RAB | K4 |  |
| F | 11 | AFM | 60 IN | F11 | 26.47 | F | 3 | GAPDH | 60 IN | F3 | 27.63 |
| F | 12 | AFM | 60 IN | F12 | 26.62 | F | 4 | GAPDH | 60 IN | F4 | 27.74 |
| B | 11 | AFM | 60 IP | B11 | 35.62 | B | 3 | GAPDH | 60 IP | B3 | 30.06 |
| B | 12 | AFM | 60 IP | B12 | 35.72 | B | 4 | GAPDH | 60 IP | B4 | 29.74 |
| D | 11 | AFM | 60 RAB | D11 | 33.59 | D | 3 | GAPDH | 60 RAB | D3 | 34.43 |
| D | 12 | AFM | 60 RAB | D12 | 34.11 | D | 4 | GAPDH | 60 RAB | D4 | 35.26 |
| M | 15 | AFM | TE | M15 |  | M | 7 | GAPDH | TE | M7 |  |
| M | 16 | AFM | TE | M16 |  | M | 8 | GAPDH | TE | M8 |  |

SI6 Table Mapping efficiency of the sequencing using Bowtie.2. The table shows the fragment length (FL), treatment redundancy (TR), control redundancy (CR), efficiency of the mapping (aligned [%]), furthermore the % of duplicates (Dup) and GC content [%] and total sequences in millions (M Seq) for reads 1 and 2 of the paired-end reads.

| **Sample** | **FL** | **TR** | **CR** | **Aligned [%]** | **Dup1 [%]** | **GC1 [%]** | **M Seq1** | **Dup2 [%]** | **GC2 [%]** | **M Seq2** |
| --- | --- | --- | --- | --- | --- | --- | --- | --- | --- | --- |
| 17 IP | 232 | 0.21 |  | 30.5 | 13 | 46 | 32.7 | 11.4 | 46 | 32.7 |
| 22 IP | 221 | 0.18 |  | 31.7 | 11.9 | 47 | 22.5 | 10.7 | 48 | 22.5 |
| 23 IP | 232 | 0.22 |  | 51.8 | 10.4 | 50 | 13.2 | 9.7 | 50 | 13.2 |
| 25 IP | 232 | 0.28 |  | 54.8 | 18.3 | 53 | 22.9 | 15.9 | 53 | 22.9 |
| 26 IP | 233 | 0.18 |  | 43.9 | 8.5 | 50 | 10.1 | 7.8 | 50 | 10.1 |
| 27 IP | 224 | 0.21 |  | 41.6 | 10.9 | 48 | 15.6 | 10.2 | 48 | 15.6 |
| 34 IP | 235 | 0.2 |  | 42.3 | 9.9 | 48 | 15.8 | 8.9 | 48 | 15.8 |
| 37 IP | 234 | 0.22 |  | 50.6 | 11.3 | 49 | 16 | 10.5 | 49 | 16 |
| 42 IP | 220 | 0.25 |  | 56.9 | 13.4 | 52 | 15.3 | 12.4 | 52 | 15.3 |
| 45 IP | 220 | 0.22 |  | 49.9 | 12.1 | 49 | 16.4 | 11.4 | 49 | 16.4 |
| 48 IP | 224 | 0.23 |  | 57.3 | 14 | 50 | 15.8 | 12.3 | 50 | 15.8 |
| 50 IP | 232 | 0.19 |  | 48 | 9 | 49 | 10.8 | 8.3 | 49 | 10.8 |
| 52 IP | 226 | 0.24 |  | 78.9 | 15 | 51 | 24.3 | 13.3 | 51 | 24.3 |
| 53 control | 220 | 0.2 | 0.19 |  |  |  |  |  |  |  |
| 53 IN | 71 | 0.19 |  | 37.4 | 11.6 | 45 | 26.4 | 10.3 | 45 | 26.4 |
| 53 IP | 220 | 0.2 |  | 62.2 | 11.6 | 49 | 12.9 | 10 | 49 | 12.9 |
| 54 control | 219 | 0.2 | 0.21 |  |  |  |  |  |  |  |
| 54 IN | 73 | 0.21 |  | 46.5 | 12.3 | 44 | 23.8 | 11.8 | 45 | 23.8 |
| 54 IP | 219 | 0.2 |  | 59.1 | 12.3 | 49 | 22.6 | 11 | 49 | 22.6 |
| 57 control | 230 | 0.21 | 0.21 |  |  |  |  |  |  |  |
| 57 IN | 70 | 0.21 |  | 35.6 | 10.8 | 45 | 23.5 | 10.4 | 45 | 23.5 |
| 57 IP | 230 | 0.21 |  | 54.8 | 11 | 50 | 18.7 | 10.3 | 50 | 18.7 |
| 58 IP | 213 | 0.19 |  | 30.1 | 10.7 | 47 | 15.2 | 10 | 47 | 15.2 |
| 60 control | 226 | 0.18 | 0.21 |  |  |  |  |  |  |  |
| 60 IN | 65 | 0.21 |  | 30.3 | 10.8 | 45 | 20.7 | 10.4 | 46 | 20.7 |
| 60 IP | 226 | 0.18 |  | 51.7 | 10 | 48 | 15.1 | 9.5 | 48 | 15.1 |
| 63 IP | 210 | 0.22 |  | 33 | 11.3 | 45 | 14.1 | 10.4 | 45 | 14.1 |
| 66 IP | 229 | 0.18 |  | 43.2 | 10 | 48 | 15.9 | 9.5 | 48 | 15.9 |
| 75 IP | 216 | 0.21 |  | 51.2 | 11.2 | 50 | 14.6 | 10.4 | 50 | 14.6 |
| 76 IP | 222 | 0.28 |  | 53.5 | 17.7 | 54 | 13 | 16.5 | 54 | 13 |
| 78 IP | 217 | 0.2 |  | 56.5 | 9.5 | 51 | 13.1 | 8.6 | 51 | 13.1 |
| 79 IP | 239 | 0.22 |  | 78.4 | 11.7 | 52 | 11.7 | 10.9 | 52 | 11.7 |
| 84 IP | 233 | 0.21 |  | 65.7 | 10.2 | 52 | 11.3 | 9.5 | 52 | 11.3 |
| 89 IP | 232 | 0.27 |  | 58.1 | 13.3 | 51 | 15.8 | 12.6 | 51 | 15.8 |
| 90 IP | 208 | 0.22 |  | 36.8 | 11.9 | 46 | 19.5 | 11.2 | 46 | 19.5 |
| 103 IP | 216 | 0.21 |  | 51.1 | 13 | 50 | 23.9 | 11.6 | 50 | 23.9 |
| 106 IP | 212 | 0.25 |  | 49.5 | 13.1 | 49 | 19.7 | 11.9 | 49 | 19.7 |
| 110 IP | 201 | 0.18 |  | 36.1 | 10.3 | 45 | 18 | 9.6 | 45 | 18 |
| 117 IP | 214 | 0.22 |  | 40.4 | 12.8 | 49 | 16.9 | 12 | 49 | 16.9 |
| 121 IP | 221 | 0.28 |  | 57.8 | 16.3 | 52 | 23.4 | 14.6 | 52 | 23.4 |
| 122 control | 224 | 0.27 | 0.19 |  |  |  |  |  |  |  |
| 122 IN | 69 | 0.19 |  | 38 | 11 | 45 | 26.2 | 10.2 | 45 | 26.2 |
| 122 IP | 224 | 0.27 |  | 75.8 | 17.2 | 54 | 21.4 | 15.2 | 54 | 21.4 |
| 127 control | 221 | 0.28 | 0.2 |  |  |  |  |  |  |  |
| 127 IN | 68 | 0.2 |  | 32 | 11.1 | 45 | 26.9 | 10.8 | 45 | 26.9 |
| 127 IP | 221 | 0.28 |  | 68.1 | 17 | 53 | 15.2 | 15.6 | 53 | 15.2 |
| 128 IP | 215 | 0.23 |  | 57.4 | 10.9 | 50 | 14.9 | 10.3 | 50 | 14.9 |
| 139 IP | 230 | 0.2 |  | 44.5 | 12.8 | 49 | 20.1 | 11.3 | 49 | 20.1 |

SI7 Table Group sizes, weight [g] of dams from embryonic day 0 to embryonic day 17 and weight gain (E13-E17, [%]), as well as litter size [n]. The displayed values represent group means ± standard errors. Statistical significance (p ≤ 0.05) as tested using the Kruskal-Wallis test is indicated by an asterisk. PS=prenatal stress, C=control, E=embryonic day

| **Condition** | **n** | **weight E0** | **weight E13** | **weight E17*** | **weight gain [%]*** | **n˚ pups** |
| --- | --- | --- | --- | --- | --- | --- |
| C | 14 | 20.14 ± 0.20 | 30.82 ± 0.47 | 38.18 ± 0.85 | 19.4±0.01 | 7.36 ± 0.46 |
| PS | 22 | 20.29 ± 0.20 | 30.82 ± 0.32 | 35.75 ± 0.42 | 13.6±0.01 | 7.64 ± 0.31 |

**SI8 Statistical analysis of differentially expressed genes (DEG) in the context of a three-way interaction of serotonin transporter (5-HTT) deficiency, PS and socially affected (SA)/unaffected behaviour (SU) (5-HTT*PS*socially affected/unaffected behaviour) by means of the calculated contrast:** [(5-*Htt+/+*SA vs *5-Htt+/+*C) vs (*5-Htt+/+*SU vs *5-Htt+/+*C)] vs [(*5-Htt+/-*SA vs *5-Htt+/-*C) vs (*5-Htt+/-*SU vs *5-Htt+/-*C)]. Nominal DEGs.

[Table SI8 from supplementary excel sheet]

**SI9 significant DEGs**

[Table SI9 from supplementary excel sheet]

**SI10 Heatmap logCPM** The heatmap shows the expression levels (logCPM) of differentially expressed genes (DEGs) identified through a three-way interaction between serotonin transporter (5-HTT) deficiency, prenatal stress (PS), and sociability phenotype (socially affected (SA) vs. unaffected (SU)). The contrast used to identify DEGs was:[(5-Htt+/+ SA vs 5-Htt+/+ C) vs (5-Htt+/+ SU vs 5-Htt+/+ C)] vs [(5-Htt+/- SA vs 5-Htt+/- C) vs (5-Htt+/- SU vs 5-Htt+/- C)]. Columns represent the experimental groups included in the interaction analysis. Rows display the identified DEGs, with corresponding p-values indicated next to each gene name.

[Figure S10 from supplementary PDF]#

**SI11. Normalized read counts of 23 significant DEGs.** Boxplots showing expression profiles of SA and SU offspring groups compared to C. Contrast calculation of three-way interaction of 5-HTT deficiency, PS and socially affected/unaffected behaviour (5-HTT*PS*socially affected/unaffected behaviour).

[Figure SI11 from supplementary PDF]

SI12 Descriptive statistics and statistical analysis of the most relevant behavioural parameters of the 3-Chamber Sociability test, the Elevated Plus Maze and the Porsolt’s Swim Test as well as the Sucrose Preference Test. Time is measured in seconds (s), distance is measured in millimetres (mm). Consumption of sucrose is measured as percentage [%] of sucrose solution consumed in view of the total volume consumed. The gene-by-environment interaction two-way ANOVA as well as the interaction of differential social behaviour following PS exposure and 5-Htt genotype two-way ANOVA were used as statistical tests. Kruskal-Wallis and Mann-Whitney-U tests were used as non-parametric test methods where applicable. The number of animals (n) included for each test is given in the table. Data represent mean ± SEM. Significant p-values are depicted in bold.

| **Physiological parameters: interaction effect of genotype and social susceptibility to prenatal stress** | | | | | | | | | | | | | | | | | | | | | | | | |
| --- | --- | --- | --- | --- | --- | --- | --- | --- | --- | --- | --- | --- | --- | --- | --- | --- | --- | --- | --- | --- | --- | --- | --- | --- |
|  | ***5Htt+/+*** | | | | | | | | | | | | ***5Htt+/-*** | | | | | | | | | | | |
| Parameter | Control | | | Prenatal stress groups | | | | | | | | | Control | | | Prenatal stress groups | | | | | | | | |
|  |  | | | all prenatal stress | | | socially affected | | | socially unaffected | | |  | | | all prenatal stress | | | socially affected | | | socially unaffected | | |
| **Sucrose Preference Test** | | | | | | | | | | | | | | | | | | | | | | | | |
|  | *N* | *Mean* | *SEM* | *N* | *Mean* | *SEM* | *N* | *Mean* | *SEM* | *N* | *Mean* | *SEM* | *N* | *Mean* | *SEM* | *N* | *Mean* | *SEM* | *N* | *Mean* | *SEM* | *N* | *Mean* | *SEM* |
| Sucrose/water consumption [ml] | 19 | 3.57 | ±0.2 | 35 | 3.81 | ±0.1 | 9 | 4.21 | ±0.2 | 26 | 3.68 | ±0.14 | 20 | 3.42 | ±0.12 | 41 | 3.29 | ±0.12 | 15 | 3.46 | ±0.16 | 26 | 3.19 | ±0.16 |
| **Corticosterone** | | | | | | | | | | | | | | | | | | | | | | | | |
|  | *N* | *Mean* | *SEM* | *N* | *Mean* | *SEM* | *N* | *Mean* | *SEM* | *N* | *Mean* | *SEM* | *N* | *Mean* | *SEM* | *N* | *Mean* | *SEM* | *N* | *Mean* | *SEM* | *N* | *Mean* | *SEM* |
| Basal all | 20 | 130.3 | ±17.6 | 36 | 148.71 | ±14.9 | 10 | 139.31 | ±28.0 | 26 | 152.33 | ±17.9 | 20 | 121.17 | ±19.8 | 42 | 148.31 | ±15.8 | 16 | 187.84 | ±35.8 | 26 | 123.98 | ±11.2 |
| Basal | 19 | 127.11 | ±18.2 | 35 | 150.41 | ±15.3 | 10 | 139.31 | ±28.0 | 25 | 154.85 | ±18.5 | 20 | 121.17 | ±19.8 | 42 | 148.31 | ±15.8 | 16 | 187.84 | ±35.8 | 26 | 123.98 | ±11.2 |
| Stress | 19 | 634.17 | ±16.1 | 35 | 618.89 | ±13.4 | 10 | 650.81 | ±18.7 | 25 | 606.12 | ±16.7 | 20 | 604.83 | ±17.7 | 42 | 600.96 | ±15.2 | 16 | 639.63 | ±29.7 | 26 | 577.17 | ±15.0 |
| Diff | 19 | 507.07 | ±20.2 | 10 | 511.5 | ±24.4 | 25 | 451.27 | ±18.3 | 20 | 483.67 | ±22.4 | 42 | 452.65 | ±17.0 | 16 | 451.79 | ±35.7 | 10 | 511.5 | ±24.4 | 26 | 453.19 | ±17.2 |
|  | **Two way ANOVA**  **Interaction genotype * PS** | | | | | | | | | | | | **Two way ANOVA**  **Interaction genotype * social susceptibility** | | | | | | | | | | | |
| **Sucrose Preference Test** | | | | | | | | | | | | | | | | | | | | | | | | |
| Sucrose/water consumption [ml] | F_(1.111)_ = 1.6; p = 0.21 | | | | | | | | | | | | F_(2,109)_ = 1.28; p = 0.28 | | | | | | | | | | | |
| **Corticosterone** | | | | | | | | | | | | | | | | | | | | | | | | |
| Basal all | F_(1,114)_ = 0.06; p = 0.809 | | | | | | | | | | | | F_(2,112)_ = 1.49.; p = 0.229 | | | | | | | | | | | |
| Basal | F_(1,112)_ = 0.01; p = 0.917 | | | | | | | | | | | | F_(2,110)_ = 1.56; p = 0.216 | | | | | | | | | | | |
| Stress | F_(1,112)_ = 0.12; p = 0.735 | | | | | | | | | | | | F_(2,110)_ = 0.11; p = 0.894 | | | | | | | | | | | |
| Diff | F_(1,112)_ = 0.04; p = 0.847 | | | | | | | | | | | | F_(2,110)_ = 0.81; p = 0.448 | | | | | | | | | | | |

SI13 Descriptive statistics and statistical analysis of the most relevant behavioural parameters of the 3-Chamber Sociability test, the Elevated Plus Maze and the Porsolt’s Swim Test as well as the Sucrose Preference Test. Time is measured in seconds (s), distance is measured in millimetres (mm). Consumption of sucrose is measured as percentage [%] of sucrose solution consumed in view of the total volume consumed. The gene-by-environment interaction two-way ANOVA as well as the interaction of differential social behaviour following PS exposure and 5-Htt genotype two-way ANOVA were used as statistical tests. Kruskal-Wallis and Mann-Whitney-U tests were used as non-parametric test methods where applicable. The number of animals (n) included for each test is given in the table. Data represent mean ± SEM. Significant p-values are depicted in bold.

| **Interaction effects of genotype and social susceptibility to prenatal stress** | | | | | | | | |
| --- | --- | --- | --- | --- | --- | --- | --- | --- |
| Parameter | **Two way ANOVA**  **Interaction genotype * PS** | | **Two way ANOVA**  **Genotype** | | **Two way ANOVA**  **PS** | | **Kruskal-Wallis**  **Genotype * PS** | |
| **3-Chamber Sociability Test** | | | | | | | | |
| Time [s] in target chamber | F_(1.114)_ = 0.96; p = 0.33 | | F_(1.114)=_ 0.82; p = 0.37 | | F_(1.114)_ = 8.81 ; **p = 0.004** | | H_(3)_ = 8,416; **p = 0.038** | |
| **Elevated Plus Maze** | | | | | | | | |
| Time [s] in open arm | F_(1.112)_ = 0.14; p = 0.712 | | F_(1.112)_ = 0.47; p = 0.493 | | F_(1.112)_ = 6.6; **p = 0.012** | | H_(3)_ = 7.115; p =0.068 | |
| Time [s] in closed arm | F_(1.112)_ = 0.36; p = 0.552 | | F_(1.112)_ = 0.5; p = 0.48 | | F_(1.112)_ = 1.85; p = 0.177 | | H_(3)_ = 3.774; p = 0.290 | |
| Time [s] in center | F_(1.112)_ = 0.19; p = 0.661 | | F_(1.112)_ = 0.0003; p = 0.986 | | F_(1.112)_ = 6.32; **p = 0.013** | | H_(3)_ = 4.684; p = 0.196 | |
| Distance [mm] in open arm | F(_1.112)_ = 0.01; p = 0.946 | | F_(1.112)_ = 0.34; p = 0.564 | | F_(1.112)_ = 7.03; **p = 0.009** | | H_(3)_ = 7.486; p = 0.058 | |
| Distance [mm] in closed arm | F_(1.112)_ = 0.25; p = 0.616 | | F_(1.112)_ = 0.53; p = 0.468 | | F_(1.112)_ = 1.24; p = 0.269 | | H_(3)_ = 3.161; p = 0.367 | |
| Distance [mm] in center | F_(1.112)_ = 0.0001; p = 0.991 | | F_(1.112)_ = 0.007; p = 0.933 | | F_(1.112)_ = 3.61; p = 0.06 | | H_(3)_ = 2.618; p = 0.454 | |
| **Porsolts Swim Test** | | | | | | | | |
| Distance [mm] in total | F_(1.114)_ = 0.006; p = 0.94 | | F_(1.114)_ = 0.24 ; p = 0.625 | | F_(1.114)_ = 0.08; p = 0.782 | | H_(3)_ = 1.835; p = 0.607 | |
| **Sucrose Preference Test** | | | | | | | | |
| Relative sucrose amount consumed [%] | F_(1.111)_ = 0.62; p = 0.433 | | F_(1.111)_ = 1.83; p = 0.179 | | F_(1.111)_ = 0.0003; p = 0.986 | | H_(3)_ = 2.800; p = 0.423 | |
|  | **Mann-Whitney *U***  ***5-Htt+/+* C * PS** | | **Mann-Whitney *U***  ***5-Htt+/-* C * PS** | | **Mann-Whitney *U***  **C * PS** | | **Mann-Whitney *U***  ***5-Htt+/+* * *5-Htt+/-*** | |
| **3-Chamber Sociability Test** | | | | | | | | |
| Time [s] in target chamber | U = 254; **p = 0.012** | | U = 269; p = 0.12 | | U = 1078; **p = 0.006** | | U = 1650; p = 0.643 | |
| **Elevated Plus Maze** | | | | | | | | |
| Time [s] in open arm | U = 255; p = 0.124 | | U = 288, p = 0.084 | | U = 1064; **p = 0.014** | | U = 1475; p = 0.263 | |
| Time [s] in closed arm | U = 309; p = 0.559 | | U = 303; p = 0.135 | | U = 1215.5; p = 0.116 | | U = 1496; p = 0.316 | |
| Time [s] in center | U = 227.5; **p = 0.043** | | U = 350; p = 0.445 | | U = 1148.5; p = 0.05 | | U = 1675; p = 0.989 | |
| Distance [mm] in open arm | U = 265; p = 0.173 | | U = 258; **p = 0.028** | | U = 1045; **p = 0.01** | | U = 1511; p = 0.357 | |
| Distance [mm] in closed arm | U = 325; p = 0.763 | | U = 313; p = 0.180 | | U = 1267; p = 0.206 | | U = 1497; p = 0.318 | |
| Distance [mm] in center | U = 281; p = 0.280 | | U = 330; p = 0.283 | | U = 1217; p = 0.119 | | U = 1589; p = 0.625 | |
|  | **Two way ANOVA**  **Interaction genotype * social susceptibility** | | **Two way ANOVA**  **Social susceptibility** | | **Two way ANOVA**  **Genotype** | | **Kruskal-Wallis**  **Genotype * social susceptibility** | |
| **3-Chamber Sociability Test** | | | | | | | | |
| Time [s] in target chamber | F_(2.112)_ = 0.57; p = 0.565 | | F_(2.112)_ = 34.59 ; **p < 0.000** | | F_(2.112)_ = 1.29; p = 0.259 | | H_(5)_ = 55.009; **p < 0.000** | |
| **Elevated Plus Maze** | | | | | | | | |
| Time [s] in open arm | F_(2.110)_ = 5.68; **p = 0.004** | | F_(2.110)_ = 4.2; **p = 0.017** | | F_(2.110)_ = 0.02; p = 0.897 | | H_(5)_ = 14.233; **p = 0.014** | |
| Time [s] in closed arm | F_(2.110)_ = 3.64; **p = 0.03** | | F_(2.110)_ = 1.38; p = 0.256 | | F_(2.110)_ = 0.02; p = 0.890 | | H_(5)_ = 8.746; p = 0.120 | |
| Time [s] in center | F_(2.110)_ = 1.07; p = 0.345 | | F_(2.110)_ = 3.52; **p = 0.033** | | F_(2.110)_ = 0.28; p = 0.600 | | H_(5)_ 7.446; p = 0.190 | |
| Distance [mm] in open arm | F_(2.110)_ = 4.29; **p = 0.016** | | F_(2.110)_ = 4.7; **p = 0.011** | | F_(2.110)_ = 0.085; p = 0.772 | | H_(5)_ = 15.750; **p = 0.008** | |
| Distance [mm] in closed arm | F_(2.110)_ = 0.89; p = 0.416 | | F_(2.110)_ = 0.7; p = 0.498 | | F_(2.110)_ = 0.306; p = 0.581 | | H_(5)_ = 3.974; p = 0.553 | |
| Distance [mm] in center | F_(2.110)_ =0.33; p = 0.719 | | F_(2.110)_ = 0.01; p = 0.921 | | F_(2.110)_ = 2.02; p = 0.138 | | H_(5)_ = 3.880; p = 0.567 | |
| **Porsolts Swim Test** | | | | | | | | |
| Distance [mm] in total | F_(2.112)_ = 0.28; p = 0.76 | | F_(2.112)_ = 0.175; p = 0.839 | | F_(2.112)_ = 0.04; p= 0.835 | | H_(5)_ = 2.34; p = 0.8 | |
| **Sucrose Preference Test** | | | | | | | | |
| Relative sucrose amount consumed [%] | F_(2.109)_ = 0.36; p = 0.696 | | F_(2.109)_ = 0.11; p = 0.892 | | F_(2.109)_ = 1.54; p = 0.217 | | H_(5)_ = 3.276; p = 0.657 | |
|  | **Mann-Whitney U**  ***5-Htt+/+* SA * C** | **Mann-Whitney U**  ***5-Htt+/+* SU * C** | | **Mann-Whitney U**  ***5-Htt+/+* SA * SU** | **Mann-Whitney U**  ***5-Htt+/+* SA * C** | **Mann-Whitney U**  ***5-Htt+/-* SU * C** | | **Mann-Whitney U**  ***5-Htt+/-* SA * SU** |
| **3-Chamber Sociability Test** | | | | | | | | |
| Time [s] in target chamber | U = 12; **p < 0.000** | U = 257; p = 0.947 | | U <0.000; **p < 0.000** | U = 25; **p < 0.000** | U = 229; p = 0.492 | | U < 0.000; **p < 0.000** |
| **Elevated Plus Maze** | | | | | | | | |
| Time [s] in open arm | U = 38; **p = 0.009** | U = 217; p = 0.491 | | U = 73; **p = 0.044** | U = 138; p = 0.643 | U = 150; **p = 0.026** | | U = 152; p = 0.147 |
| Time [s] in closed arm | U = 56; p = 0.081 | U = 242; p = 0.909 | | U = 76; p = 0.056 | U = 137; p = 0.619 | U = 166; p = 0.063 | | U = 153; p = 0.154 |
| Time [s] in center | U = 52; **p = 0.048** | U = 175.5; p = 0.1 | | U = 110; p = 0.480 | U = 148; p = 0.895 | U = 194; p = 0.223 | | U = 147; p = 0.114 |
| Distance [mm] in open arm | U = 35; **p = 0.006** | U = 230; p = 0.696 | | U = 67; **p = 0.026** | U = 120; p = 0.289 | U = 138; **p = 0.012** | | U = 156; p = 0.178 |
| Distance [mm] in closed arm | U = 84; p = 0.614 | U = 241; p = 0.890 | | U = 116; p = 0.621 | U = 134; p = 0.551 | U = 179: p = 0.118 | | U = 180; p = 0.468 |
| Distance [mm] in center | U = 73; p = 0.313 | U = 208; p = 0.370 | | U = 120; p = 0.724 | U = 142; p = 0.741 | U = 188; p = 0.175 | | U = 168; p = 0.300 |

##
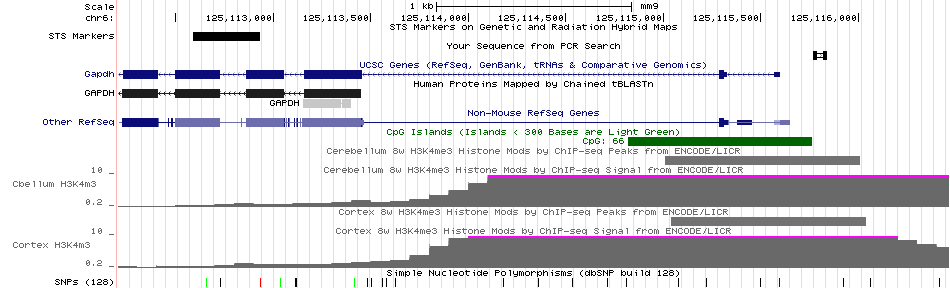
Supplementary Figures

SI2 Primer location at the glyceraldehyde-3-phosphate dehydrogenase (Gapdh) gene


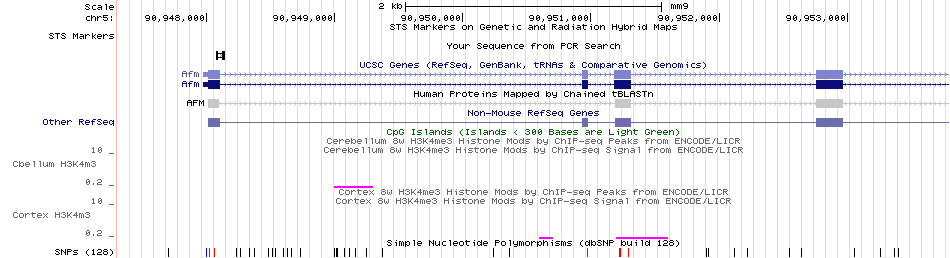


SI3 Primer location at the afamin (Afm) gene

# References

HARRIS, R. B., MITCHELL, T. D., SIMPSON, J., REDMANN, S. M., JR., YOUNGBLOOD, B. D. & RYAN, D. H. 2002. Weight loss in rats exposed to repeated acute restraint stress is independent of energy or leptin status. *Am J Physiol Regul Integr Comp Physiol,* 282**,** R77–88.

HARRIS, R. B., PALMONDON, J., LESHIN, S., FLATT, W. P. & RICHARD, D. 2006. Chronic disruption of body weight but not of stress peptides or receptors in rats exposed to repeated restraint stress. *Horm Behav,* 49**,** 615–25.

JEONG, J. Y., LEE, D. H. & KANG, S. S. 2013. Effects of chronic restraint stress on body weight, food intake, and hypothalamic gene expressions in mice. *Endocrinol Metab (Seoul),* 28**,** 288–96.

RYBKIN, II, ZHOU, Y., VOLAUFOVA, J., SMAGIN, G. N., RYAN, D. H. & HARRIS, R. B. 1997. Effect of restraint stress on food intake and body weight is determined by time of day. *Am J Physiol,* 273**,** R1612–22.

UNTERGASSER, A., CUTCUTACHE, I., KORESSAAR, T., YE, J., FAIRCLOTH, B. C., REMM, M. & ROZEN, S. G. 2012. Primer3--new capabilities and interfaces. *Nucleic Acids Res,* 40**,** e115.

1. <https://genome.ucsc.edu/cgi-bin/hgPcr> [↑](#footnote-ref-1)
